# SMART 2.0 Statistical Metabolomics Analysis: An R Tool 2.0

**DOI:** 10.1021/acs.analchem.5c03225

**Published:** 2025-10-31

**Authors:** Yu-Jen Liang, Chih-Ting Yang, Chia-Wei Chen, Yin-Chun Lin, Shu-Yao Lin, Yi Sheng Wang, Hsin-Chou Yang

**Affiliations:** 1 Institute of Statistical Science, 38017 Academia Sinica, Taipei 115, Taiwan; 2 Agricultural Biotechnology Research Center, 38017 Academia Sinica, Taipei 115, Taiwan; 3 Department of Chemistry, National Sun Yat-sen University, Kaohsiung 804, Taiwan; 4 Genomics Research Center, 38017 Academia Sinica, Taipei 115, Taiwan; 5 Biomedical Translation Research Center, 38017 Academia Sinica, Taipei 115, Taiwan; 6 Department of Statistics, National Cheng Kung University, Tainan 701, Taiwan; 7 Institute of Public Health, National Yang Ming Chiao Tung University, Taipei 11221, Taiwan

## Abstract

Metabolomics has experienced significant growth and increased popularity due to technological advancements. We introduced an integrated tool for untargeted metabolomics analysis, SMART 1.0, that streamlined the entire analysis process, from initial data preprocessing to subsequent association analysis. With SMART 2.0, we enhanced SMART 1.0 by introducing new analytical modules in targeted metabolomics analysis, data normalization, quality control assessment, and advanced dimensionality reduction and classification methods. Additionally, SMART 2.0 offers integrative omics pathway analysis and postanalysis tasks such as peak identification and concentration calibration. We also explored the potential of using large language models for peak annotation and have found the results to be promising. This study employs narcotics data and breast cancer data as demonstrative examples to illustrate the new functionalities. The codes, a user guide, and example data can be downloaded at https://github.com/YuJenL/SMART.

## Graphical abstract

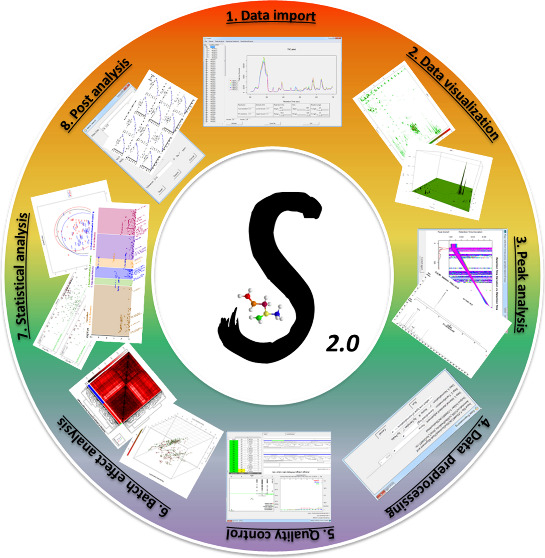

## Introduction

Metabolomics has been widely applied to characterize small molecules both intra- and intercellularly and to analyze dynamic molecular changes in response to physiological stimuli and environmental exposures in healthy and disease states.^1,2^ Metabolomics encompasses two primary study designs that lead to untargeted and targeted analyses, each with its respective strengths and limitations.^3,4^ Untargeted metabolomics is a hypothesis-generating approach that provides a global overview of metabolomics profiling. In contrast, targeted metabolomics is a hypothesis-driven approach, focusing on predefined metabolites or specific metabolic pathways.^5,6^ Recently, both untargeted and targeted metabolomics have been increasingly applied across various fields, including clinical research,^7,8^ food chemistry,^9,10^ toxicology,^11,12^ cancer study,^13–16^ COVID-19 infections,^17,18^ and pathway analysis.^19,20^ This growing trend advocates for integrating untargeted and targeted analyses simultaneously in a unified metabolomics analytical platform.^4,6,21–28^

In parallel, gaining a deeper understanding of the biological connectivity among phenotype-associated metabolites identified through metabolome-wide association studies (MWASs) is essential. However, metabolite data alone may not fully elucidate the complex biological and molecular mechanisms underlying the phenotypes of interest. To achieve a more holistic perspective, it is crucial to integrate metabolomics with other omics layers, such as genomics, epigenomics, transcriptomics, proteomics, and microbiomics, which can complement one another.^29–31^ This knowledge gap has driven us to develop a novel multiomics pathway analysis approach that combines diverse biological data with established pathway databases, including KEGG,^32^ Reactome,^33^ and WikiPathways,^34^ to offer a more complete understanding of the biological mechanisms underlying health and disease.^29,35^

Numerous metabolomics analysis tools are available, including commercial platforms from major instrument manufacturers (e.g., Waters’ Progenesis QI, Bruker’s MetaboScape, Thermo Fisher’s Xcalibur, and Agilent’s MassHunter) and open-source tools developed by the research community (e.g., XCMS,^36^ MZmine,^37^ OpenMS,^38^ and MetaboAnalyst^39^). While each offers valuable functionalities, there remains a need for a one-stop, user-friendly, comprehensive, and flexible solution that integrates and extends their capabilities.

SMART 1.0 was developed as an integrated tool for untargeted metabolomics data analysis, streamlining the entire analysis workflow from initial data preprocessing to downstream association analysis.^40^ It was designed as a one-stop, user-friendly, comprehensive, and flexible solution and has contributed to advancing metabolomics research. While SMART 1.0 effectively addressed many key analytical needs, the evolving demands of the field and valuable user feedback highlighted opportunities to further broaden its capabilities. These considerations motivated the development of SMART 2.0, which builds upon the strong foundation of SMART 1.0.

In SMART 2.0, we significantly enhance the capacities of SMART 1.0 by introducing advanced analytical modules such as targeted data analysis, omics data integration, and concentration calibration. We also expand functionalities for data preprocessing, quality control, and postanalysis procedures. These improvements considerably broaden the scope and applicability of metabolomics research. The R source codes, user guide, and example data sets for SMART 2.0 are available for download from the GitHub repository at https://github.com/YuJenL/SMART.

## Materials and Methods

### SMART 2.0 Description

SMART 2.0, featuring a user-friendly interface, was developed in R for the Windows and Linux operating systems. SMART 2.0 expands upon the analysis modules in SMART 1.0, introducing several new features. These include peak analysis for targeted data; normalization methods for data preprocessing, such as Pareto scaling (PS)^41^ and rank-based inverse normal transformation (RINT);^42^ signal-to-noise ratio (S/N) calculation for quality control; partial least-squares and partial least-squares discriminant analysis (PLS/PLS-DA);^43–45^ integrative omics pathway analysis (IOPA); and peak identification and concentration calibration^43,46^ in the postanalysis stage. The modules are introduced in the following subsections according to the workflow shown (Figure 1). The differences between SMART 1.0 and 2.0 modules are highlighted (Figure S1), and new functionalities are illustrated in the following subsections.

**Figure 1:**
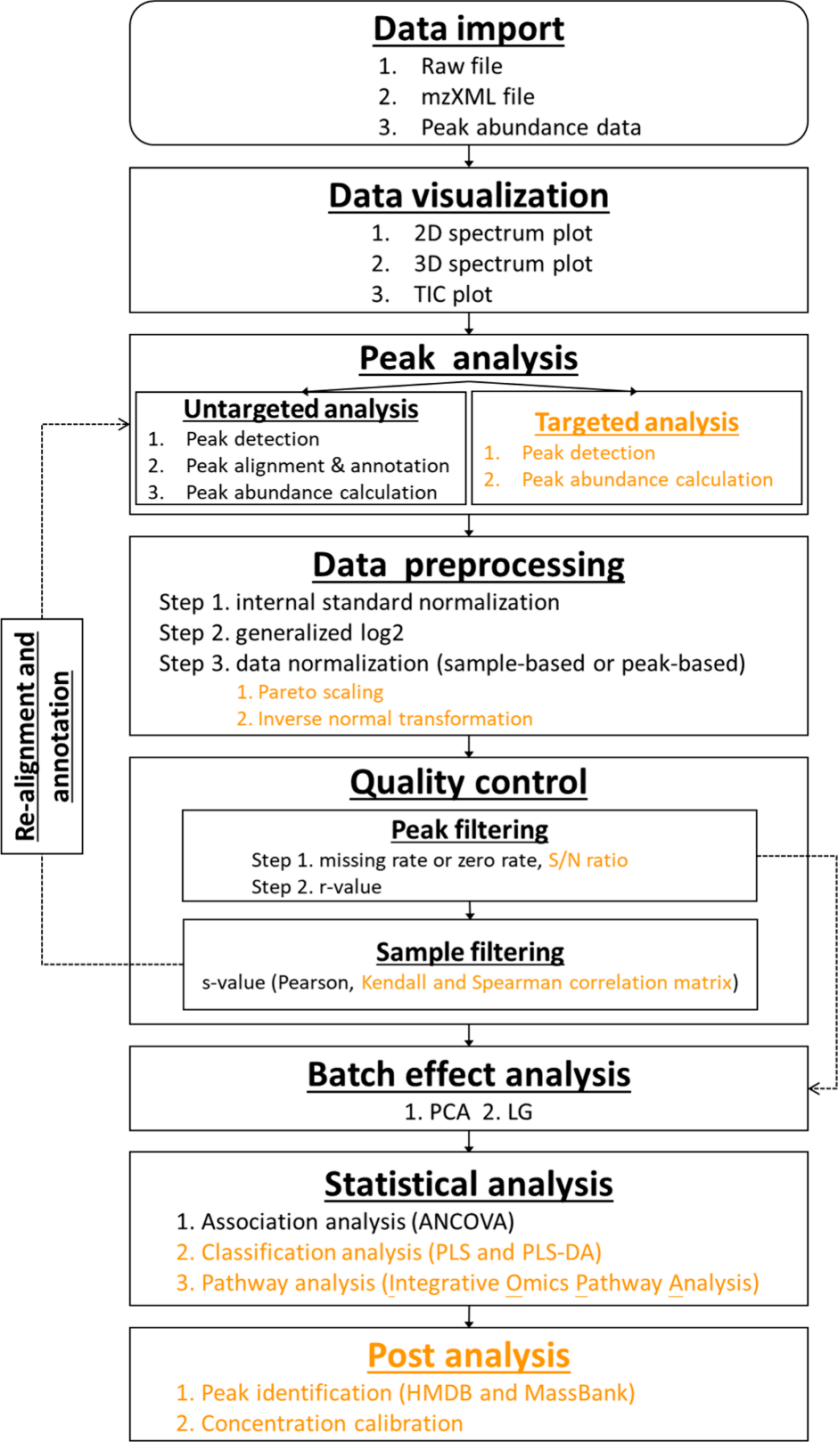
Analytical flowchart of SMART 2.0. The primary modules are indicated in bold, with newly introduced modules and methods highlighted in orange.

### Peak Analyses

SMART 1.0 provides only untargeted peak detection and retention time (RT) alignment using XCMS.^36^ In SMART 2.0, we further develop a dedicated workflow for peak detection and peak abundance calculation in targeted data experiments. The detailed protocol is outlined in the Supporting Information “Peak analysis” (Text S1).

#### Peak Detection for Targeted Experiments

LC-MS and LC-MS/MS experimental results (*m*/*z* values and RT) can be provided for peak detection. Precise *m*/*z* values for MS1 or MS2 should be included, while RT information may be omitted. If the RT is not provided, then SMART 2.0 will automatically scan all MS1 spectra to identify the peak with the precise *m*/*z* value and assign its RT. Furthermore, if the MS2 experimental result is available, then SMART 2.0 will search for MS2 spectrum fragments using either the RT information supplied by the user for the target peaks or the RT acquired from MS1. Subsequently, SMART 2.0 will compare the detected MS2 fragments with their true *m*/*z* values, allowing for a specified margin of error. We can identify these target peaks using the MS1 and MS2 data at this stage.

#### Peak Abundance Calculation for Targeted Experiments

The peak abundance calculation utilizes the *m*/*z* and RT values detected by the peak detection algorithm to accumulate the peak intensity, where the ranges of *m*/*z* and RT are user-provided. The peak abundance tables for MS1 and MS2 will be output separately. If a fragment is not detected, then it will be output as not available (NA). The module also offers the calculation of the S/N for quality control of fragments, where SNR is calculated as the mean intensity divided by the standard deviation of intensity. Furthermore, in addition to the peak abundance table, the module calculates the relative abundance of each fragment based on the results from MS2. Additionally, the MS1 and MS2 analysis spectra for each peak will also be provided as output.

### Data Preprocessing

In SMART 1.0, we provided three procedures for peak abundance adjustment: an internal standard, data transformation, and data normalization. To better accommodate a broader range of metabolomics data characteristics, such as high variance and non-normally distributed metabolite abundances, SMART 2.0 introduces additional scaling and normalization options in the data preprocessing module, including PS^41^ and RINT.^42^ These two methods were specifically chosen for their complementary strengths: PS reduces the influence of large fold changes while preserving the data structure, and RINT helps stabilize variance and approximate normality, facilitating downstream statistical analysis. PS is particularly suitable for metabolite data analysis because it normalizes variability in metabolite concentrations, enhances data interpretation, balances the S/N, facilitates multivariate analysis, and retains biological variance. This scaling method improves the robustness and accuracy of statistical techniques used in metabolomics.^41,47–49^ The inverse normal transformation, commonly used in genome-wide association studies (GWASs), quantitative trait locus (QTL) mapping, and clinical studies for non-normally distributed phenotypes,^42,50,51^ is valuable in metabolomics data analysis. It normalizes data, meets statistical assumptions, reduces the impact of outliers, clarifies metabolite relationships for principal component analysis (PCA) and PLS-DA, and standardizes data scales for better comparison and comprehensive analysis.^52,53^

### Quality Control

#### Peak Filtering

In SMART 1.0, we implemented missing rate and zero rate calculations as basic quality control measures to filter suboptimal peaks. These metrics primarily capture the completeness and presence/absence of data for each peak across samples. However, they do not sufficiently account for peak intensity variability across samples, which can indicate biological instability or technical inconsistency.

To further account for variability in peak intensity among samples, SMART 2.0 introduces the S/N as an additional filtering criterion. The S/N ratio reflects the consistency and reliability of peak detection by comparing the signal intensity to background noise, thus capturing not only presence/absence but also sample-level variability in peak abundance. This allows for the identification of peaks with excessive variability or poor reproducibility, even if their missing or zero rates are acceptable.

By integrating the S/N ratio, SMART 2.0 provides a more comprehensive and robust framework for peak quality assessment, enabling users to better filter out unstable or noisy peaks. Users can specify an S/N threshold to exclude such peaks from downstream analysis, thereby enhancing the overall data quality and reliability of statistical inference.

#### Sample Filtering

In SMART 1.0, sample filtering was based on distance metrics derived from clustering analysis, specifically using Pearson’s correlation matrix to calculate intersample distances. In SMART 2.0, we have augmented this approach by incorporating additional methods for correlation matrix computation, including Kendall and Spearman correlations. Pearson’s correlation assesses linear relationships, while Spearman’s and Kendall’s correlations evaluate monotonic and ordinal relationships, respectively. This expanded methodological framework allows for more comprehensive analysis across diverse data scenarios.

### Statistical Analysis

#### PLS/PLS-DA

SMART 1.0 provided a general analysis of covariance (ANCOVA) model for each peak to discover the association between metabolites and the main factor groups (e.g., case vs control) or quantitative traits of interest (e.g., blood pressure). In SMART 2.0, we further utilized the ropls package^54^ to construct PLS and PLS-DA models, enabling the identification of associations between metabolites and multiple continuous or categorical traits through latent variable analysis. Each latent variable is a linear combination of the original independent variables, retaining the most important patterns and relationships. In addition, to account for the influence of covariates, we also generate residuals adjusted for covariates using the ANCOVA model and include them in the PLS and PLS-DA models. Subsequently, we will compute the PLS scores and the variable importance in projection (VIP) scores. The VIP score indicates a variable’s significance within the PLS and PLS-DA models, encapsulating a variable’s contribution to the overall model. Additionally, the proportion of variance in the response variable *Y* explained by the model (*R2Y*) and predictive accuracy of the model (*Q2Y*) values will be generated to assess the model’s performance.

#### Integrative Omics Pathway Analysis (IOPA)

SMART 2.0 adds a pathway analysis module for interpreting biological processes. Various pathway-based analysis algorithms exist,^55^ including over-representation analysis (ORA),^56^ gene set analysis (GSA),^57^ gene set enrichment analysis (GSEA),^58^ and signaling pathway impact analysis (SPIA).^59^ Among these, SPIA incorporates pathway topology, making it a widely recognized informative method for pathway analysis. However, SPIA was initially developed for analyzing single-type molecular data. We extend SPIA to consider both genes and metabolites in KEGG metabolism pathways jointly,^32,60,61^ and we name this extension eSPIA.

SMART 2.0 integrates multiomics data, e.g., transcriptomics integrated with metabolomics or genomics integrated with metabolomics, based on the KEGG pathway database. KEGG pathway information is retrieved using the R package KEGGREST,^62^ which provides access to the KEGG REST API (Representational State Transfer Application Programming Interface).^63^ Pathway structures are parsed using the R package KEGGgraph,^64^ and finally, gene IDs, compound IDs, and relations between compounds and genes are obtained for each pathway. A detailed protocol refers to the Supporting Information “Integrative omics pathway analysis” (Text S2).

To identify the phenotype-associated pathways, SMART 2.0 first calculates *p*-values for each pathway using both ORA and eSPIA. These *p*-values are then integrated using either Fisher’s method,^65^ which assumes independence among *p*-values, or the Pbine method^66^ (available at https://github.com/Yinchun-Lin/Pbine), which relaxes this independence assumption. Depending on the data characteristics and analysis objectives, either Fisher’s method or the Pbine method can be used. Finally, multiple testing is addressed through false discovery rate^67^ (FDR) adjustment and Bonferroni correction.^68^ The flowchart of IOPA is shown (Figure 2).

**Figure 2:**
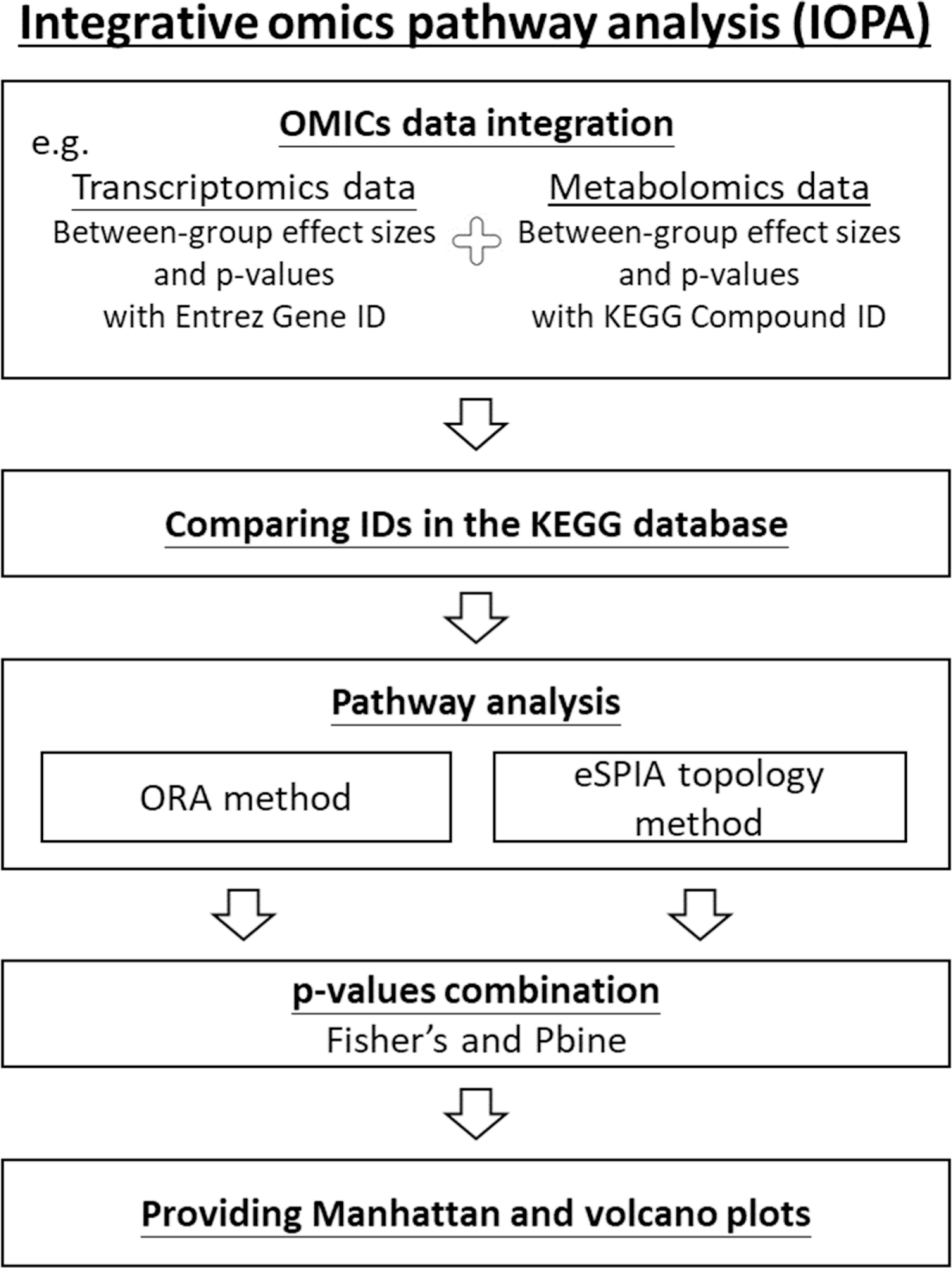
Flowchart of IOPA. The main analysis process of IOPA involves collecting summary statistics (e.g., between-group effect sizes and *p*-values) from omics studies, mapping gene and compound IDs to KEGG pathways, calculating *p*-values for each pathway by using ORA and eSPIA separately, and applying *p*-value combination methods, such as Fisher’s method and Pbine, to evaluate the statistical significance of pathways, display Manhattan and volcano plots, and identify those pathways that are significant.

### Postanalysis

#### Peak Identification

To identify significant peaks obtained from ANCOVA and PLS/PLS-DA analyses, SMART 2.0 provides a convenient batch query function that matches peaks against the HMDB^69–71^ and MassBank^72^ databases. Users should provide query information, including the mass (Da) of peaks, the ion mode (neutral, positive, and negative modes), adduct types, and tolerance (Da or ppm). The HMDB metabolite data set was downloaded from the Human Metabolome Database (https://hmdb.ca/downloads), and the MassBank metabolite data set was downloaded from the MassBank repository (https://github.com/MassBank/MassBank-data/tags). Both data sets are periodically updated in SMART 2.0 to incorporate the latest curated information, ensuring reliable and reproducible metabolite annotation during analysis. SMART 2.0 returns a table listing the target *m*/*z*, adduct type, database name, database IDs (HMDB ID, MassBank Accession, and KEGG ID), metabolite name, chemical formula, molecular weight, and delta (i.e., the difference between target *m*/*z* and adduct *m*/*z*). With recent advancements in generative artificial intelligence, SMART 2.0 also explores the potential of using large language models (LLMs) for enhanced peak identification. Although LLM access is currently conducted externally via APIs, we provide example prompts and workflows in the Supporting Information “Post analysis (Peak identification)” (Text S3), with illustrative examples shown in the Results section (Table 3). Integration of LLM functionalities is part of our future development roadmap to further expand AI-assisted capabilities within SMART.

#### Concentration Calibration

To calibrate the concentration of a compound in a test sample, a calibration curve is first constructed based on a standard compound with known abundances at different concentrations.^73–75^ SMART 2.0 implements a two-step procedure for concentration calibration: (1) constructing a calibration curve and (2) estimating the concentration of unknown samples using the established curve. Calibration curve construction can be performed using a linear or quadratic regression model, with optional weighting factors of 1, 1/*x*, or 1/*x* ^2^. The optimal weighting scheme is determined based on the relationship between the concentrations (*x*) and the corresponding standard deviation of instrument responses (σ).^76,77^ To ensure robustness, outlier detection is conducted using Cook’s distance (Cook’s *D*), confidence intervals (CI), and prediction error (i.e., the discrepancies between the true and estimated concentrations). Outliers are removed, and the mode is refitted to improve the model’s accuracy and reliability. The quality of each calibration model is assessed using the coefficient of determination *R* ^2^ and adjusted *R* ^2^. All model results under various conditions are provided, allowing users to select the most appropriate calibration line. Once the optimal calibration curve is established, the concentration calibration module can compute the concentration of test or other unknown samples. A flowchart illustrating the concentration calibration process is provided (Figure 3). For a detailed protocol, please refer to the Supporting Information “Post analysis (Concentration calibration)” (Text S4).

**Figure 3:**
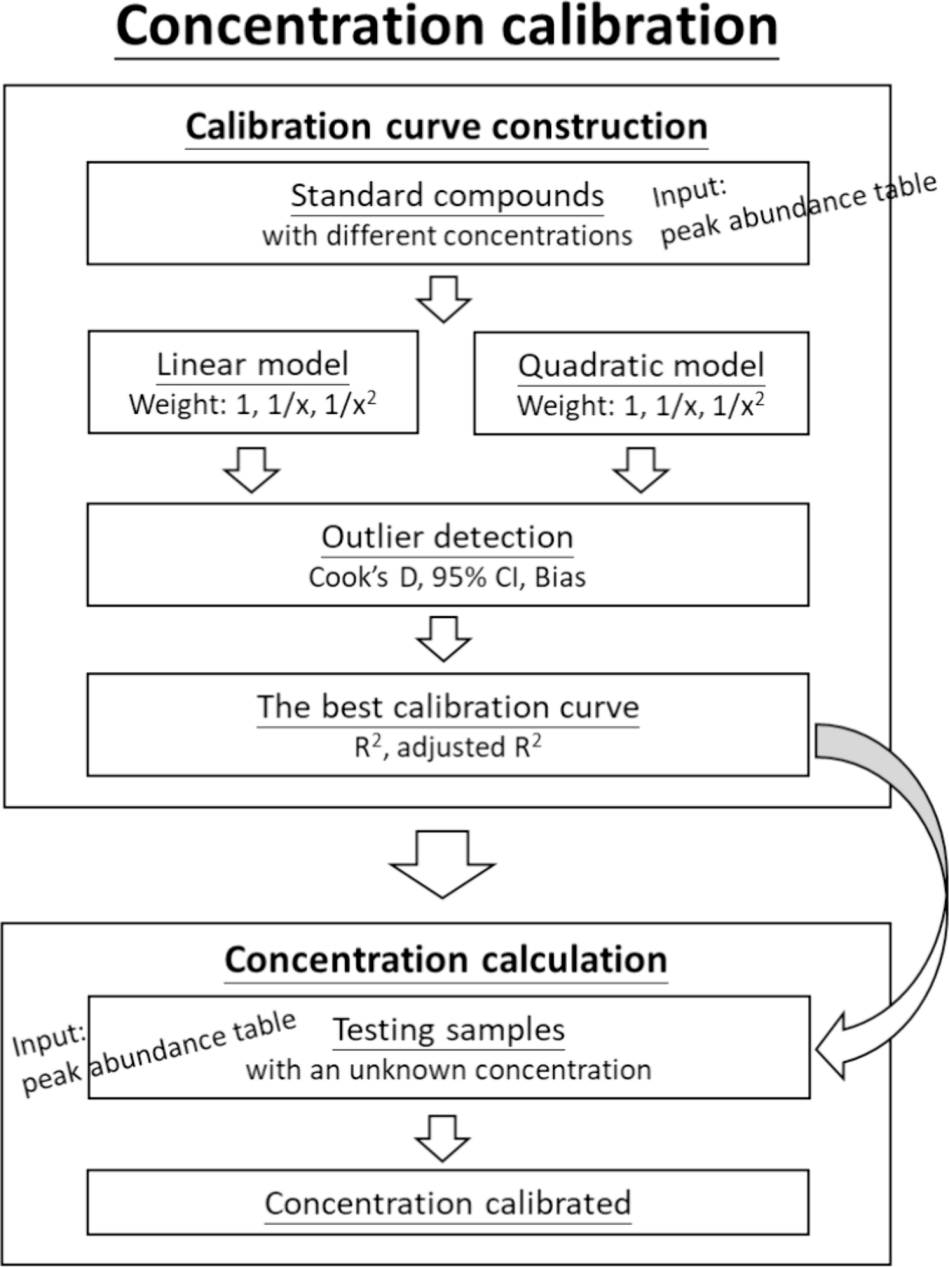
Flowchart of concentration calibration. The concentration calibration process consists of two main steps: calibration curve construction and concentration estimation. First, calibration curves are built for the peak abundance data of each standard compound across different concentrations. Both linear and quadratic models are considered, along with three weighting schemes (1, 1/*x*, and 1/*x* ^2^). Outlier detection is then performed using indicators: Cook’s distance, 95% confidence interval, and bias. The best-fit calibration line is selected based on *R* ^2^ and adjusted *R* ^2^ values. In the second step, the concentration of the unknown sample is estimated by substituting its peak abundance values into the corresponding calibration curve derived in the first step.

## Results

### Two Data Sets for Demonstration

#### Narcotics Data

According to official statistics released by Taiwan’s Ministry of the Interior, a total of 37,854 individuals were implicated in drug-related offenses in 2024 (https://statis.moi.gov.tw/micst/webMain.aspx?k=menume). The total quantity of narcotic substances confiscated amounted to 11,294.77 kg, of which more than 65% were categorized as Schedule III or higher controlled substances under national drug classification standards. Drug abuse poses serious public health challenges, leads to violence and crime, and imposes significant burdens on families and society.

To demonstrate the targeted analysis capabilities of SMART 2.0, we executed a new experiment focusing on 12 commonly used first- and second-level narcotics in Taiwan. LC-MS and LC-MS/MS experiments were conducted on a set of 12 narcotics, which included heroin (diacetylmorphine), morphine, cocaine, thebaine, delta9-THC (delta9-tetrahydrocannabinol), amphetamine, MA (methamphetamine), MDMA (3,4-methylenedioxymethamphetamine), MDA (also known as the “love drug”; 3,4-methylenedioxyamphetamine), ketamine, FM2 (flunitrazepam), and nimetazepam. Each of the 12 narcotics was first prepared at 11 distinct concentration levels (i.e., 50, 100, 200, 300, 400, 500, 600, 700, 800, 900, and 1000 ppb). Then, for each concentration level, equal volumes of the 12 individual narcotic solutions were combined to create one mixture solution. As a result, each mixture sample at a given concentration level contained all 12 narcotics at the same concentration. Finally, for each mixture sample, we conducted independent mass spectrometry analyses focusing on one narcotic at a time.

The 500 ppb sample was designated as the test sample for concentration calibration, whereas the remaining samples at the other 10 concentration levels were used to construct the calibration curve. A detailed description of the experiment can be found in the Supporting Information “Drug experiment” (Text S5). The data presented here were first published and analyzed in the current paper. This data set was used to evaluate the performance of SMART 2.0 across three key tasks: targeted peak analysis, metabolite identification, and concentration calibration.

#### Breast Cancer Data

A previous breast cancer study conducted gene expression and metabolomics experiments.^78,79^ The metabolomics data set comprised 536 metabolites measured across 132 participants, including 67 with human breast tumor tissue samples and 65 with tumor-adjacent noncancerous tissue samples. Regarding racial distribution, the cohort comprised 64 African-American individuals (32 tumor and 32 control samples) and 68 European-American individuals (35 tumor and 33 control samples).

The gene expression data set included 20,254 genes across 108 individuals, of whom 61 provided tumor samples and 47 provided adjacent nontumor tissue samples. Within this data set, there were 53 individuals of African-American descent (including 30 tumor and 23 control samples) and 55 individuals of European-American descent (including 31 tumor and 24 control samples).

For demonstration purposes, we acquired the gene expression and metabolomics data directly from the publicly available repository associated with the integrative R package IntLIM on GitHub (https://github.com/mathelab/IntLIM).^79^ We applied this multiomics data set to demonstrate the functionalities of the PLS-DA and IOPA modules in SMART 2.0.

### SMART 2.0 Software

The SMART 2.0 software, user guide, and illustrative examples can be downloaded from the GitHub repository (https://github.com/YuJenL/SMART). Demonstration results using the narcotics and breast cancer data sets are presented below.

### Peak Analysis: Targeted Data

We utilized the narcotics data set to demonstrate this functionality. For the peak analysis of the 12 targeted narcotics, the *m*/*z* tolerance was set at 1000 ppm, and the RT tolerance was ±5 s. A summary of the successful detection of all 12 targeted narcotics across 11 different concentration levels is provided (Table 1). For instance, the estimated *m*/*z* value for heroin in MS1 across 11 concentration levels was 370.16, with an associated RT of 11.42 min. In MS2, the estimated *m*/*z* values for heroin across 11 different concentrations were 370.16, 328.15, 268.13, 211.07, 193.06, and 58.00, with corresponding relative abundances of 100.00, 18.21, 19.30, 10.82, 4.67, and <0.01, respectively. As part of the quality assessment, we calculated the S/N ratio for each compound. For example, the average S/N ratio of heroin across the 11 concentration levels was 1.28. Additionally, Figure S2A–L displays the MS1 and MS2 spectra of the 12 narcotics at a concentration of 500 ppb. The peak analysis module ultimately generates the peak abundance tables for MS1 and MS2, covering all 12 narcotics across the 11 concentration levels, used in downstream analysis.

**Table 1:** Targeted Peak Analysis of 12 Narcotics^b^

		MS1			MS2		
name	RT (min)	true *m*/*z*	estimated *m*/*z*	S/N	true *m*/*z*	estimated *m*/*z*	relative abundance
heroin	11.42	370	370.16	1.28	370, 328, 268, 211, 193, 58	370.16, 328.15, 268.13, 211.07, 193.06, 58.00	100.00, 18.21, 19.30, 10.82, 4.67, <0.01
morphine	2.45	286	286.14	0.82	286, 268, 229, 201	286.14, 268.13, 229.09, 201.09	100.00, 2.95, 9.02, 13.00
cocaine	11.7	304	304.15	1.15	304, 182, 150, 105, 82	304.15, 182.12, 150.09, 105.03, 82.06	3.82, 100.00, 10.50, 9.19, 12.75
thebaine	11.31	312	312.16	1.32	312, 281, 266, 251, 221, 58	312.16, 281.12, 266.09, 251.07, 221.10, 58.04	34.44, 15.92, 61.18, 100.00, 61.28, 0.05
delta9-THC^a^	25.23	315	315.23	0.75	315, 259, 193, 135, 123, 107, 93	315.20, 259.17, 193.12, 135.12, 123.04, 107.09, 93.07	12.61, 32.05, 100.00, 27.85, 19.31, 14.02, 21.59
amphetamine	7.04	136	136.11	0.19	136, 119, 91	136.02, 119.08, 91.05	4.26, 0.81, 100.00
MA^a^	8.26	150	150.13	0.59	150, 119, 91	150.12, 119.09, 91.05	0.12, 1.56, 100.00
MDMA^a^	8.99	194	194.12	0.99	194, 163, 135, 133, 105	194.10, 163.07, 135.04, 133.06, 105.07	0.24, 47.83, 99.94, 76.71, 91.98
MDA^a^	8.44	180	180.10	0.86	180, 163, 135, 133, 105	180.08, 163.07, 135.04, 133.06, 105.07	0.34, 23.19, 84.90, 63.52, 100.00
ketamine	10	238	238.10	1.22	238, 220, 179, 163, 152, 125	238.10, 220.09, 179.06, 163.03, 152.03, 125.01	0.62, 15.44, 19.04, 18.86, 7.91, 100.00
FM2^a^	17.4	314	314.09	0.96	314, 200, 286, 268	314.09, 200.08, 286.10, 268.10	67.98, 0.03, 8.51, 100.00
nimetazepam	17.35	296	296.10	0.94	296, 268, 250, 222, 193, 165	296.10, 268.11, 250.11, 222.11, 193.09, 165.07	65.29, 13.04, 100.00, 11.55, 4.08, 3.93

a delta9-THC: delta9-tetrahydrocannabinol; MA: methamphetamine; MDMA: 3,4-methylenedioxymethamphetamine; MDA: 3,4-methylenedioxyamphetamine; FM2: flunitrazepam.

b The results of MS1 and MS2 analyses are presented in the respective columns. The true *m*/*z* and retention time (RT) values were known a priori (user-provided), and the *m*/*z* values are listed in descending order. The peak analysis module computed the estimated *m*/*z*, signal-to-noise ratio (S/N), and relative abundance for each compound. See the Supporting Information (“Peak analysis”) for a detailed description of the protocol. All estimated *m*/*z*, S/N, and relative abundance values represent the averages across the 11 concentration levels.

### Statistical Analysis

#### PLS/PLS-DA

We used the breast cancer data set to demonstrate the functionality of the PLS/PLS-DA module. The score plots from the PLS-DA analyses, based on gene expression or metabolomics data sets, showed a clear separation between tumor and control samples (Figure S3A–C). Using the full gene expression data set (20,254 genes), the model achieved *R2Y* = 0.685 and *Q2Y* = 0.539 (Figure S3A). For the full metabolomics data set (504 metabolites), the model yielded *R2Y* = 0.594 and *Q2Y* = 0.454 (Figure S3B). Notably, the integrative analysis combining gene expression and metabolite data further improved the separation between tumor and control samples, achieving *R2Y* = 0.694 and *Q2Y* = 0.55 (Figure S3C).

To enhance model parsimony and predictive accuracy, SMART 2.0 supports covariate adjustment, variable selection, and integration across multiple analytical modules. In this demonstration, we compared different submodels by selecting only the most relevant gene expression and metabolite variables (Tables S1 and S2). First, ANCOVA was performed separately on the gene expression and metabolomics data with adjustment for age and ancestry. The resulting residuals were then directly used as input to the PLS/PLS-DA module within SMART 2.0. This highlights a key advantage of the platform: users can perform covariate adjustment and downstream multivariate modeling in a seamless pipeline without switching to external software or reformatting intermediate results.

ANCOVA identified 9227 genes and 343 metabolites significantly associated with breast cancer (pFDR < 0.05). Several variable selection strategies were evaluated to prioritize features based on the smallest *p*-values or highest variable importance in projection (VIP) score (Table S1). The results indicated that a dual-filtering strategy, which combines pFDR < 0.05 with top 10 VIF scores, markedly enhanced predictive accuracy. This optimized model achieved the highest predictive accuracy (*Q2Y* = 0.701), along with robust performance *R2Y* = 0.725 and a low root-mean-square error of estimation (RMSEE) of 0.263 (Figure 4). The top 10 most important gene expression and metabolite variables included in this model are provided (Table S2). These findings highlight the advantage of integrating statistical significance with multivariate relevance, as well as the efficiency and flexibility of SMART 2.0 for streamlined multiomics modeling.

**Figure 4:**
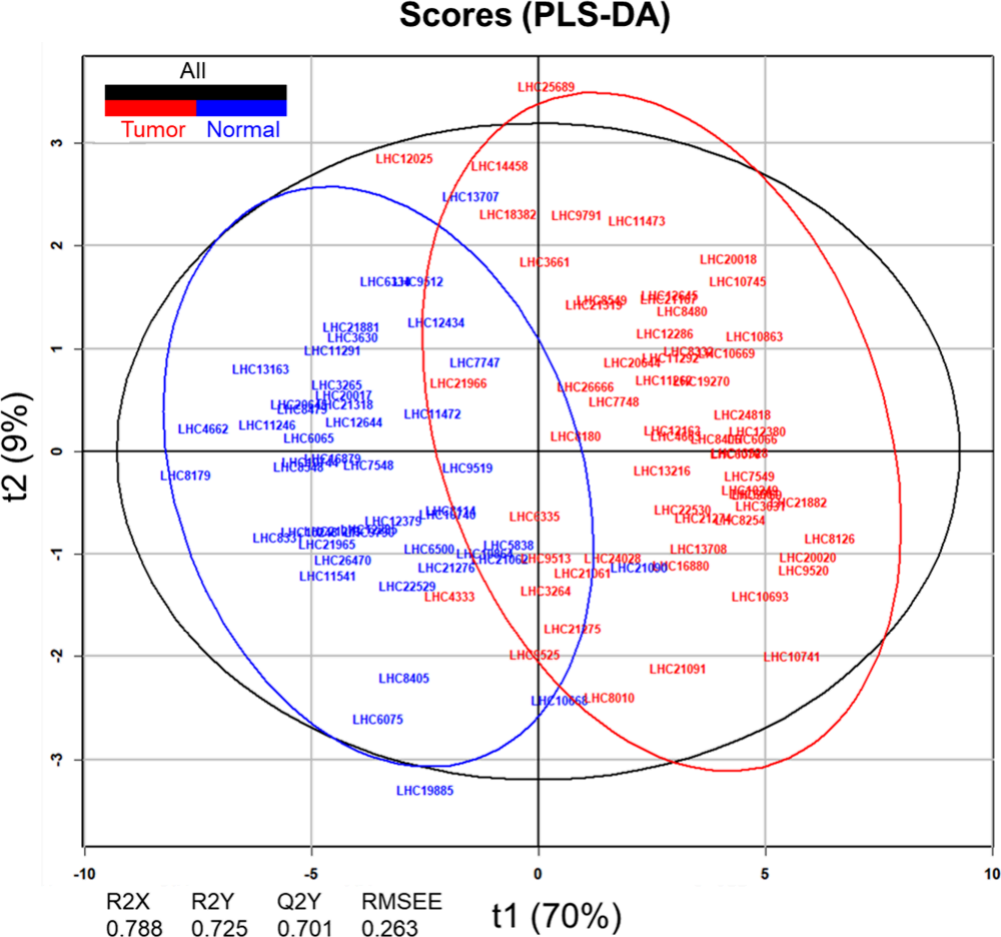
Score plot from the PLS-DA analysis of breast cancer data. Using the dual-filtering strategy, which combines pFDR < 0.05 with top 10 VIF scores, the optimized model achieved the highest predictive accuracy *Q2Y* = 0.701, along with robust performance in *R2Y* = 0.725 and a low root-mean-square error of estimation (RMSEE) = 0.263. *R2X* and *R2Y* indicate the proportion of variance explained in the predictors (*X*) and response (*Y*), respectively. *Q2Y* reflects the model’s predictive accuracy via cross-validation, while RMSEE quantifies the model’s estimation error. The plot displays the first two latent variables: *t*1, explaining 70% of the total variation, and *t*2, explaining 9%. Red dots represent breast cancer samples, and blue dots indicate normal controls. Red, blue, and black ellipses represent 95% confidence intervals for breast cancer, normal control, and overall groups, respectively.

#### IOPA

We also analyzed the breast cancer data set to demonstrate the functionality of IOPA. First, this analysis focused on 19,675 of 20,254 genes with Entrez Gene IDs and 239 of 504 metabolites with KEGG Compound IDs. ANCOVA identified 9036 genes and 199 metabolites significantly associated with breast cancer. Second, we retrieved 347 KEGG pathways using the R package KEGGREST and successfully parsed all of them with the R package KEGGgraph. Third, based on the beta coefficients, i.e., between-group differences or effect size, obtained from ANCOVA, we applied a threshold of >0.005 or < −0.005 to identify differentially regulated features. Using this criterion, we detected 51 pathways via ORA (pFDR < 0.05) and two pathways via eSPIA (pFDR < 0.05). Finally, we identified 34 significant pathways using Pbine (pFDR < 0.05). The detailed results are presented in Table 2. Among them, the PI3K-Akt signaling pathway (hsa04151) exhibited the highest significance (pPbine_FDR = 2.80 × 10^–10^), with a pathway of 350 genes and 53 significant genes. Figure 5 represents a Manhattan plot summarizing the IOPA results across six categories of KEGG pathway maps. Of the 34 significant pathways, six were related to metabolism and 13 were associated with human diseases. A volcano plot depicting pathway significance versus normalized eSPIA scores is shown (Figure S4). Notably, the fructose and mannose metabolism pathway was significant and had the highest normalized eSPIA score of 0.458.

**Figure 5:**
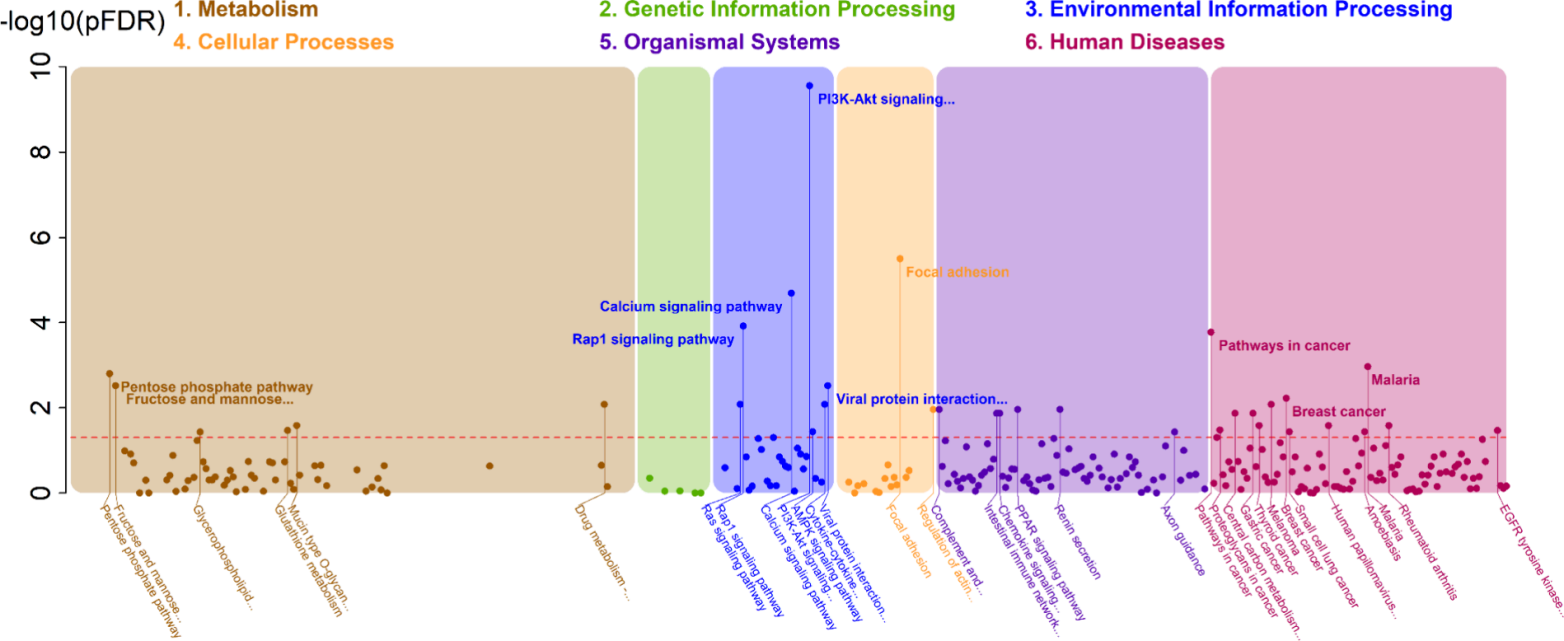
Manhattan plot from the IOPA analysis of breast cancer data. The colors on the plot correspond to the KEGG pathway maps. The vertical axis represents the significance of the Pbine test, with the FDR-adjusted *p*-values displayed as their negative logarithms (−log 10). The horizontal axis represents pathways from the KEGG database. The horizontal reference line indicates the significance level (α = 0.05). Names of the top 10 significant pathways are displayed in bold.

**Table 2:** 34 Significant Pathways Identified through the IOPA^b^

		gene			compound			pathway (gene + compound)					
**name**	**ID**	**G_n**	**deG_n**	**G_pORA**	**C_n**	**deC_n**	**C_pORA**	**P_n**	**deP_n**	**pORA**	**peSPIA**	**pPbine**	**pPbine_FDR**
PI3K-Akt signaling pathway^a^	hsa04151	349	53	4.820 × 10^–11^	1	0	1	350	53	4.272 × 10^–10^	5.000 × 10^–6^	1.032 × 10^–12^	2.800 × 10^–10^
focal adhesion	hsa04510	199	33	2.510 × 10^–8^	0	0	1	199	33	9.642 × 10^–8^	2.000 × 10^–3^	2.301 × 10^–8^	3.120 × 10^–6^
calcium signaling pathway	hsa04020	239	28	2.160 × 10^–4^	0	0	1	239	28	5.388 × 10^–4^	5.000 × 10^–6^	2.220 × 10^–7^	1.550 × 10^–5^
Rap1 signaling pathway	hsa04015	209	30	2.520 × 10^–6^	0	0	1	209	30	7.807 × 10^–6^	4.000 × 10^–3^	1.806 × 10^–6^	1.200 × 10^–4^
pathways in cancer^a^	hsa05200	529	55	1.010 × 10^–5^	4	0	1	533	55	6.001 × 10^–5^	1.000 × 10^–3^	3.152 × 10^–6^	1.700 × 10^–4^
malaria	hsa05144	49	14	3.350 × 10^–7^	0	0	1	49	14	6.671 × 10^–7^	1	2.435 × 10^–5^	1.099 × 10^–3^
pentose phosphate pathway	hsa00030	29	5	2.202 × 10^–2^	8	6	2.009 × 10^–02^	37	11	6.891 × 10^–6^	1.820 × 10^–1^	4.150 × 10^–5^	1.606 × 10^–3^
fructose and mannose metabolism	hsa00051	33	4	1.141 × 10^–1^	7	6	6.934 × 10^–03^	40	10	9.345 × 10^–5^	3.900 × 10^–2^	1.018 × 10^–4^	3.039 × 10^–3^
viral protein interaction with cytokine and cytokine receptor	hsa04061	96	18	6.360 × 10^–6^	0	0	1	96	18	1.385 × 10^–5^	2.510 × 10^–0^	9.787 × 10^–5^	3.039 × 10^–3^
breast cancer^a^	hsa05224	147	18	1.661 × 10^–3^	0	0	1	147	18	3.078 × 10^–3^	3.000 × 10^–3^	2.218 × 10^–4^	6.007 × 10^–3^
drug metabolism-cytochrome P450	hsa00982	59	12	9.630 × 10^–5^	0	0	1	59	12	1.650 × 10^–4^	1.200 × 10^–1^	4.189 × 10^–4^	8.375 × 10^–3^
Ras signaling pathway	hsa04014	232	25	1.543 × 10^–3^	0	0	1	232	25	3.295 × 10^–3^	6.000 × 10^–3^	4.185 × 10^–4^	8.375 × 10^–3^
cytokine-cytokine receptor interaction	hsa04060	287	35	1.600 × 10^–5^	0	0	1	287	35	5.184 × 10^–5^	3.970 × 10^–1^	4.327 × 10^–4^	8.375 × 10^–3^
melanoma	hsa05218	72	11	2.296 × 10^–3^	0	0	1	72	11	3.563 × 10^–3^	5.000 × 10^–3^	3.837 × 10^–4^	8.375 × 10^–3^
PPAR signaling pathway	hsa03320	72	14	4.380 × 10^–5^	0	0	1	72	14	8.103 × 10^–5^	4.400 × 10^–1^	6.828 × 10^–4^	1.097 × 10^–2^
regulation of the actin cytoskeleton	hsa04810	215	28	3.390 × 10^–5^	0	0	1	215	28	9.123 × 10^–5^	3.770 × 10^–1^	6.627 × 10^–4^	1.097 × 10^–2^
renin secretion	hsa04924	69	12	4.500 × 10^–4^	2	1	5.661 × 10^–01^	71	13	2.707 × 10^–4^	1.330 × 10^–1^	6.882 × 10^–4^	1.097 × 10^–2^
complement and coagulation cascades	hsa04610	85	16	1.950 × 10^–5^	0	0	1	85	16	3.904 × 10^–5^	9.960 × 10^–1^	7.336 × 10^–4^	1.104 × 10^–2^
chemokine signaling pathway	hsa04062	188	25	6.200 × 10^–5^	0	0	1	188	25	1.524 × 10^–4^	3.890 × 10^–1^	1.040 × 10^–3^	1.368 × 10^–2^
intestinal immune network for IgA production	hsa04672	45	10	1.680 × 10^–4^	0	0	1	45	10	2.676 × 10^–4^	2.400 × 10^–1^	1.111 × 10^–3^	1.368 × 10^–2^
gastric cancer^a^	hsa05226	149	19	7.660 × 10^–4^	0	0	1	149	19	1.495 × 10^–3^	4.100 × 10^–2^	1.069 × 10^–3^	1.368 × 10^–2^
central carbon metabolism in cancer^a^	hsa05230	70	9	1.685 × 10^–2^	26	5	9.764 × 10^–01^	96	14	1.703 × 10^–3^	3.200 × 10^–2^	9.698 × 10^–4^	1.368 × 10^–2^
thyroid cancer^a^	hsa05216	37	9	1.700 × 10^–4^	0	0	1	37	9	2.615 × 10^–4^	6.030 × 10^–1^	2.323 × 10^–3^	2.634 × 10^–2^
rheumatoid arthritis	hsa05323	88	14	4.050 × 10^–4^	0	0	1	88	14	7.139 × 10^–4^	2.220 × 10^–1^	2.333 × 10^–3^	2.634 × 10^–2^
mucin-type *O-*glycan biosynthesis	hsa00512	34	8	4.990 × 10^–4^	0	0	1	34	8	7.282 × 10^–4^	2.420 × 10^–1^	2.544 × 10^–3^	2.652 × 10^–2^
human papillomavirus infection	hsa05165	328	36	1.140 × 10^–4^	0	0	1	328	36	3.415 × 10^–4^	4.970 × 10^–1^	2.467 × 10^–3^	2.652 × 10^–2^
proteoglycans in cancer^a^	hsa05205	203	25	2.140 × 10^–4^	0	0	1	203	25	5.006 × 10^–4^	4.910 × 10^–1^	3.338 × 10^–3^	3.350 × 10^–2^
glutathione metabolism	hsa00480	56	5	2.095 × 10^–1^	11	6	1.276 × 10^–01^	67	11	1.989 × 10^–3^	1.400 × 10^–1^	3.695 × 10^–3^	3.453 × 10^–2^
EGFR tyrosine kinase inhibitor resistance	hsa01521	79	10	1.344 × 10^–2^	0	0	1	79	10	1.930 × 10^–2^	1.400 × 10^–2^	3.605 × 10^–3^	3.453 × 10^–2^
AMPK signaling pathway	hsa04152	119	13	1.720 × 10^–2^	4	1	8.134 × 10^–01^	123	14	1.551 × 10^–2^	2.100 × 10^–2^	4.196 × 10^–3^	3.668 × 10^–2^
amoebiasis	hsa05146	100	16	1.500 × 10^–4^	2	0	1	102	16	3.612 × 10^–4^	8.890 × 10^–1^	4.148 × 10^–3^	3.668 × 10^–2^
small-cell lung cancer^a^	hsa05222	92	13	1.984 × 10^–3^	0	0	1	92	13	3.255 × 10^–3^	1.050 × 10^–1^	4.364 × 10^–3^	3.696 × 10^–2^
glycerophospholipid metabolism	hsa00564	97	12	8.567 × 10^–3^	8	3	5.514 × 10^–01^	105	15	1.465 × 10^–3^	2.550 × 10^–1^	4.690 × 10^–3^	3.747 × 10^–2^
axon guidance	hsa04360	181	21	1.430 × 10^–3^	0	0	1	181	21	2.838 × 10^–3^	1.320 × 10^–1^	4.701 × 10^–3^	3.747 × 10^–2^

a MYC proto-oncogene involved; ID: pathway ID of KEGG; G_n: the number of genes in a pathway; deG_n: the number of differentially expressed genes in a pathway; G_pORA: *p*-value of the gene’s ORA of a pathway; C_n: the number of compounds in a pathway; deC_n: the number of significant metabolites in a pathway; C_pORA: *p*-value of the metabolite’s ORA of a pathway; P_n: G_n + C_n; deP_n: deG_n + deC_n.

b Each row represents a pathway, where the first column denotes the pathway’s name, and the ID corresponds to the pathway ID in the KEGG database. If only genes are considered, then the information includes the total number of genes in the pathway (G_n), the number of significant genes (deG_n), and the *p*-value of the ORA test (G_pORA). If only compounds are considered, then the details consist of the total number of compounds in the pathway (C_n), the significant number of compounds (deC_n), and the *p*-value of the ORA test (C_pORA). If both genes and compounds are taken into account, then the data include the total number of genes and compounds in the pathway (P_n), the number of significant genes and compounds (deP_n), and the *p*-value of the ORA test (p_pORA). The next includes results from the eSPIA test (peSPIA) considering pathway topology. Additionally, it integrates ORA and eSPIA tests using the Pbine method, listing raw and FDR-corrected *p*-values (pPbine and pPbine_FDR), with data ranked by pPbine_FDR.

### Postanalysis

#### Peak Identification

We utilized the narcotics data set to demonstrate the functionality of the peak identification module. The estimated *m*/*z* values for the 12 narcotics were employed in a targeted analysis for peak identification. Among these, heroin and nimetazepam could not be found in the HMDB database. However, of the remaining 10 narcotics, seven were successfully identified in HMDB using a mass tolerance setting of 2 ppm, and an additional three narcotics were retrieved when the tolerance discerned was expanded to 3 ppm.

In the MassBank database, only nimetazepam was absent from the list of the 12 narcotics. Among the other 11 narcotics, eight were successfully matched with a 2 ppm mass tolerance, and three more became identifiable at 3 ppm. In total, 11 out of 12 narcotics were accurately identified using HMDB or MassBank.

Generative AI chatbots for information retrieval have recently gained popularity.^80–82^ In our study, we employed several widely used large language model (LLM) chatbots, including OpenAI’s ChatGPT-4 and ChatGPT-3.5, Meta’s Llama3-70B, and Windows Copilot (Precision Mode), for *m*/*z* comparisons and compound identification. Using well-designed prompts (see the Supporting Information “Peak identification” (Text S3)), ChatGPT-4, ChatGPT-3.5, and Llama3-70B correctly identified all 12 narcotics. In contrast, Windows Copilot failed to identify delta9-THC and nimetazepam and incorrectly identified FM2 by suggesting alternative compounds. The detailed results of the peak identification analysis are summarized (Table 3).

**Table 3:** Peak Identification of the 12 Narcotics^b^

							SMART 2.0		AI chatbot			
peak index	name	estimated *m*/*z*	adduct *m*/*z* (M + H)	delta (ppm)^a^	formula	HMDB ID	HMDB	MassBank	ChatGPT-4	ChatGPT-3.5	Llama3	Copilot
1	heroin (diacetylmorphine)	370.1642	370.1649	1.8910	C_21_H_23_NO_5_	NA	NA	Y	Y	Y	Y	Y
2	morphine	286.1430	286.1438	2.7958	C_17_H_19_NO_3_	HMDB0014440	Y	Y	Y	Y	Y	Y
3	cocaine	304.1538	304.1543	1.6439	C_17_H_21_NO_4_	HMDB0015043	Y	Y	Y	Y	Y	Y
4	thebaine	312.1586	312.1594	2.5628	C_19_H_21_NO_3_	HMDB0029378	Y	Y	Y	Y	Y	Y
5	delta9-THC (delta9-tetrahydrocannabinol)	315.2318	315.2319	0.3172	C_21_H_30_O_2_	HMDB0014613	Y	Y	Y	Y	Y	N
6	amphetamine	136.1118	136.1121	2.2041	C_9_H_13_N	HMDB0014328	Y	Y	Y	Y	Y	Y
7	MA (methamphetamine)	150.1273	150.1277	2.6644	C_10_H_15_N	HMDB0015517	Y	Y	Y	Y	Y	Y
8	MDMA (3,4-methylenedioxymethamphetamine)	194.1172	194.1176	2.0606	C_11_H_15_NO_2_	HMDB0254382	Y	Y	Y	Y	Y	Y
9	MDA (love drug; 3,4-methylenedioxyamphetamine)	180.1017	180.1019	1.1105	C_10_H_13_NO_2_	HMDB0041931	Y	Y	Y	Y	Y	Y
10	ketamine	238.0992	238.0993	0.4200	C_13_H_16_ClNO	HMDB0015352	Y	Y	Y	Y	Y	Y
11	FM2 (flunitrazepam)	314.0930	314.0935	1.5919	C_16_H_12_FN_3_O_3_	HMDB0015510	Y	Y	Y	Y	Y	W
12	nimetazepam	296.1025	296.1030	1.6886	C_16_H_13_N_3_O_3_	NA	NA	NA	Y	Y	Y	N

a Delta = abs­(estimated *m*/*z* – adduct *m*/*z*)/adduct *m*/*z*.

b The peak analysis module determines the “estimated *m*/*z*” values. Twelve narcotics can be identified within a tolerance of 3 ppm using SMART 2.0, provided that they are included in the database and AI chatbots. “NA” denotes substances not present in the HMDB or MassBank databases. “Y” signifies a correct identification, “N” indicates a failure to identify any compound, and “W” signifies a wrong ID.

#### Concentration Calibration

The concentration calibration module provides two primary functions: (1) calibration curve construction and (2) concentration calculation. We employed the narcotics data set to illustrate the functionality of the concentration calibration module.

##### Calibration Curve Construction

We summarized the highest adjusted *R* ^2^ values obtained by constructing calibration curves, employing linear and quadratic models, across 10 concentrations for 12 narcotics (Table 4). For instance, heroin was subjected to the linear model with a weight set to 1/*x*, and outlier detection employed the bias method. The available samples included concentrations of 100, 200, 300, 700, and 800, yielding the highest adjusted *R* ^2^ of 0.9873. When employing the quadratic model, the weight was set to 1/*x* ^2^, and outlier detection utilized a 95% CI. The available samples included concentrations of 50, 100, 200, 300, 700, 800, 900, and 1000, resulting in the highest adjusted *R* ^2^ of 0.9983. Figure 6 utilizes heroin as an example to illustrate the calibration curve constructed under the various scenarios mentioned above. The results for the other 11 narcotics are presented in Figure S5A–K.

**Figure 6:**
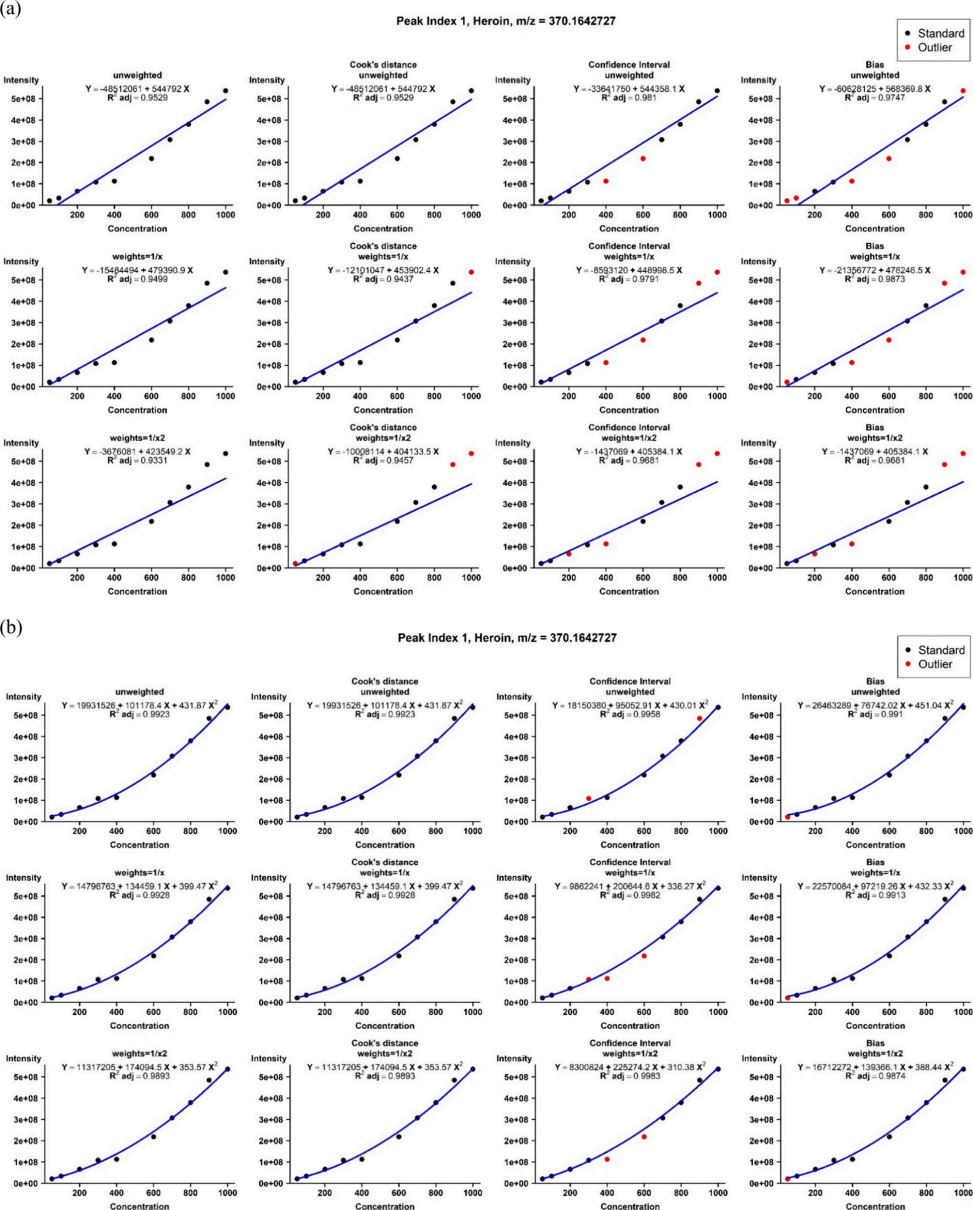
Calibration curve of heroin. It was constructed using either a linear or quadratic model, with various weightings, including unweighted, 1/*x*, and 1/*x* ^2^. Additionally, various outlier detection methods were employed, including Cook’s *D* (>4/*n*, where *n* represents the sample size), a 95% confidence interval (CI), and the bias method (with a 20% relative error). The figure displays the calibration curve equations under various scenarios, along with adjusted *R* ^2^ values. Red denotes outliers, while black indicates the retained standards. (a) Linear model. (b) Quadratic model.

**Table 4:** Calibration Curves of the 12 Narcotics: Results of Constructing the Linear and Quadratic Calibration Curves with the Highest Adjusted *R* ^2^ Value (adj. *R* ^2^) for 10 Concentrations of 12 Narcotics

	linear model						quadratic model				
name	weight	outlier detection	sample^a^	***R*** ^2^	adj. *R* ^2^		weight	outlier detection	sample^a^	***R*** ^2^	**adj. *R*** ^2^
heroin	1/*x*	bias	100; 200; 300; 700; 800	0.9904	0.9873		1/*x* ^2^	CI	50; 100; 200; 300; 700; 800; 900; 1000	0.9988	0.9983
morphine	unweighted	CI	50; 100; 200; 300; 700; 800; 900; 1000	0.9958	0.9951		1/*x*	CI	50; 100; 200; 300; 600; 700; 800; 900; 1000	0.9983	0.9978
cocaine	unweighted	CI	50; 100; 200; 300; 700; 800; 900; 1000	0.9926	0.9914		1/*x*	CI	50; 100; 200; 300; 600; 700; 800; 900; 1000	0.9988	0.9984
thebaine	1/*x*	bias	100; 700; 800; 900	0.9962	0.9943		1/*x*	CI	50; 100; 200; 300; 600; 700; 900; 1000	0.9989	0.9985
delta9-THC^b^	1/*x* ^2^	CI	50; 600; 700; 800; 900	0.9995	0.9994		1/*x*	CI	50; 100; 200; 300; 600; 700; 800; 900; 1000	0.9895	0.9861
amphetamine	1/*x*	CI	50; 100; 600; 700; 800; 900; 1000	0.8863	0.8636		1/*x* ^2^	CI	50; 100; 300; 400; 600; 800; 900; 1000	0.9902	0.9863
MA^b^	1/*x* ^2^	CI	50; 100; 400; 600; 700; 800	0.9977	0.9971		unweighted	CI	50; 100; 200; 300; 600; 800; 900; 1000	0.9990	0.9987
MDMA^b^	1/*x*	bias	100; 300; 600; 700; 800; 900	0.9909	0.9887		unweighted	CI	50; 100; 200; 600; 700; 800; 900; 1000	0.9995	0.9993
MDA^b^	1/*x* ^2^	bias	50; 600; 700; 800	0.9929	0.9894		unweighted	CI	50; 100; 200; 400; 600; 700; 900; 1000	0.9990	0.9987
ketamine	1/*x*	bias	100; 200; 300; 600; 700; 800; 900	0.9981	0.9978		unweighted	bias	100; 200; 300; 600; 700; 800; 900; 1000	0.9995	0.9993
FM2^b^	unweighted	CI	50; 100; 200; 300; 700; 800; 900	0.9949	0.9939		1/*x*	CI	50; 100; 600; 700; 800; 900; 1000	0.9998	0.9997
nimetazepam	1/*x* ^2^	CI	50; 200; 300; 600; 700; 800	0.9949	0.9936		unweighted	CI	50; 100; 200; 600; 700; 800; 900; 1000	0.9990	0.9985

a The calibration curve samples were chosen after removing outliers.

b delta9-THC: delta9-tetrahydrocannabinol; MA: methamphetamine; MDMA: 3,4-methylenedioxymethamphetamine; MDA: 3,4-methylenedioxyamphetamine; FM2: flunitrazepam.

##### Concentration Calculation

We determined the concentrations of the 12 narcotics in the 500 ppb sample and presented the relative error for each narcotic (Table 5). The concentration of morphine was estimated with the lowest relative error (−0.0891%), and the concentration of amphetamine was estimated with the highest relative error (54.7643%). Furthermore, Figure 7 illustrates the relationship between the optimal linear calibration curve and the calculated concentration of heroin in the 500 ppb test sample. The results for the other 11 narcotics are presented in Figure S6A–K.

**Figure 7:**
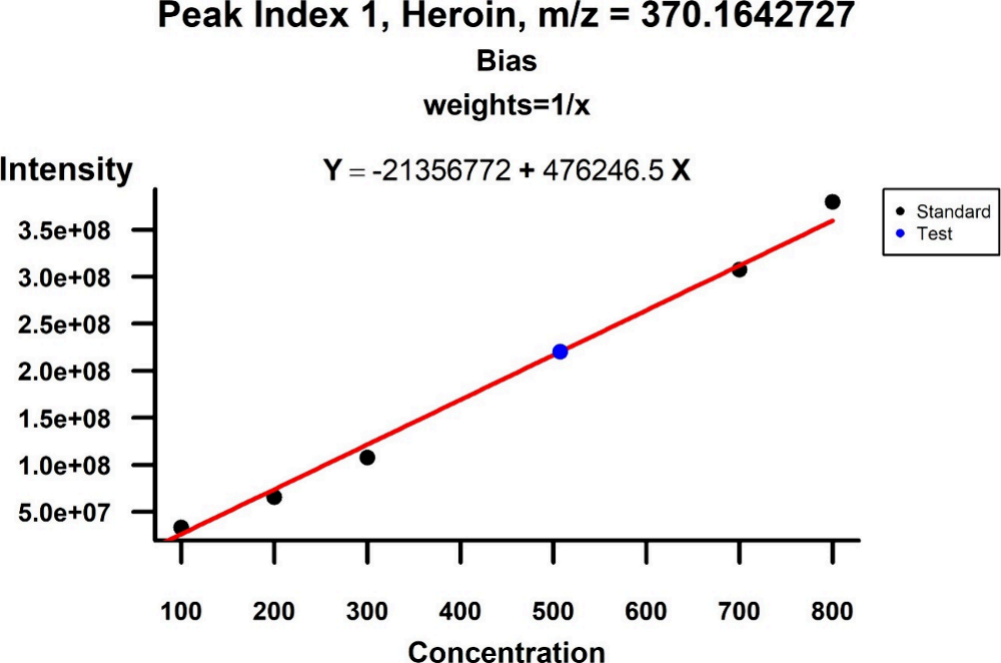
Optimal calibrated concentration of heroin. The graph illustrates the calculated concentrations of heroin in the 500 ppb test sample, utilizing the optimal linear calibration curve with the weighted method as 1/*x* and applying the Bias method to detect outliers. The blue dot denotes the 500 ppb test sample, while black dots indicate the other standards.

**Table 5:** Determined Concentration and Relative Error of the 12 Narcotics in the 500 ppb Test Sample^b^

peak index	name	*m*/*z*	RT (s)	T1_500_MS1^a^ (ppb)	relative error (%)
1	heroin	370.1643	685.3724	507.4039	1.4808
2	morphine	286.1431	146.2696	499.5544	–0.0891
3	cocaine	304.1538	701.1360	501.2351	0.2470
4	thebaine	312.1588	678.8176	450.4228	–9.9154
5	delta9-THC (delta9-tetrahydrocannabinol)	315.2302	1513.3190	467.1095	–6.5781
6	amphetamine	136.0804	419.1944	773.8217	54.7643
7	MA (methamphetamine)	150.1021	496.8365	595.2049	19.0410
8	MDMA (3,4-methylenedioxymethamphetamine)	194.1173	538.9991	520.7164	4.1433
9	MDA (love drug; 3,4-methylenedioxyamphetamine)	180.0866	505.6407	492.0814	–1.5837
10	ketamine	238.0988	599.9313	547.7417	9.5483
11	FM2 (flunitrazepam)	314.0930	1044.2740	512.0763	2.4153
12	nimetazepam	296.1024	1040.5220	537.2955	7.4591

a Estimated concentration. T1_500_MS1 is the name of the test sample, denoting sample T1 with MS1 data and a concentration of 500 ppb.

b We utilized the widely adopted linear model to construct the calibration curve, incorporating weights such as unweighted, 1/*x*, and 1/*x* ^2^. Outlier identification methods, including Cook’s *D*, a 95% CI, and the bias method (with a 20% relative error), were applied to detect and eliminate outliers.

## Discussion

We discuss the new features and capabilities of SMART 2.0, which aim to enhance accessibility, interoperability, and analytical depth in metabolomics research.

### Targeted Peak Analysis

In the case of targeted peak analysis, it is essential to provide *m*/*z* information. Providing RT information is highly beneficial, especially since the *m*/*z* values of many compounds can be very similar or even identical. If RT information is not provided, then SMART 2.0 can automatically identify the peak corresponding to the maximum intensity of the specified *m*/*z* value and assign the correct RT to that peak. However, it is important to note that it may not be accurately detected in cases where the peak is of low abundance. From the demonstration of the narcotics data set, the S/N ratio of amphetamine was found to be only 0.19. This low ratio suggests that further investigation is required to assess whether inappropriate peak detection parameters may have led to the failure to capture the correct signal. As shown in Figure S2A, the MS1 spectra of amphetamine exhibit multiple peaks, implying that the originally defined RT window may have been too narrow to encompass all relevant signals. Additionally, the presence of multiple MS1 peaks may be attributed to the formation of protomers—structural isomers generated by protonation at different sites under electrospray ionization (ESI) conditions—particularly in basic compounds like amphetamine and especially at higher concentrations.^83,84^ This interpretation is supported by MS/MS analysis at 500 ppb, which revealed that both MS1 peaks produced identical fragmentation patterns (*m*/*z* 91, 119, and 136) (Figure S2B), consistent with authentic amphetamine, confirming that these peaks originate from the same compound.

### Data Preprocessing

SMART 2.0 introduces additional data preprocessing options not available in SMART 1.0, including PS^41^ and RINT,^51^ to enhance the flexibility and robustness of metabolomics analysis workflows. PS is widely used to stabilize variance across features and improve pattern recognition in multivariate analyses,^85^ while RINT helps normalize skewed data distributions, facilitating the application of statistical methods that assume normality. By incorporating both scaling and transformation tools, SMART 2.0 empowers users to better accommodate diverse data characteristics and optimize model performance in downstream analyses.

### PLS/PLS-DA

In SMART 1.0, the statistical analysis primarily utilized the (untargeted) metabolome-wide association study using ANCOVA, which focused on identifying individual significantly trait-associated metabolites. The addition of PLS/PLS-DA module enables the integration of information from multiple genes and metabolites, thereby enhancing the evidential strength of the statistical methods. From the analysis of the breast cancer data set, we found that using only gene expression data for PLS-DA analysis resulted in a *Q2Y* value of 0.539 (Figure S3A). Using only metabolite data, the *Q2Y* value was 0.454 (Figure S3B). When genes and metabolites were combined in the analysis, the *Q2Y* value increased to 0.55 (Figure S3C). In the PLS-DA model, we use VIP values to pinpoint genes and metabolites with significant contributions, and higher VIP values indicate that these markers play a crucial role in the PLS model’s predictive accuracy and biological interpretation (Table S2). The top three VIP genes (*DENND2A*, *VIT*, and *SEMA3G*) and metabolites (*C*-glycosyltryptophan, dimethylarginine, and phosphoethanolamine) have been documented to be related to breast cancer.^86–91^ Further biological experiments are warranted to confirm the significance of other novel genes and metabolites that have not yet been documented.

### IOPA

In the IOPA module, we integrate various omics data and multiple markers through pathways. Based on the breast cancer data set, two markers of interest from previous studies,^78,92–94^ MYC proto-oncogene (gene ID 4609) and the oncometabolite 2-hydroxyglutarate (2HG) (KEGG ID C02630), were found to be significant in a single-point analysis using ANCOVA. Additionally, the MYC proto-oncogene was involved in 8 out of the 34 identified pathways (Table 2), among which the PI3K-Akt signaling pathway (hsa04151) stood out as the most significant, with an FDR-corrected *p*-value of 2.80 × 10^–10^ using the Pbine method. The PI3K-Akt signaling pathway plays a crucial role in breast cancer development and progression. Activation of this pathway is frequently observed in breast cancer cells, contributing to increased cell proliferation, survival, and metastasis.^95–97^ Inhibition of PI3K-Akt signaling has shown promise in suppressing breast cancer growth both in vitro and in vivo.^98,99^ Moreover, targeting specific components of this pathway has emerged as a potential therapeutic strategy for breast cancer treatment.^100,101^

Although the significance of the PI3K-Akt signaling pathway primarily arises from its influence on gene expression, integrating genes and metabolites through IOPA reveals additional pathways linked to metabolites. Notably, examples such as the pentose phosphate pathway (hsa00030) and fructose and mannose metabolism (hsa00051) emerge. In particular, the gene and metabolite ORA tests for the pentose phosphate pathway exhibit significance, with *p*-values of 0.02202 and 0.02009, respectively. However, the integrated ORA test demonstrates even greater significance, yielding a *p*-value of 6.891 × 10^–6^. For fructose and mannose metabolism, the ORA test of genes alone yields nonsignificance (*p* = 0.11411), whereas the ORA test of metabolites alone demonstrates significance (*p* = 0.00693). However, upon integrating genes and metabolites, the significance is further amplified (*p* = 9.345 × 10^–05^). This underscores the enhanced understanding achieved by integrating transcriptomic and metabolomics data in pathway analysis. The enrichment of the pentose phosphate pathway (hsa00030) and fructose and mannose metabolism (hsa00051) aligns with their known metabolic roles in cancer. The pentose phosphate pathway supports tumor proliferation and redox homeostasis through dual high-flux branches.^102^ Additionally, fructose metabolism promotes breast cancer metastasis via nuclear kinase activity of ketohexokinase-A, which mediates fructose-induced signaling.^103^

Considering both ORA and topology-based analyses, neither ORA nor eSPIA alone reached statistical significance for the EGFR tyrosine kinase inhibitor resistance pathway (hsa01521) (*p* = 0.019 and 0.014; pFDR = 0.076 and 0.361, respectively) or the AMPK signaling pathway (hsa04152) (*p* = 0.016 and 0.021; pFDR = 0.068 and 0.407, respectively). Notably, combining the *p*-values using the Pbine method improved the statistical significance of both pathways (*p* = 0.004 and 0.004; pFDR = 0.035 and 0.037, respectively), suggesting that integrating ORA and eSPIA enhances detection sensitivity. Importantly, this increased significance aligns with known cancer biology. The EGFR signaling pathway is frequently dysregulated in early stage breast cancer and is associated with altered circulating EGFR levels, supporting its utility as both a circulating biomarker and therapeutic target.^104^ Similarly, the AMPK signaling pathway acts as a central regulator of cellular metabolism and immune modulation within the tumor microenvironment, positioning it as a promising target for cancer therapy.^84^ Together, EGFR and AMPK pathways represent biological meaningful targets in breast cancer pathophysiology and are actively pursued in precision oncology.

### Peak Identification

The first postanalysis module, peak identification, utilizes *m*/*z* data from HMDB and MassBank for matching purposes, allowing for rapid comparison of unknown peaks. When the candidate narcotics are predetermined, all can be identified within a tolerance of 3 ppm using SMART 2.0 (provided that they are included in the database) and generative AI chatbots (excluding Copilot). Nevertheless, in the absence of precise drug information, direct one-to-one comparison of these narcotics is not feasible due to the potential presence of other metabolites with identical *m*/*z* values, even with the assistance of AI chatbots. Consequently, additional experiments such as MS/MS analysis, standard reference comparison, nuclear magnetic resonance (NMR) spectroscopy, and chemical reaction validation are essential to ascertain the identities of unknown peaks. Although HMDB does not encompass heroin and nimetazepam and even SMART 2.0 is unable to detect them, AI chatbots can expeditiously and accurately integrate online data to identify the correct results. Generative AI has become widely utilized in medical research, aiding clinicians and researchers in making reliable medical decisions.^105–107^ However, it is crucial to consider the nuances, applicability, and consistency of their responses and to establish the most effective query protocols to ensure accurate results.^108–110^ Our plans include integrating AI methodologies into SMART as an auxiliary tool to enhance its capabilities.

### Concentration Calibration

The second module, concentration calibration, includes two main components: establishing a calibration curve using standards and utilizing this curve to estimate the concentrations of other samples. In the narcotics analysis, peak analysis revealed that the amphetamine signal was excessively broad and consisted of multiple peaks (Figure S2A). As a result, calibration curve construction using either linear or quadratic models produced suboptimal fits (Figure S5E), likely due to signal variability. This suggests the need to re-evaluate the LC-MS experimental conditions for amphetamine to enhance quantification accuracy and data quality. Moreover, the presence of multiple MS1 peaks may be caused by the formation of protomers under ESI conditions, which can introduce variability in peak shape and intensity, especially at higher concentrations. The simplest effective model should be used to characterize the concentration–response relationship.^111^ Additionally, the selection of weighting and regression equations must be justified by the data’s characteristics and research objectives.^112,113^

The second component involves using the calibration curve, established with standards under consistent experimental conditions, to estimate unknown sample concentrations. In the narcotics analysis, estimated concentrations and relative errors for all drugs, except amphetamine and methamphetamine, were within a 10% range. The discrepancy for amphetamine is due to inaccurate peak abundance, and for methamphetamine, a higher calibration concentration of ∼600 ppb was observed (Figure S6F), suggesting variations in the LC-MS experiment. Increasing the number of replicates in future experiments is recommended to enhance accuracy.

Consistency in LC-MS experimental conditions is crucial for constructing a reliable calibration curve and achieving accurate quantification. Conducting multiple replicates at identical concentrations improves calibration curve stability, thereby enhancing the accuracy of unknown sample concentration estimates.^112^

## Conclusions

SMART 2.0 offers an end-to-end platform for modern metabolomics analysis. Building on version 1.0, SMART 2.0 introduces an enhanced suite of features, including targeted peak detection, accurate quantification of peak abundance, robust data analytics, and novel multiomics pathway analysis. It also supports the rapid identification of unknown metabolites and the construction of calibration curves for precise concentration quantification, improving reliability and interpretability.

Remarkably, to demonstrate the practical applications and boundaries of targeted data analysis using SMART 2.0, we conducted a new MS and MS/MS experiment focusing on 12 narcotic compounds. The data set and analysis provide valuable insights into the metabolomics profiles, fragmentation patterns, and quantification tolerances of these 12 narcotics. The findings contribute to the development of more reliable approaches for detecting and monitoring drug use in various forensic and biomedical contexts.

By combining usability with analytical depth, SMART 2.0 complements existing tools and fills critical gaps in the metabolomics workflow. Looking ahead, we aim to expand further SMART’s capabilities to support integrative analyses across multiomics and multimodality data. With advances in artificial intelligence and data science, SMART will empower researchers to explore complex biological systems and generate actionable insights in fields such as disease diagnostics, precision medicine, drug discovery, nutrition, and environmental health.

## Supplementary Material



## Notes

(Text S1) Peak analysis; (Text S2) integrative omics pathway analysis; (Text S3) post analysis (peak identification); (Text S4) post analysis (concentration calibration); (Text S5) drug experiment; (Table S1) performance of PLS-DA for genes and metabolites using different selection criteria; (Table S2) list of significant genes and metabolites with top 10 VIP scores; (Figure S1) comparison of SMART 2.0 and 1.0; (Figure S2) MS1 and MS2 spectra of amphetamine, cocaine, delta9-THC, FM2, heroin, ketamine, MDA (love drug), MA, MDMA, morphine, nimetazepam, and thebaine, all at a concentration of 500 ppb; (Figure S3) score plots from PLS-DA of gene expression data, metabolomics data, and integrated gene expression and metabolomics data; (Figure S4) volcano plot from the IOPA analysis of breast cancer data; (Figure S5) calibration curves for morphine, cocaine, thebaine, delta9-THC, amphetamine, MA, MDMA, MDA (love drug), ketamine, FM2, and nimetazepam; (Figure S6) optimal calibrated concentration plots for morphine, cocaine, thebaine, delta9-THC, amphetamine, MA, MDMA, MDA (love drug), ketamine, FM2, and nimetazepam (PDF)

## Abbreviations

ESI: electrospray ionization
GSA: gene set analysis
GSEA: gene set enrichment analysis
HMDB: Human Metabolome Database
IOPA: integrative omics pathway analysis
MWASs: metabolome-wide association studies
ORA: over-representation analysis
PLS/PLS-DA: partial least-squares and partial least-squares discriminant analysis
PS: Pareto scaling
RINT: rank-based inverse normal transformation
RMSEE: root-mean-square error of estimation
S/N: signal-to-noise ratio
SPIA: signaling pathway impact analysis
