# Supplementary material for: SMART 2.0 Statistical Metabolomics Analysis: An R Tool 2.0
Source: Anal Chem. 2025 Oct 31;97(46):25453–68. doi: 10.1021/acs.analchem.5c03225 (PMC12658861; doi:10.1021/acs.analchem.5c03225)
Supplement: Supplementary file 1 [file ac5c03225_si_001.pdf]

Supporting Information

SMART 2.0: Statistical Metabolomics Analysis: An R Tool 2.0

Yu-Jen Liang[1], Chih-Ting Yang[1], Chia-Wei Chen[1], Yin-Chun Lin[1], Shu-Yao Lin[2,3], Yi Sheng Wang[4,*] and Hsin-Chou Yang[1,5,6,7,*]

[1] Institute of Statistical Science, Academia Sinica, Taipei 115, Taiwan

[2] Agricultural Biotechnology Research Center, Academia Sinica, Taipei 115, Taiwan

[3] Department of Chemistry, National Sun Yat-sen University

[4] Genomics Research Center, Academia Sinica, Taipei 115, Taiwan

[5] Biomedical Translation Research Center, Academia Sinica, Taipei 115, Taiwan

[6] Department of Statistics, National Cheng Kung University, Tainan 701, Taiwan

[7] Institute of Public Health, National Yang Ming Chiao Tung University, Taipei 11221, Taiwan

* To whom correspondence should be addressed.

Name: [Hsin-Chou Yang]; Tel: [886-2-27875686]; Fax: [886-2-27886833]; Email: [hsinchou@stat.sinica.edu.tw]; Present Address: Office No. 4016, Environmental Changes Research Building A, Institute of Statistical Science, Academia Sinica. No. 128, Sec. 2, Academia Road, Nankang, Taipei, Taiwan 115

Name: [Yi Sheng Wang]; Tel: [886-2-27871272]; Email: [wer@gate.sinica.edu.tw]; Present Address: Genomics Research Center, Academia Sinica. No. 128, Sec. 2, Academia Road, Nankang, Taipei, Taiwan 115

Table of Contents

TEXTS

Text S1. Peak analysis S-4

Text S2. Integrative omics pathway analysis S-5

Text S3. Post analysis (peak identification) S-8

Text S4. Post analysis (concentration calibration) S-10

Text S5. Drug experiment S-11

TABLES

Table S1. Performance of PLS-DA for genes and metabolites using different selection criteria S-12

Table S2. List of significant genes and metabolites with top 10 VIP scores S-13

FIGURES

Figure S1. Comparison of SMART 2.0 and 1.0 .... S-14

Figure S2A. MS1 and MS2 spectra of amphetamine at a concentration of 500 ppb S-15

Figure S2B. MS1 and MS2 spectra of cocaine at a concentration of 500 ppb S-16

Figure S2C. MS1 and MS2 spectra of delta9-THC at a concentration of 500 ppb S-17

Figure S2D. MS1 and MS2 spectra of FM2 at a concentration of 500 ppb S-18

Figure S2E. MS1 and MS2 spectra of heroin at a concentration of 500 ppb S-19

Figure S2F. MS1 and MS2 spectra of ketamine at a concentration of 500 ppb S-20

Figure S2G. MS1 and MS2 spectra of MDA (love drug) at a concentration of 500 ppb S-21

Figure S2H. MS1 and MS2 spectra of MA at a concentration of 500 ppb S-22

Figure S2I. MS1 and MS2 spectra of MDMA at a concentration of 500 ppb S-23

Figure S2J. MS1 and MS2 spectra of morphine at a concentration of 500 ppb S-24

Figure S2K. MS1 and MS2 spectra of nimetazepam at a concentration of 500 ppb S-25

Figure S2L. MS1 and MS2 spectra of thebaine at a concentration of 500 ppb S-26

Figure S3A. Score plot from PLS-DA of gene expression data S-27

Figure S3B. Score plot from PLS-DA of metabolomics data S-28

Figure S3C. Score plot from PLS-DA on integrated gene expression and metabolomics data S-29

Figure S4. Volcano plot from the IOPA analysis of breast cancer data S-30

Figure S5A. Calibration curve for morphine S-31

Figure S5B. Calibration curve for cocaine S-33

Figure S5C. Calibration curve for thebaine S-35

Figure S5D. Calibration curve for delta9-THC S-37

Figure S5E. Calibration curve for amphetamine S-39

Figure S5F. Calibration curve for MA ……………………………………………………………S-41

Figure S5G. Calibration curve for MDMA ………………………………………………………........ S-43

Figure S5H. Calibration curve for MDA (love drug) ……………………………………………......... S-45

Figure S5I. Calibration curve for ketamine ………………………………………………………........ S-47

Figure S5J. Calibration curve for FM2 ……………………………………………………………S-49

Figure S5K. The calibration curve for nimetazepam ……………………………………….………...S-51

Figure S6A. Optimal calibrated concentration plot for morphine ……………………………….………S-53

Figure S6B. Optimal calibrated concentration plot for cocaine …………………………….……….S-54

Figure S6C. Optimal calibrated concentration plot for thebaine …………………………………………S-55

Figure S6D. Optimal calibrated concentration plot for delta9-THC ……………………………..…S-56

Figure S6E. Optimal calibrated concentration plot for amphetamine ……………………………………S-57

Figure S6F. Optimal calibrated concentration plot for MA ……………………………………………S-58

Figure S6G. Optimal calibrated concentration plot for MDMA …………………………………………S-59

Figure S6H. Optimal calibrated concentration plot for MDA (love drug) ………………………………S-60

Figure S6I. Optimal calibrated concentration plot for ketamine …………………………………………S-61

Figure S6J. Optimal calibrated concentration plot for FM2 ……………………………….………S-62

Figure S6K. Optimal calibrated concentration plot for nimetazepam ………………………..……..…S-63

REFERENCES. Literature cited in this Supporting Information ………………………..…..………S-64

SUPPLEMENTAL TEXTS

Text S1. Peak analysis

1. Peak detection

   To perform peak detection, mzXML files containing MS1, or MS2, or both MS1 and MS2 data were required. The xcms package [1] in R, specifically the function xcmsRaw, was utilized to read the mzXML files. This step enabled the generation of peak profiles for each sample, serving as an initial phase for peak detection.

2. Peak abundance calculation

   For peak abundance calculation, the true m/z information for both MS1 and MS2 was provided. While RT information was optional, tolerance values for m/z and RT were set. The RT tolerance could be specified as a constant, provided by the user, or automatically detected by SMART.

   If the user provided RT information, the process began with the scanning of MS1 data. The m/z and RT values were locked within the specified tolerance ranges, and the maximum intensity within these ranges was identified. Abundance of MS1 was determined by accumulating the intensity within the tolerance range. The process was repeated for MS2 data, using the m/z and RT information for each fragment as provided by the user to lock onto the targets. The maximum intensity within the tolerance range was identified for each fragment, and the intensities within their respective tolerance ranges were accumulated to calculate the peak abundance of MS2.

   If the user did not provide RT information, the analysis started with MS1 data. Initially, m/z was locked within the tolerance range, and the maximum intensity was identified. At this point, the RT value corresponding to the maximum intensity was set as the RT for this peak. Peak abundance of MS1 was determined by accumulating the intensity within the tolerance range. Subsequently, MS2 data was scanned. The process for MS2 mirrored that of MS1, using the m/z and RT information obtained during MS1 to lock onto the target peaks, finding the maximum intensity within the tolerance range, and then accumulating the intensities within these tolerance ranges to calculate the peak abundance of MS2.

Text S2. Integrative omics pathway analysis

For a given KEGG pathway, we computed the cumulative perturbation within the pathway using the concept of SPIA [2]. We extended this approach from signal pathways to include metabolism pathways, creating the method named eSPIA.

We obtained information on 347 KEGG pathways using the R package KEGGREST [3]. All pathway structures were parsed with the R package KEGGgraph [4]. Additionally, we extracted topology information for 304 pathways using eSPIA. eSPIA is based on the R package SPIA, which we extended to derive the normalized weighted directed adjacency matrix, denoted as B, representing the relationships between genes and compounds.

Here is an example of a normalized weighted directed adjacency matrix B:

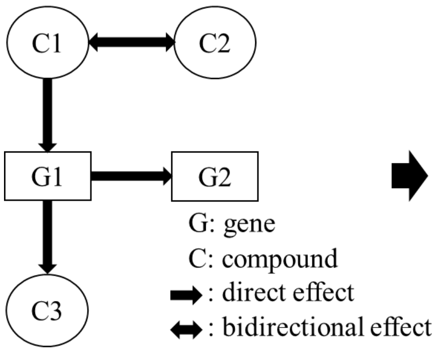

$$B = \begin{array}{c} \begin{matrix} C1 & C2 & C3 & G1 & G2 \end{matrix} \\ \begin{bmatrix} 0 & 1 & 0 & 0 & 0 \\ .5 & 0 & 0 & 0 & 0 \\ 0 & 0 & 0 & .5 & 0 \\ .5 & 0 & 0 & 0 & 0 \\ 0 & 0 & 0 & .5 & 0 \end{bmatrix} \end{array} \begin{matrix} C1 \\ C2 \\ C3 \\ G1 \\ G2 \end{matrix}$$

In this pathway, we have 5 nodes represents three compounds and two genes, so B is a 5 times 5 matrix where each entry b_{ij} represents the normalized weight of the directed edge from node i to node j in the pathway. The values are normalized to reflect the relationships between genes and/or compounds within the pathway.

In total, we analyzed 304 non-zero matrices B in our pathway analysis. Combining these with input between-group effect size ($\triangle E$), we calculated the accumulations of perturbations (Acc) using eSPIA for each pathway to assess the pathway's impact.

In order to ensure comparability of eSPIA statistics, we performed normalization. We developed a normalized statistic (normalized eSPIA) to evaluate candidate pathways. The statistic of accumulated perturbation (noted as t_A) was defined as the sum of the net perturbation accumulation, given by $\sum_i Acc(g_i)$, where,

$Acc(g_i) = PF(g_i) - \triangle E(g_i)$,

$PF(g_i) = \triangle E(g_i) + \sum_{j=1}^{n} \beta_{ij} \frac{PF(g_j)}{N_{ds}(g_j)}$,

g_i: The gene or compound i in the pathway.

$\triangle E(g_i)$: Between-group effect size of g_i.

$PF(g_j)$: Perturbation factors of g_j directly upstream of the target g_i.

$N_{ds}(g_j)$: The number of downstream genes or compounds of each g_i.

β_{ij}: The strength of the interaction between g_j and g_i.

We employed a bootstrap approach that randomized the shuffle of g_i to test the significance of statistic t_A for each pathway. The corresponding p-value was denoted as $P_{PERT} = P(T_A \geq t_A | H_0)$.

Since the size of t_A is influenced by g_i, the expression size, number of nodes (genes or compounds) in the pathway, and the pathway's topology, we proposed a normalized statistic t_{AN}, defined as:

$t_{AN} = \frac{t_A}{\|B(I-B)^{-1}\|_1 \|\triangle E\|_1}$,

where B is a matrix with entries $b_{ij} = \frac{\beta_{ij}}{N_{ds}(g_j)}$, $\triangle E$ is a vector with entries $\triangle E(g_i)$.

The normalized statistic t_{AN} ranged from -1 to 1 (see appendix), where more extreme values indicate a greater impact on the pathway. Therefore, t_{AN} represents the normalized eSPIA scores.

In addition, we calculated the p-value from over-representation analysis (ORA) and combined these two pieces of evidence using the Pbine [5] package to obtain the overall significant value for each pathway.

Appendix

Proof of the range of t_{AN}.

Matrix form of $Acc(g_i) = PF(g_i) - \triangle E(g_i) = \sum_{j=1}^{n} \beta_{ij} \frac{PF(g_j)}{N_{ds}(g_j)}$

is: $Acc = B(PF) = B(Acc + \triangle E)$, where $B_{ij} = \frac{\beta_{ij}}{N_{ds}(g_j)}$.

$$\Rightarrow (I - B)Acc = B(\triangle E)$$

$$\Rightarrow Acc = (I - B)^{-1}B(\triangle E).$$

Thus, we had $ta \leq |ta| = |\text{sum}(Acc)| \leq \|Acc\|_1 = \|B(I - B)^{-1} \triangle E\|_1 \leq \|B(I - B)^{-1}\|_1 \|\triangle E\|_1$.

$$\Rightarrow -1 \leq t_{AN} = \frac{ta}{\|B(I - B)^{-1}\|_1 \|\triangle E\|_1} \leq 1$$

as normalized eSPIA.

Text S3. Post analysis (peak identification)

The following prompts are designed to perform m/z comparisons and identify compounds of 12 drugs in our narcotics study by using several popular AI chatbots, including ChatGPT-4, ChatGPT-3.5, Llama3-70b, and Windows Copilot (Precision Mode). According to our experience, it's highly suggested that prompts should be concise, structured, and outlined step by step rather than described in a whole paragraph without a clear segmentation.

> You are a mass spectrometer expert.
> You are also knowledgeable about narcotics.
>
> We have 12 drugs to identify, including heroin (diacetylmorphine), morphine, cocaine, thebaine, delta9-THC (delta9-tetrahydrocannabinol), amphetamine, MA (methamphetamine), MDMA (3,4-methylenedioxymethamphetamine), MDA (also known as the love drug; 3,4-methylenedioxyamphetamine), ketamine, FM2 (flunitrazepam), and nimetazepam.
>
> First, list the molecular formulas and masses of these 12 drugs.
>
> The closest match for each peak to the 12 drugs was found by calculating the difference in ppm (delta value) between the m/z value corresponding to each compound's true mass and the provided peak. This ensures the most accurate match.
>
> I will give you the compounds that correspond to the following MS1 m/z [M+H].
> Please calculate and list the delta value calculation process one by one.
>
> Which of the 12 drugs listed above corresponds to the MS1 data below?
> Peak index m/z
> 1 136.1118
> 2 150.1273
> 3 180.1017
> 4 194.1172
> 5 238.0992
> 6 286.143

7	296.1025
8	304.1538
9	312.1586
10	314.093
11	315.2318
12	370.1642

Please calculate one by one.

Please help me find the following information in the database: compound names, compound masses, compound formulas, delta values, and the databases used.

Text S4. Post analysis (concentration calibration)

1. Calibration curve construction

   In the process of concentration calibration, the initial step involved the construction of a calibration curve. This was achieved by first using standard substances and conducting experiments to collect MS1 and MS2 data. Subsequently, targeted peak analysis was applied to calculate the peak abundance of the standard substances.

   The concentration calibration, a part of the post-analysis, then proceeded to construct the calibration curve. This involved the use of either a linear model or a quadratic model to create. The 'nls' function of R was employed to calculate the nonlinear (weighted) least-squares estimates of the parameters for a nonlinear model. Various weight methods (1, 1/x, or $1/x^2$) and outlier detection techniques (Cook's D, CI, and Bias) were employed to enhance the accuracy of the calibration.

   Finally, all results were generated and included model parameter estimates for each combination of weight and outlier detection methods. Additionally, relevant information such as R^2 and adjusted R^2, and parameter estimates of calibration curve were provided in the output.

2. Concentration calculation

   To estimate the concentration of an unknown sample, users could employ their own calibration curve. This required providing the parameter estimates of the model, the accurate concentration of the standards, and the standards' intensity. Alternatively, users could input the calibration curve results generated in the previous stage. SMART took into account user settings, such as choosing between a linear or quadratic model, weighting methods, and outlier detection methods, and used adjusted R^2 to automatically select the most suitable model. The parameter estimates from this selected model were then applied to estimate the concentration of the unknown sample.

Text S5. Drug experiment

1. Chemicals

The drug standards were purchased from Cerilliant (Round Rock, TX, USA). Acetonitrile (ACN, LC-MS reagent) and formic acid (reagent grade) were obtained from J. T. Baker and Honeywell (Muskegon, MI, USA), respectively. Deionized water was produced by a Milli-Q system (Millipore, Bedford, MA, USA).

2. Standard solution preparation

The stock solution including 12 drug standards (20 ppm, each) was prepared in 5% ACN, which was further diluted to different concentrations (i.e., 50, 100, 200, 300, 400, 500, 600, 700, 800, 900, and 1000 ppb) for analysis.

3. Instrumentation and setting parameters

The samples were analyzed by LC-MS/MS. An ultra-performance liquid chromatographic system (Ultimate 3000, ThermoFisher Scientific, Germany) with a Kinetex Biphenyl (2.1 mm × 100 mm, 2.6 µm, 100 Å, Phenomenex, USA) column was used for separation. The injection volume was set at 20 µL and the column oven temperature was maintained at 25 oC. The flow rate was set at 0.8 mL/min. The mobile phases including D.D. water with 0.1% formic acid (eluent A) and ACN with 0.1% formic acid (eluent B) were used. The LC multistep gradient started at 0% B over 5 min, and sequentially ramped to 40% B over 10 min, 75% over 5 min, 95% over 5 min, and then decreased to 0% B in 2 min. The initial gradient composition (0% B) was then restored and allowed to equilibrate for 2 min before the next measurement. An Orbitrap Fusion Lumos Tribrid mass spectrometer (Thermo Fisher Scientific, USA) equipped with a heated electrospray ionization (HESI) source was used to collect mass spectra at MS1 (full scan: 50–550 m/z) and MS2 (DDA mode) level. The ESI voltage was +3.5 kV, the temperature of ion transfer tube and sheath gas was set to 330 and 320 oC, respectively. The pressure of sheath gas, aux gas, sweep gas were respectively set to 60, 15, and 2 arbitrary units in the control software.

SUPPLEMENTAL TABLES

Table S1. Performance metrics of PLS-DA models based on different feature selection strategies applied to gene expression (GE) and metabolites (MT). For each selection criterion, the number of selected genes and metabolites, total features used, and the variance explained by the first two latent variables (t1 and t2) are shown. Model performance was evaluated using *R2X* (variance explained in predictors), *R2Y* (variance explained in response), *Q2Y* (predictive accuracy via cross-validation), and RMSEE (root mean square error of estimation). All models were constructed using the ropls package. The optimal model in terms of *Q2Y* is highlighted in bold.

GE & MT selection criteria	# GE	# MT	# Total	t1	t2	*R2X*	*R2Y*	*Q2Y*	RMSEE
All GE	20254	0	20254	14%	9%	0.237	0.685	0.539	0.282
All MT	0	504	504	34%	5%	0.395	0.594	0.454	0.322
All GE + MT	20254	504	20758	14%	9%	0.236	0.694	0.55	0.278
pFDR<0.05	9227	343	9570	28%	6%	0.341	0.705	0.594	0.273
pFDR<0.05 Top200	200	200	400	63%	7%	0.693	0.683	0.602	0.283
pFDR<0.05 Top100	100	100	200	70%	7%	0.765	0.67	0.61	0.289
pFDR<0.05 Top50	50	50	100	73%	7%	0.806	0.667	0.63	0.29
pFDR<0.05 Top30	30	30	60	75%	8%	0.834	0.669	0.64	0.289
pFDR<0.05 & VIP>=1	3820	221	4041	43%	6%	0.489	0.716	0.648	0.268
pFDR<0.05 & VIP>=1.5	236	23	259	71%	4%	0.75	0.724	0.671	0.264
pFDR<0.05 & VIP Top200	200	200	400	59%	5%	0.648	0.722	0.658	0.265
pFDR<0.05 & VIP Top100	100	100	200	65%	6%	0.703	0.722	0.67	0.265
pFDR<0.05 & VIP Top50	50	50	100	67%	7%	0.744	0.713	0.675	0.269
pFDR<0.05 & VIP Top30	30	30	60	70%	8%	0.775	0.719	0.689	0.267
pFDR<0.05 & VIP Top20	20	20	40	70%	7%	0.775	0.729	0.696	0.262
**pFDR<0.05 & VIP Top10**	**10**	**10**	**20**	**70%**	**9%**	**0.788**	**0.725**	**0.701**	**0.263**
pFDR<0.05 & VIP Top5	5	5	10	70%	11%	0.812	0.718	0.695	0.267

Table S2. List of significant genes and metabolites with top 10 VIP scores.

Data	Rank_VIP	Name	pFDR_ANCOVA	VIP	p_VIP	Full Name
GE	1	*DENND2A*	5.77E-17	1.749866	3.64E-15	DENN domain containing 2A
GE	2	*VIT*	5.77E-17	1.74309	1.07E-14	vitrin
GE	3	*SEMA3G*	1.62E-16	1.706197	1.83E-14	semaphorin 3G
GE	4	*SYN2*	2.47E-16	1.724991	2.11E-14	synapsin II
GE	5	*TMEM220*	3.37E-15	1.716255	4.33E-14	transmembrane protein 220
GE	6	*FIGF*	5.91E-16	1.70247	6.33E-14	vascular endothelial growth factor D
GE	7	*NLGN1*	5.91E-16	1.712405	1.01E-13	neuroligin 1
GE	8	*PYROXD2*	1.20E-13	1.742309	3.23E-13	pyridine nucleotide-disulphide oxidoreductase domain 2
GE	9	*WISP1*	8.60E-12	1.754814	3.39E-13	WNT1-inducible signaling pathway protein 1
GE	10	*BGN*	5.01E-10	1.827453	2.09E-10	biglycan
MT	1	C-glycosyltryptophan	1.08E-15	1.805684	5.75E-14	tryptophan 2-C-mannoside
MT	2	dimethylarginine (SDMA + ADMA)	8.78E-15	1.674783	1.86E-13	Symmetric dimethylarginine, Asymmetric dimethylarginine
MT	3	phosphoethanolamine	1.79E-14	1.659897	2.69E-13	2-Amino-ethanol dihydrogen phosphate
MT	4	proline	1.79E-14	1.666299	3.09E-13	proline
MT	5	glutamate	2.83E-14	1.634288	3.89E-13	glutamate
MT	6	aspartate	5.62E-14	1.66305	6.42E-13	aspartic acid
MT	7	X - 8994	1.19E-13	1.662857	2.15E-12	NA
MT	8	N-acetylaspartate (NAA)	3.69E-12	1.654768	9.29E-12	N-Acetylaspartic acid
MT	9	taurine	1.72E-12	1.639344	9.54E-12	taurine
MT	10	X - 12800	7.13E-11	1.624311	7.84E-11	NA

SUPPLEMENTAL FIGURES

Figure S1. Comparison of SMART 2.0 and 1.0. The changes and new features introduced in the upgrade from SMART 1.0 to SMART 2.0 are highlighted in blue.

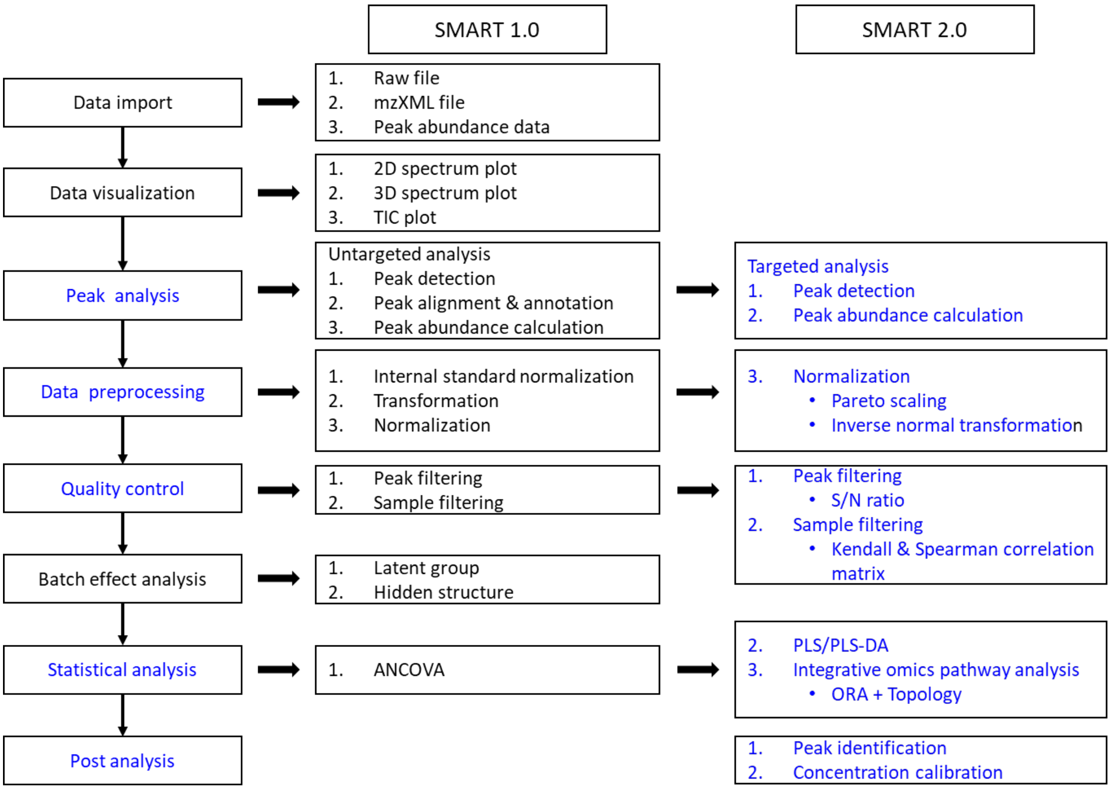

Figure S2A. MS1 and MS2 spectra of amphetamine at a concentration of 500 ppb. (a) MS1. (b) MS2.

(a)

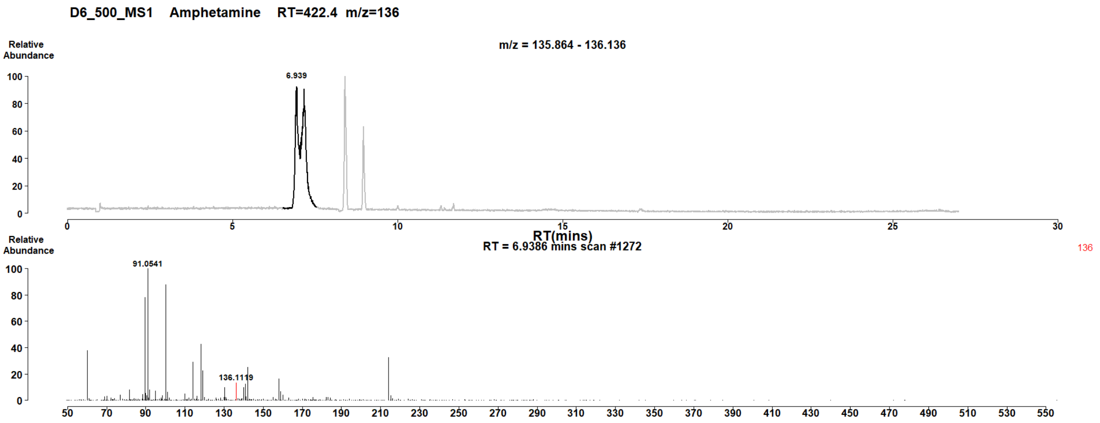

(b)

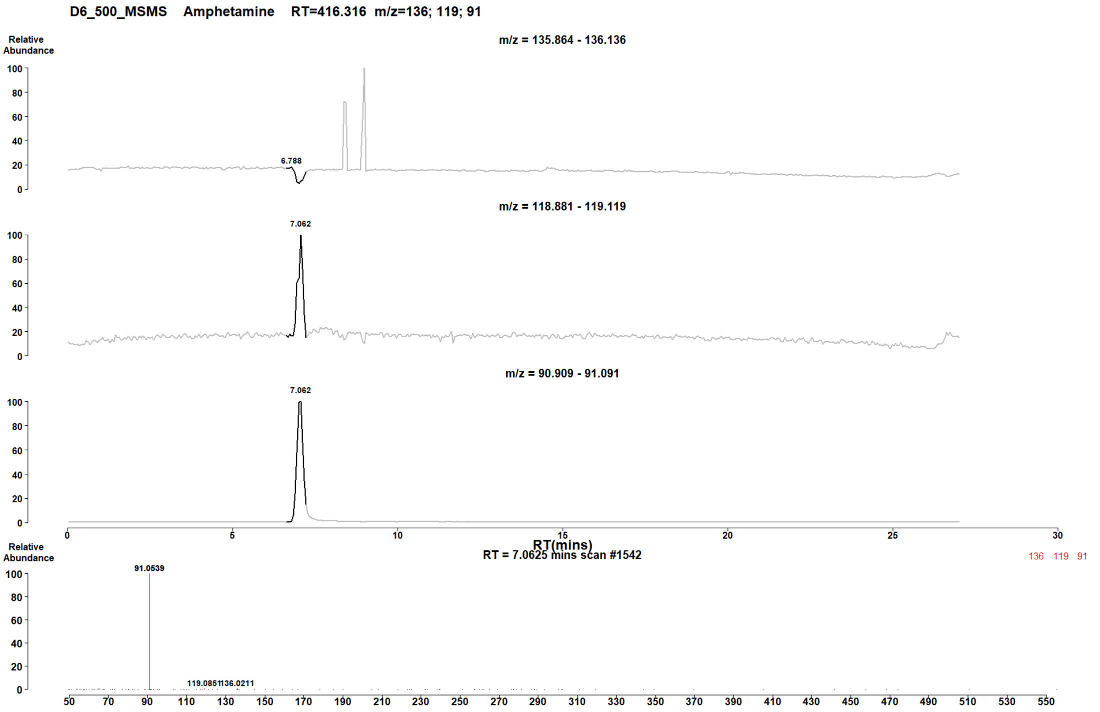

Figure S2B. MS1 and MS2 spectra of cocaine at a concentration of 500 ppb. (a) MS1. (b) MS2.

(a)

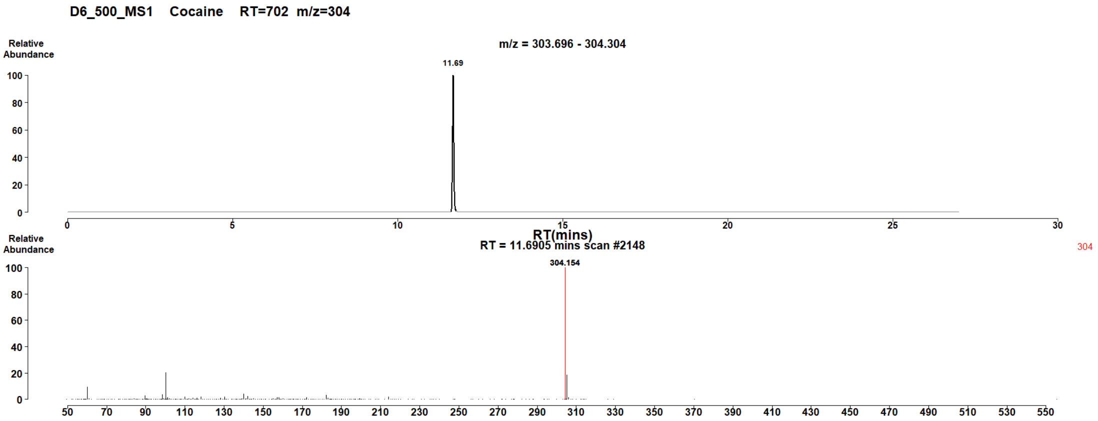

(b)

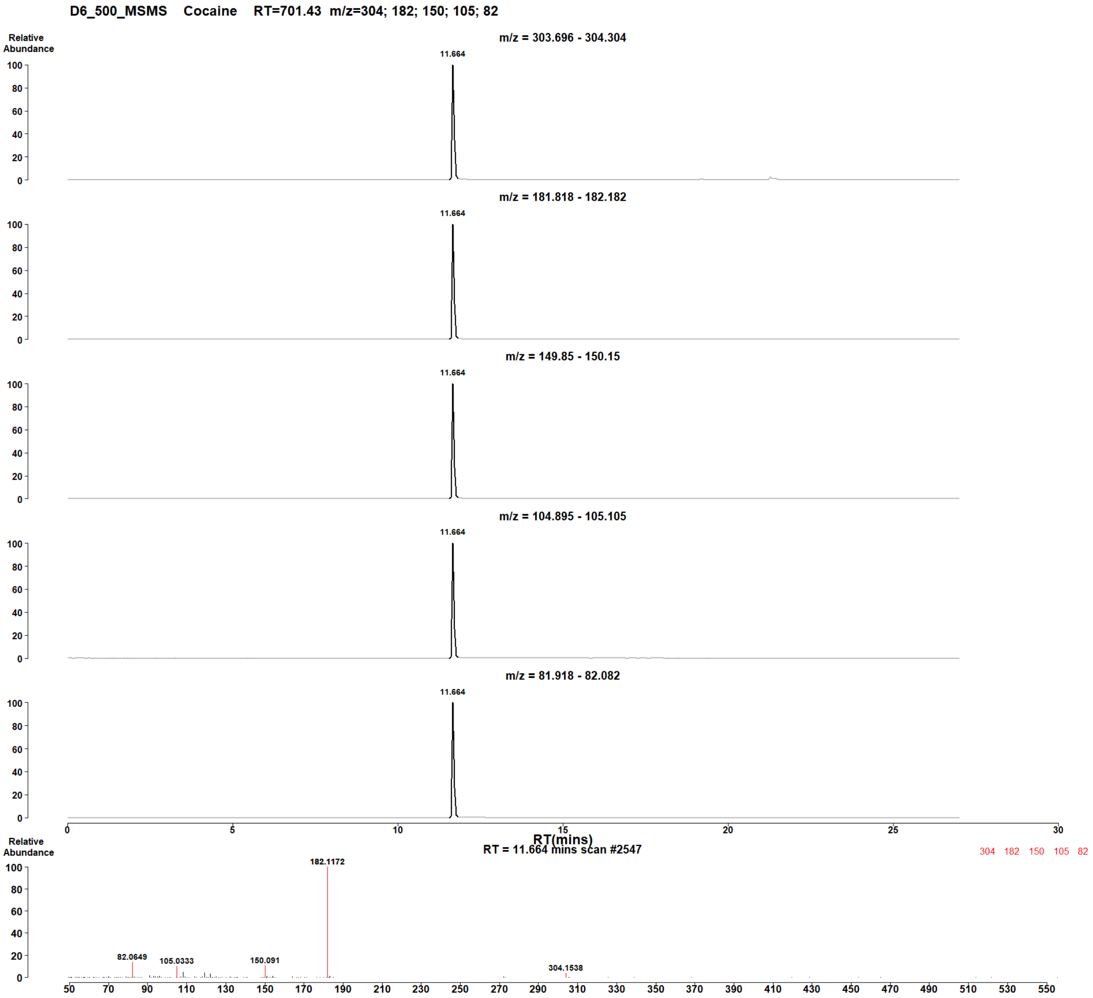

Figure S2C. MS1 and MS2 spectra of delta9-THC at a concentration of 500 ppb. (a) MS1. (b) MS2.

(a)

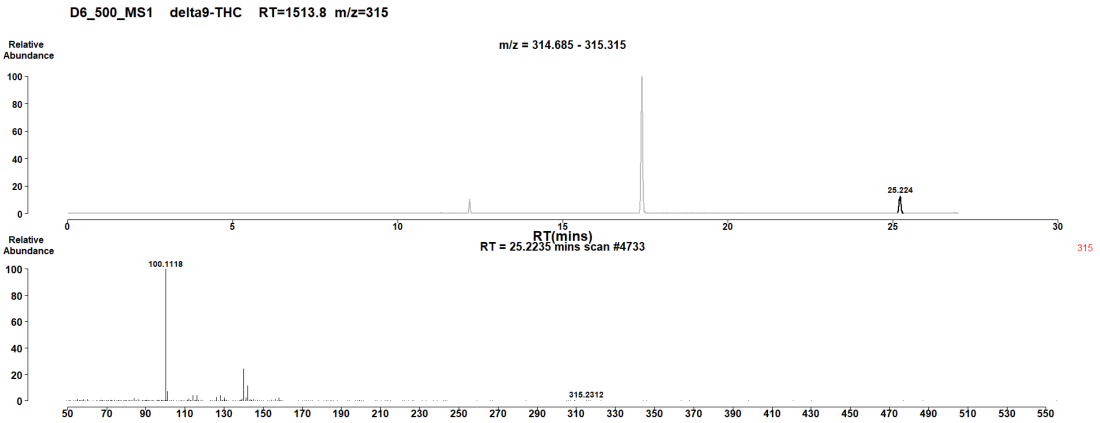

(b)

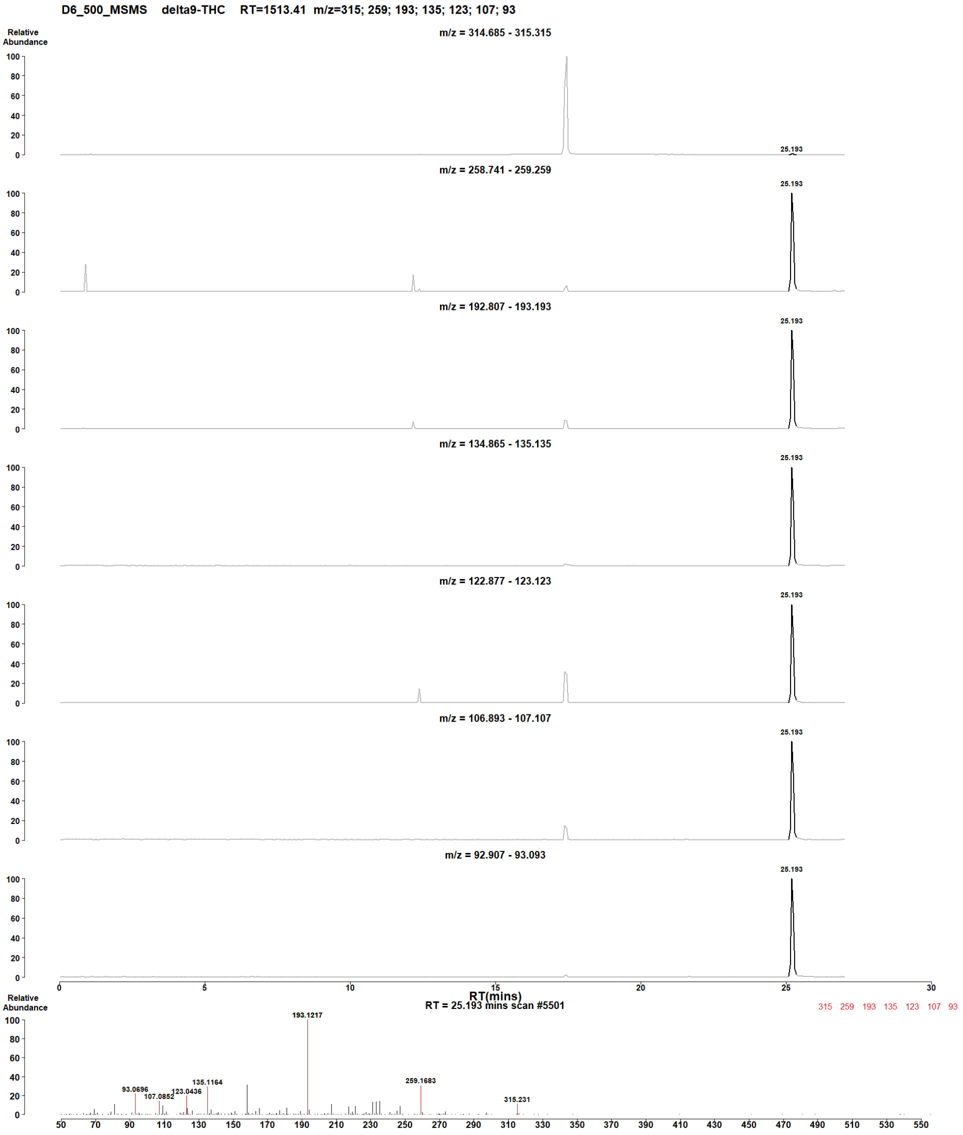

Figure S2D. MS1 and MS2 spectra of FM2 at a concentration of 500 ppb. (a) MS1. (b) MS2.

(a)

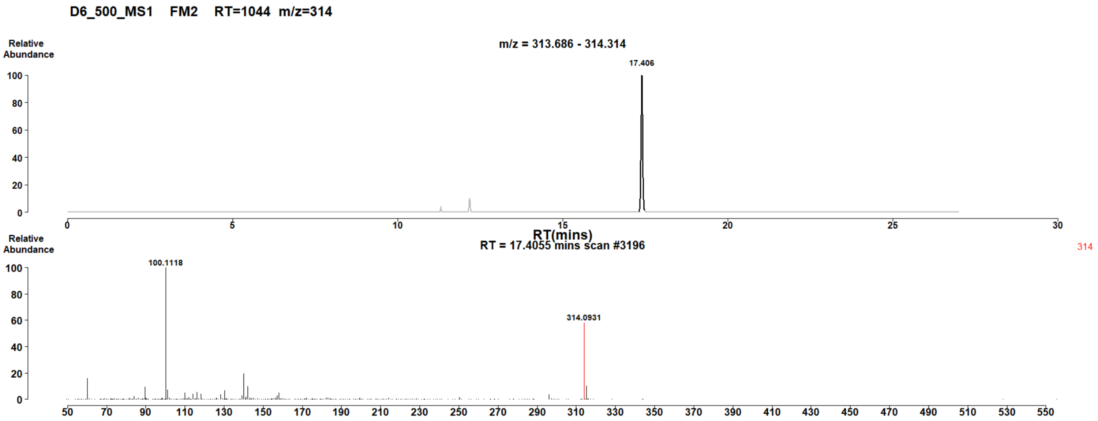

(b)

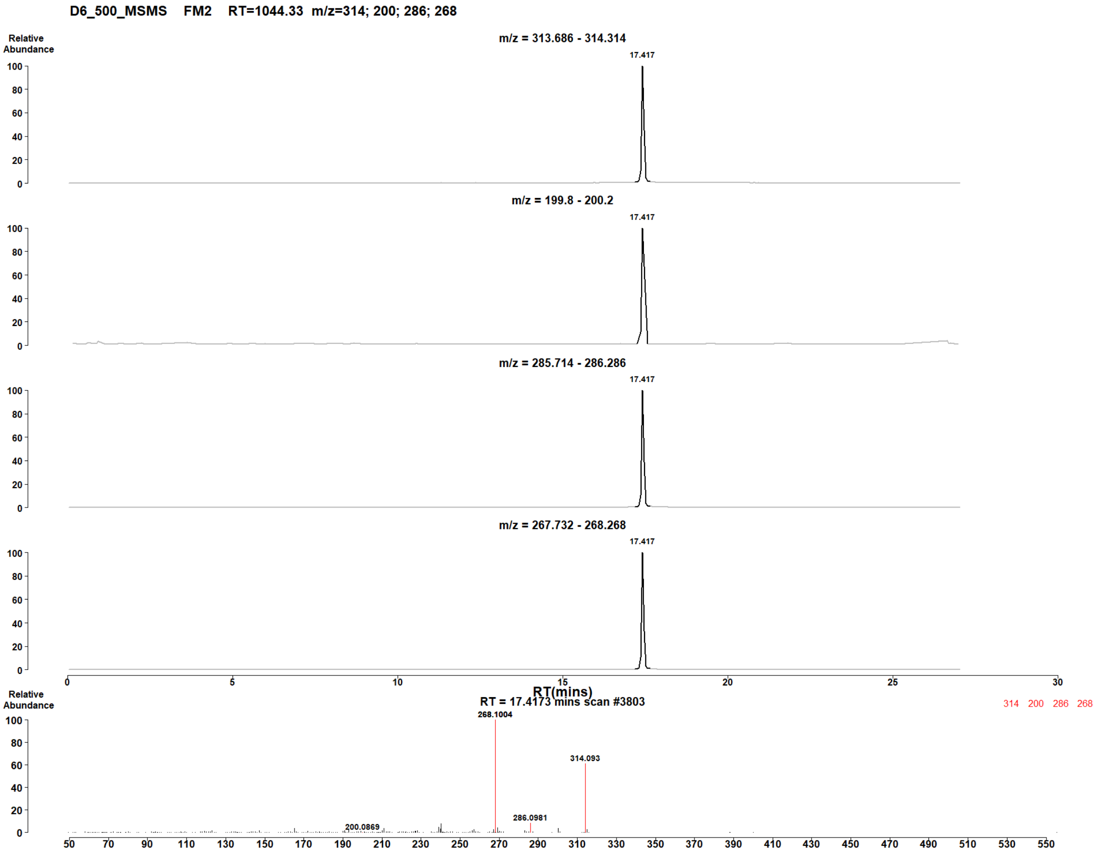

Figure S2E. MS1 and MS2 spectra of heroin at a concentration of 500 ppb. (a) MS1. (b) MS2.

(a)

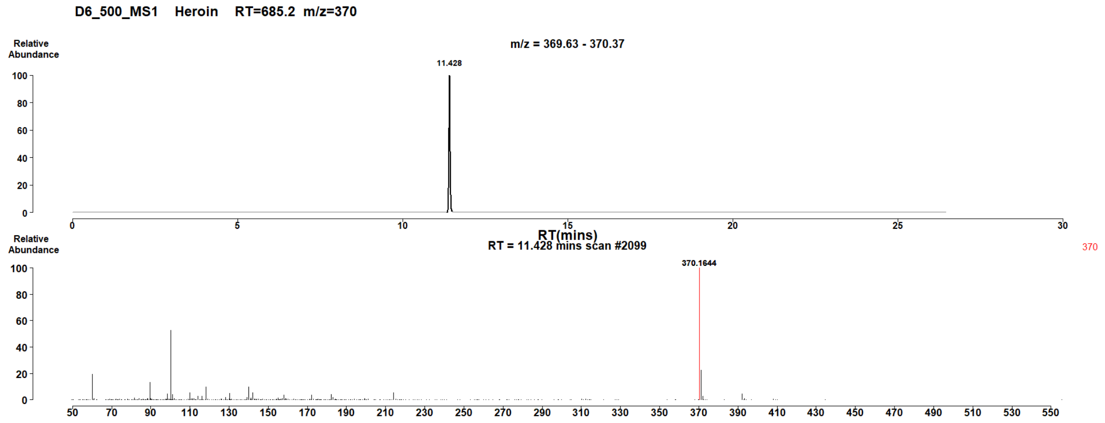

(b)

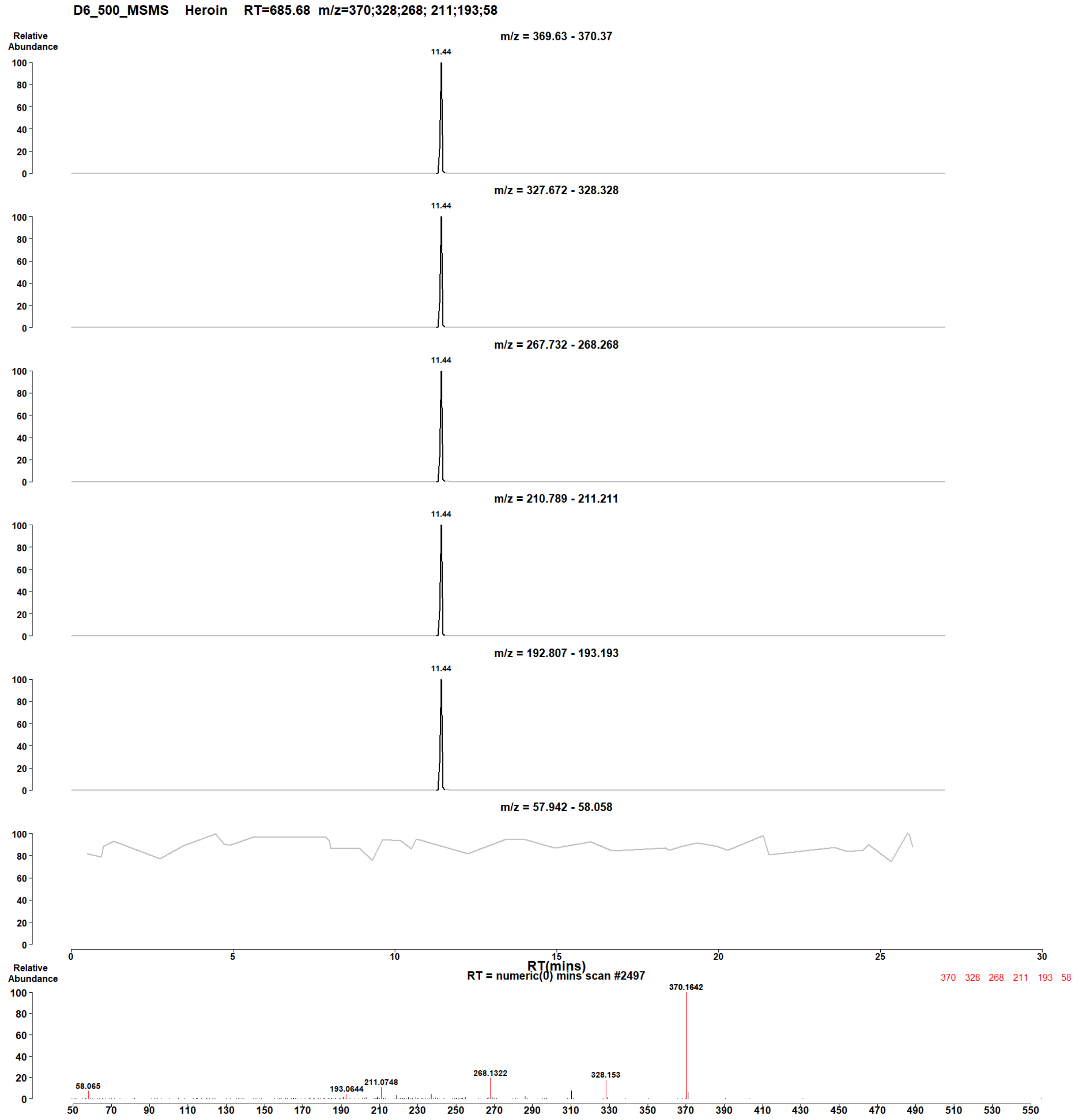

Figure S2F. MS1 and MS2 spectra of ketamine at a concentration of 500 ppb. (a) MS1. (b) MS2.

(a)

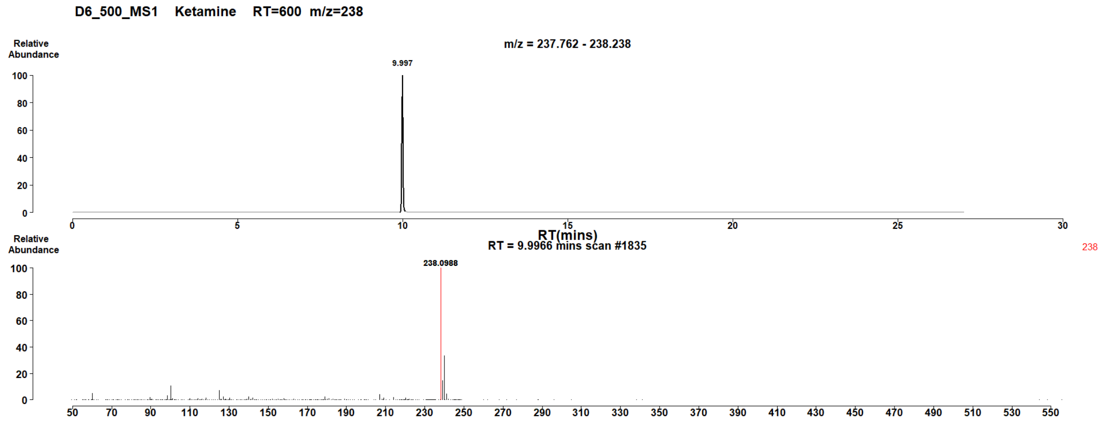

(b)

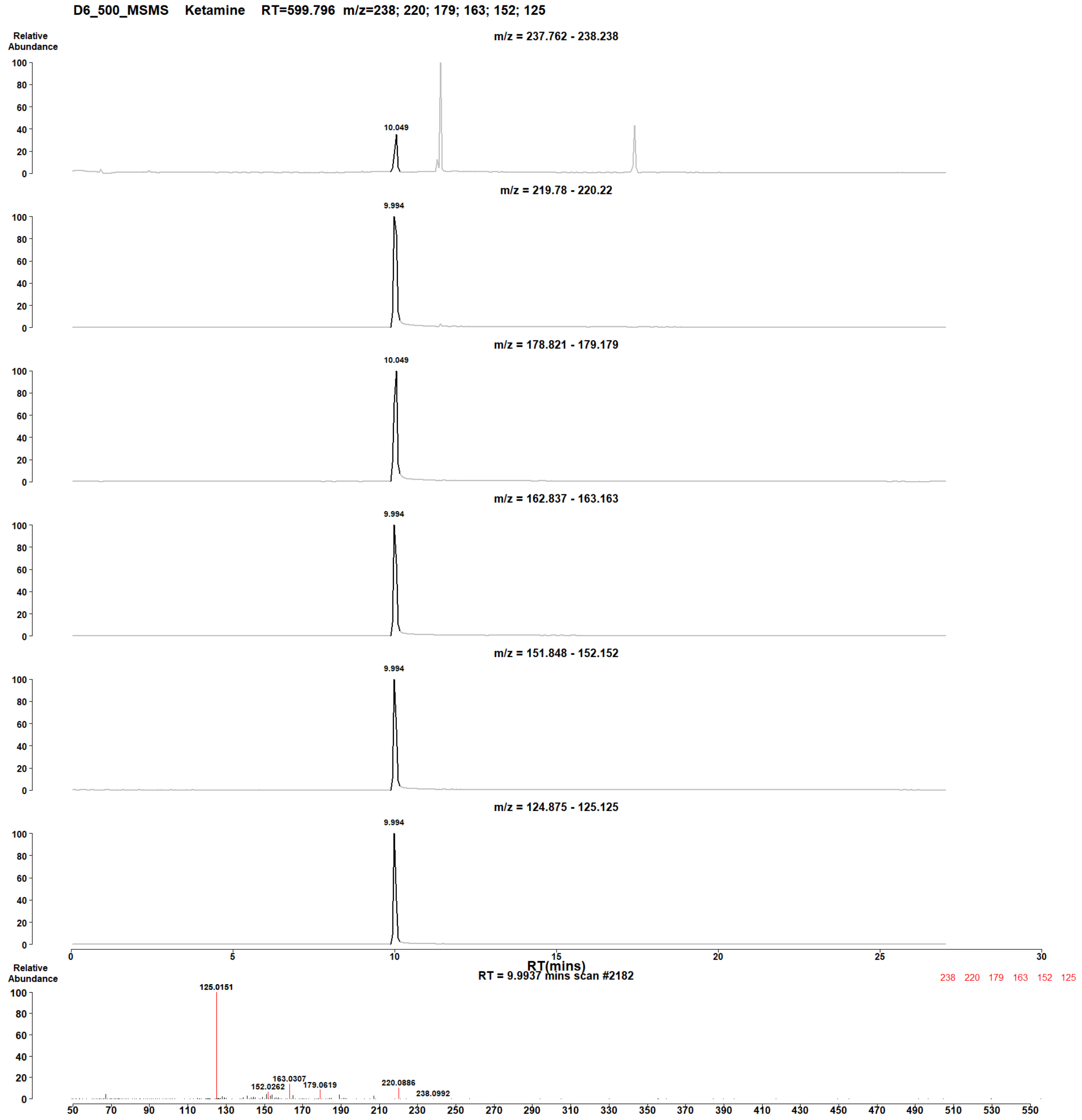

Figure S2G. MS1 and MS2 spectra of MDA (love drug) at a concentration of 500 ppb. (a) MS1. (b) MS2.

(a)

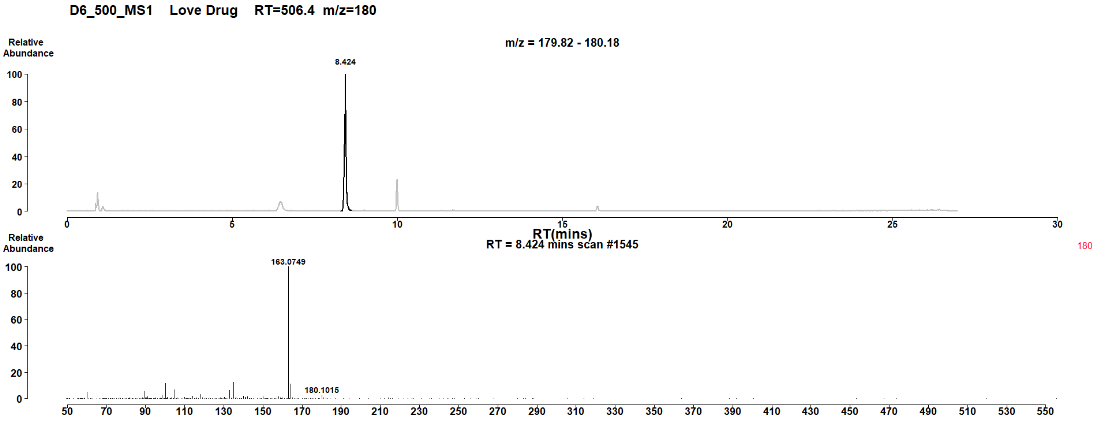

(b)

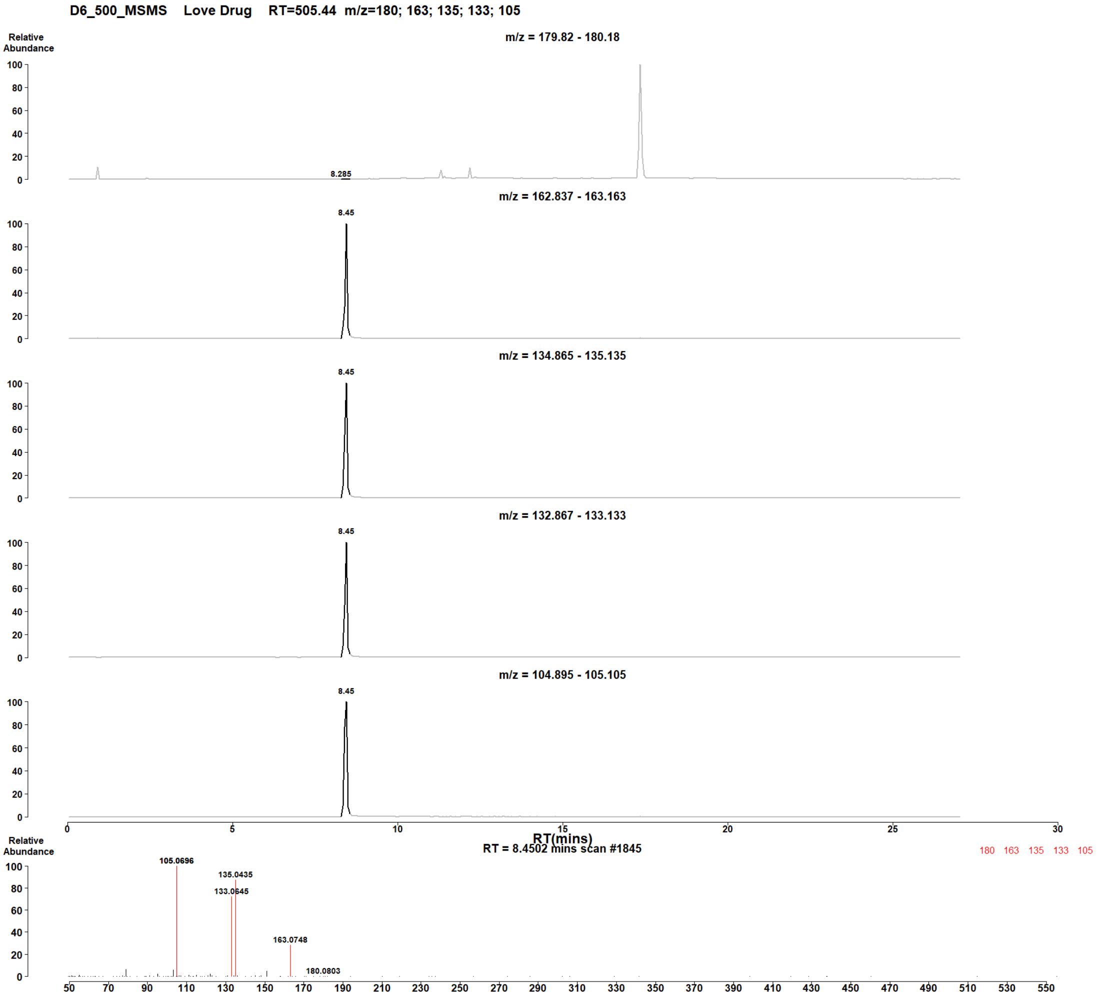

Figure S2H. MS1 and MS2 spectra of MA at a concentration of 500 ppb. (a) MS1. (b) MS2.

(a)

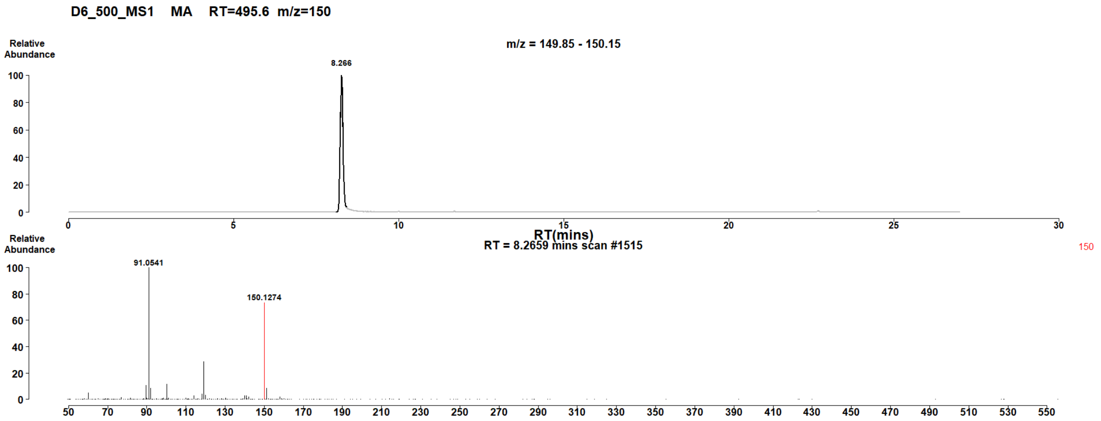

(b)

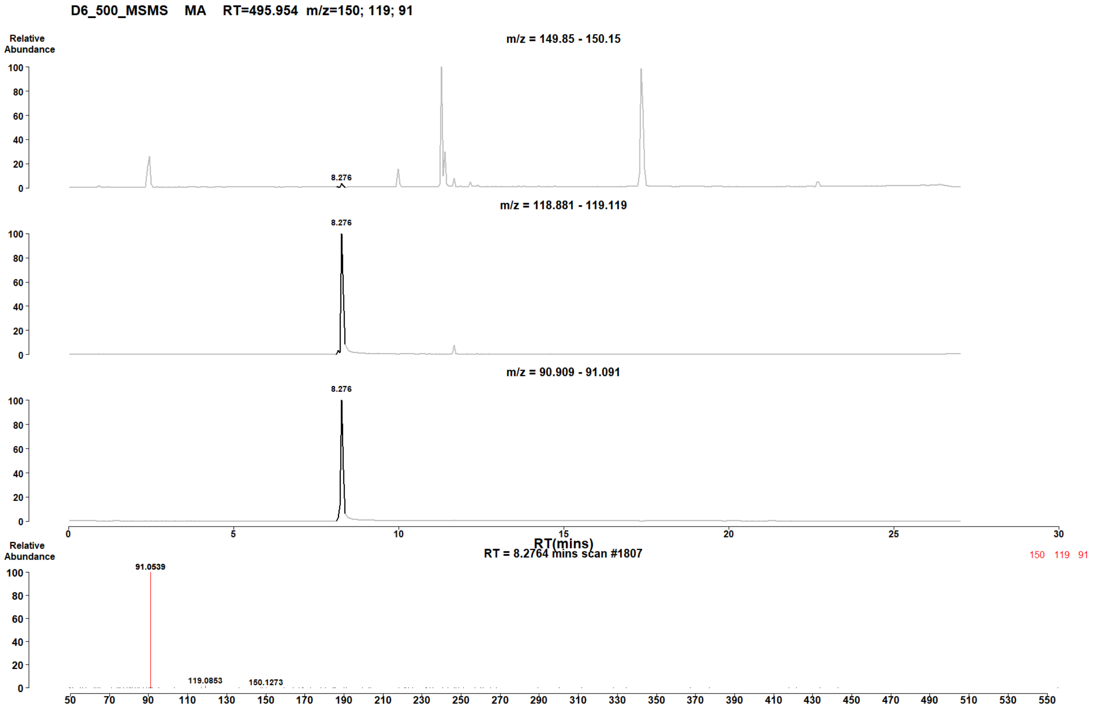

Figure S2I. MS1 and MS2 spectra of MDMA at a concentration of 500 ppb. (a) MS1. (b) MS2.

(a)

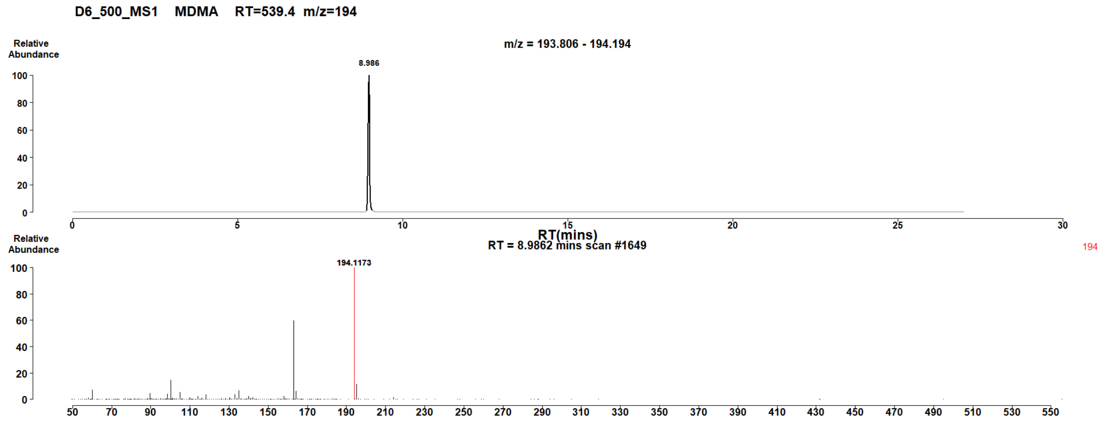

(b)

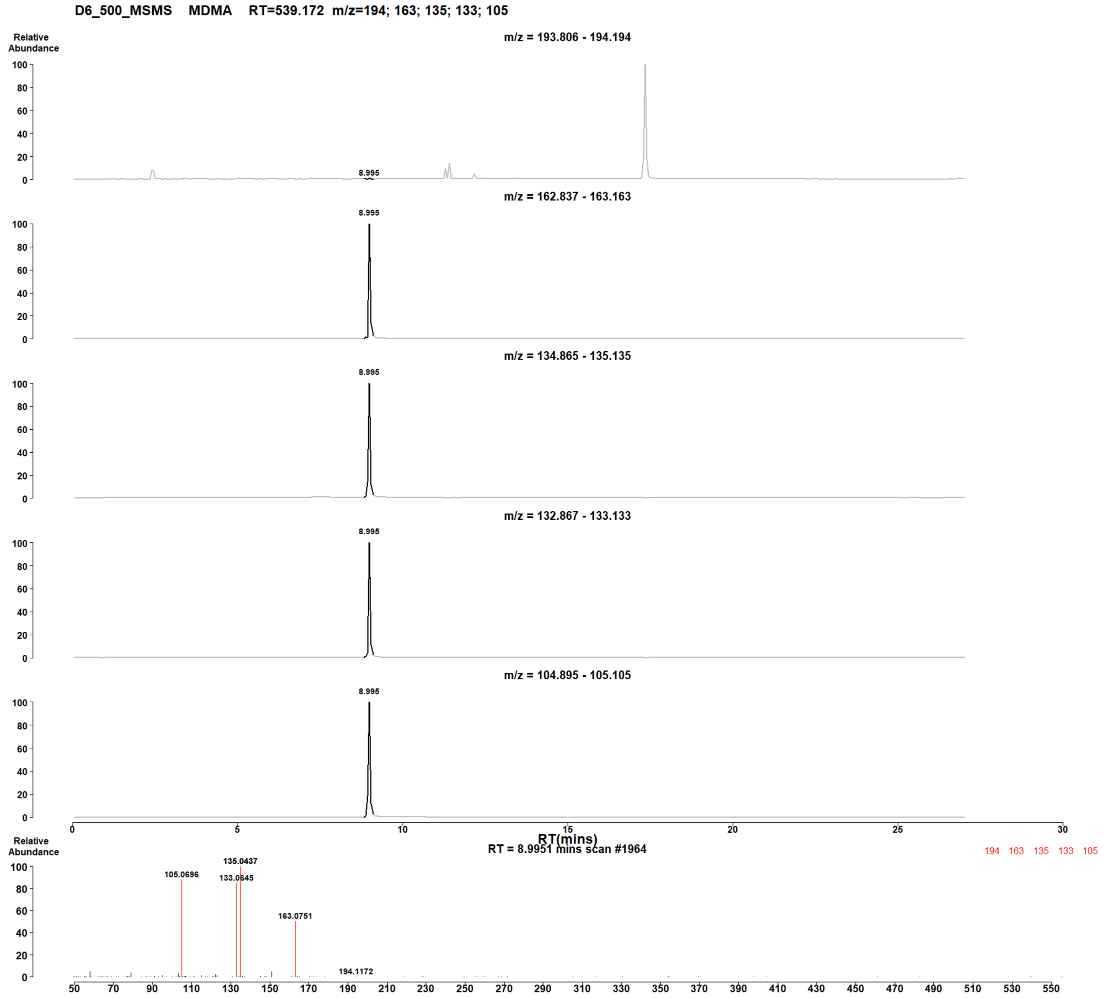

Figure S2J. MS1 and MS2 spectra of morphine at a concentration of 500 ppb. (a) MS1. (b) MS2.

(a)

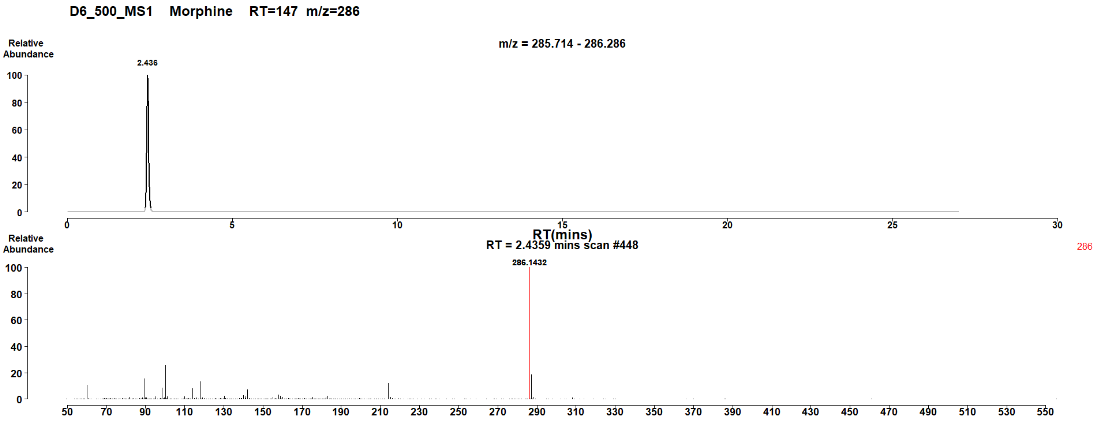

(b)

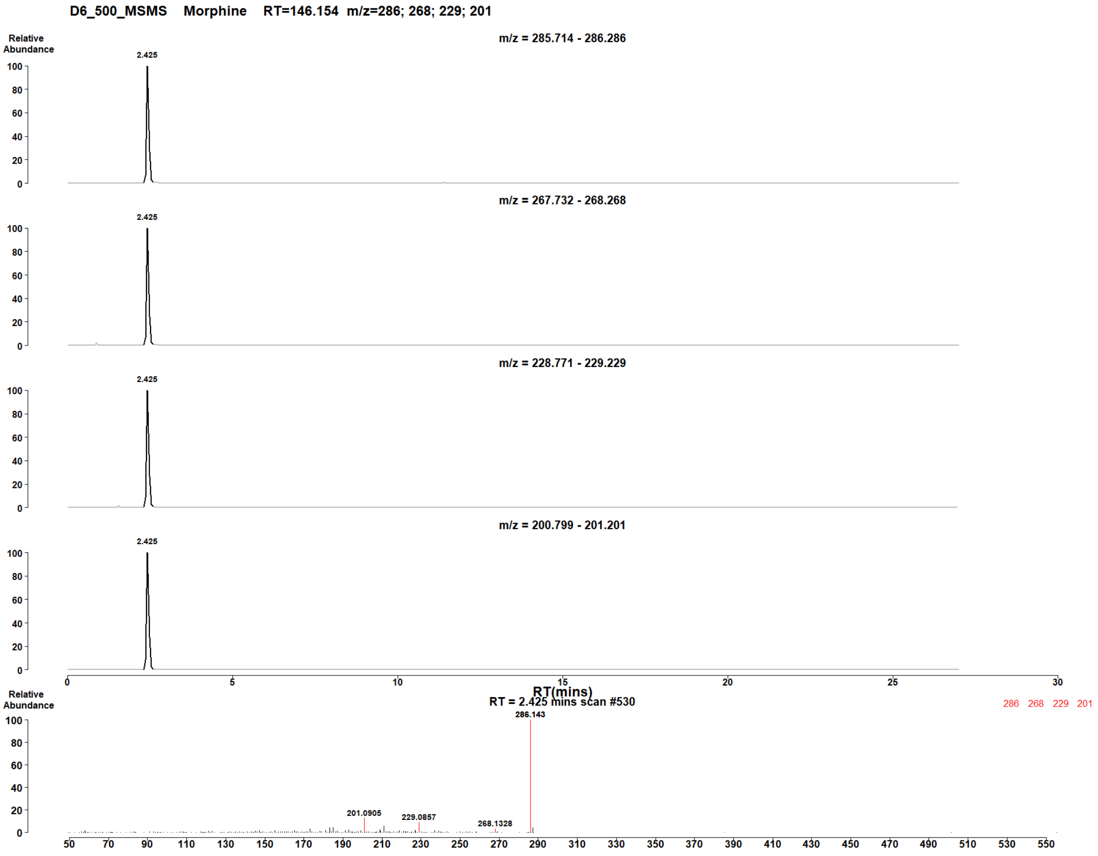

Figure S2K. MS1 and MS2 spectra of nimetazepam at a concentration of 500 ppb. (a) MS1. (b) MS2.

(a)

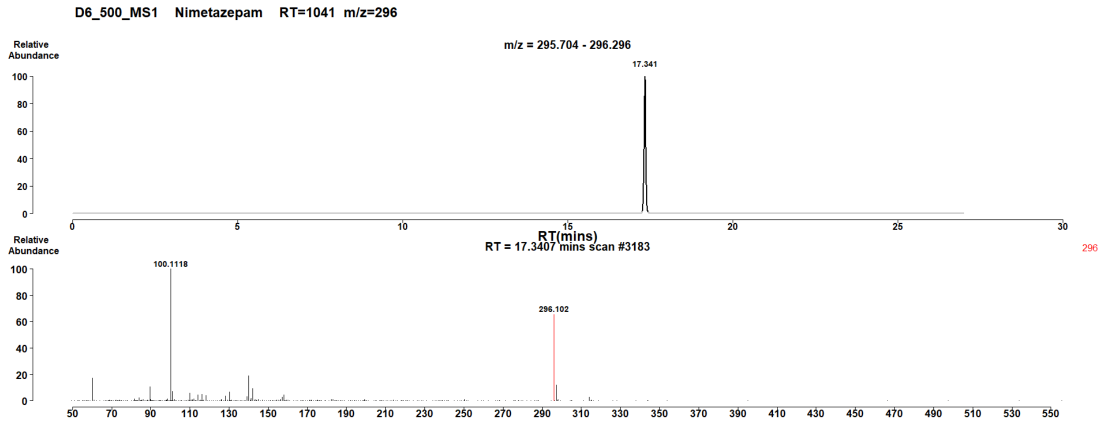

(b)

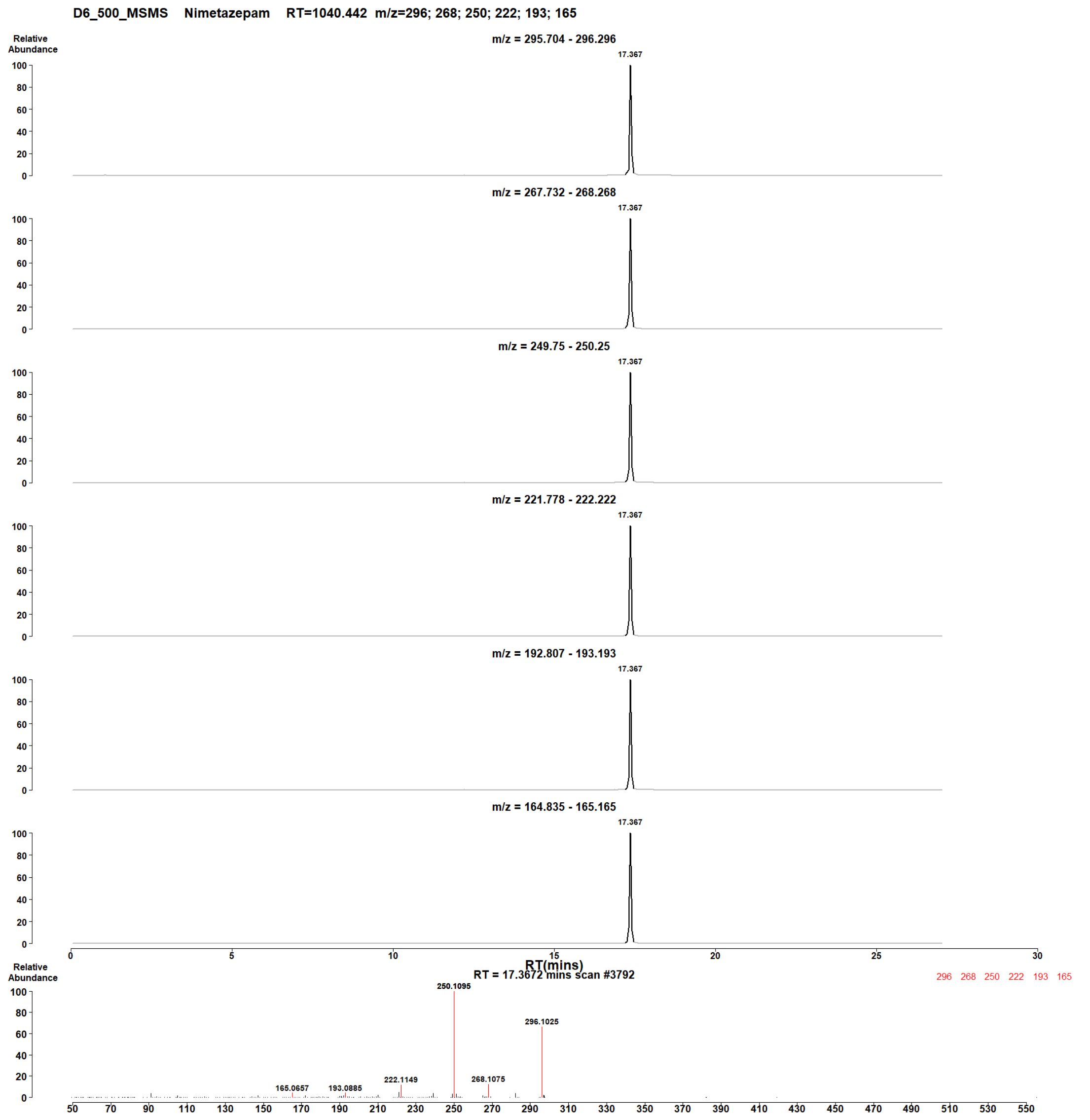

Figure S2L. MS1 and MS2 spectra of thebaine at a concentration of 500 ppb. (a) MS1. (b) MS2.

(a)

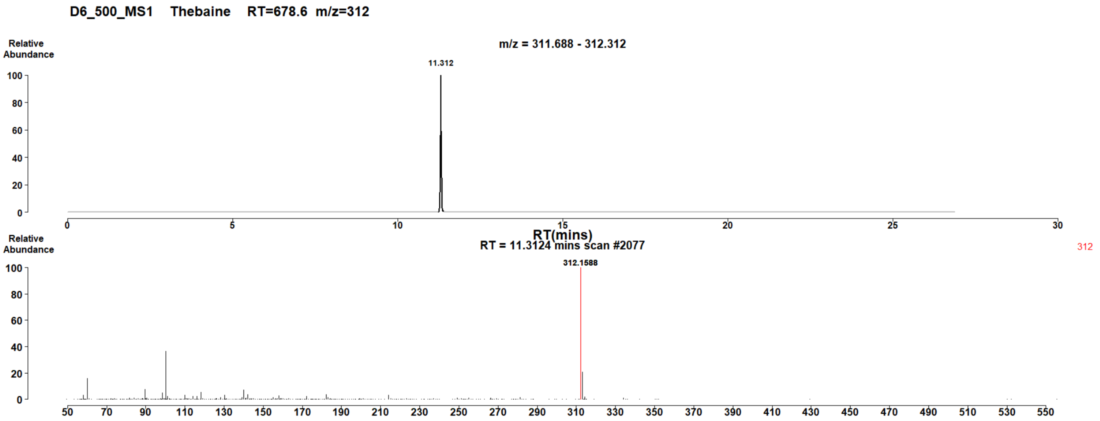

(b)

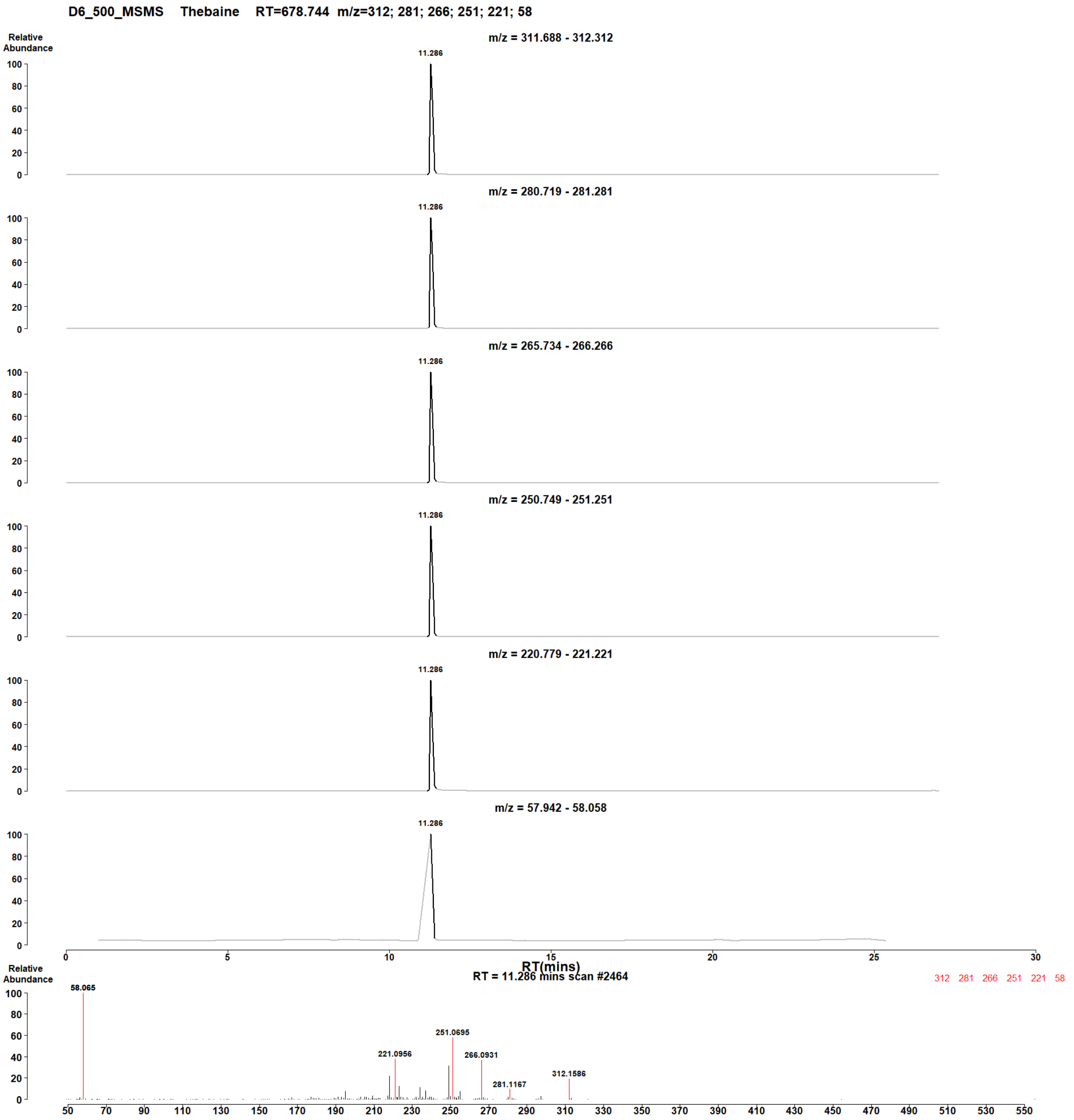

Figure S3A. Score plot from PLS-DA of gene expression data. Red denotes breast cancer samples, blue signifies normal samples, and black represents all samples. Ellipses denote 95% confidence intervals.

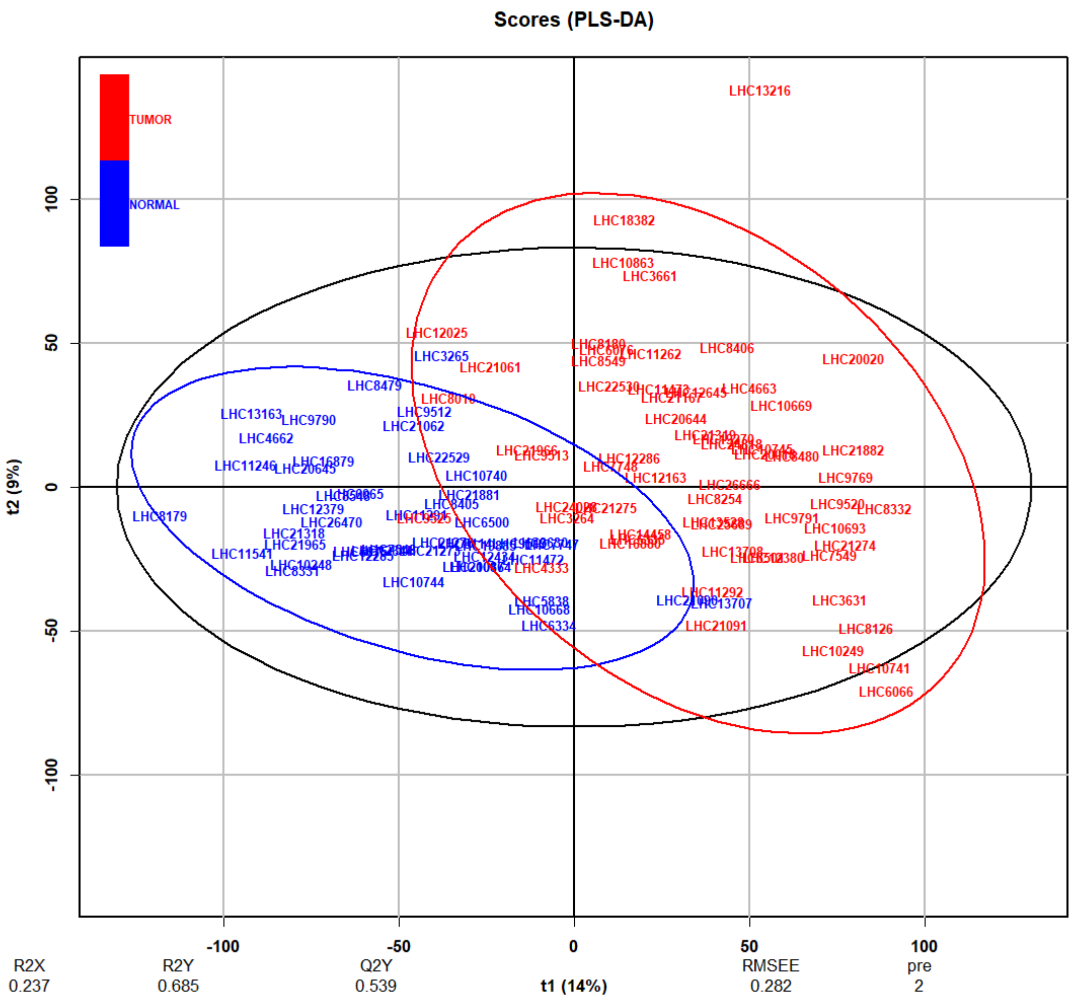

Figure S3B. Score plot from PLS-DA of metabolomics data. Red denotes breast cancer samples, blue signifies normal samples, and black represents all samples. Ellipses denote 95% confidence intervals.

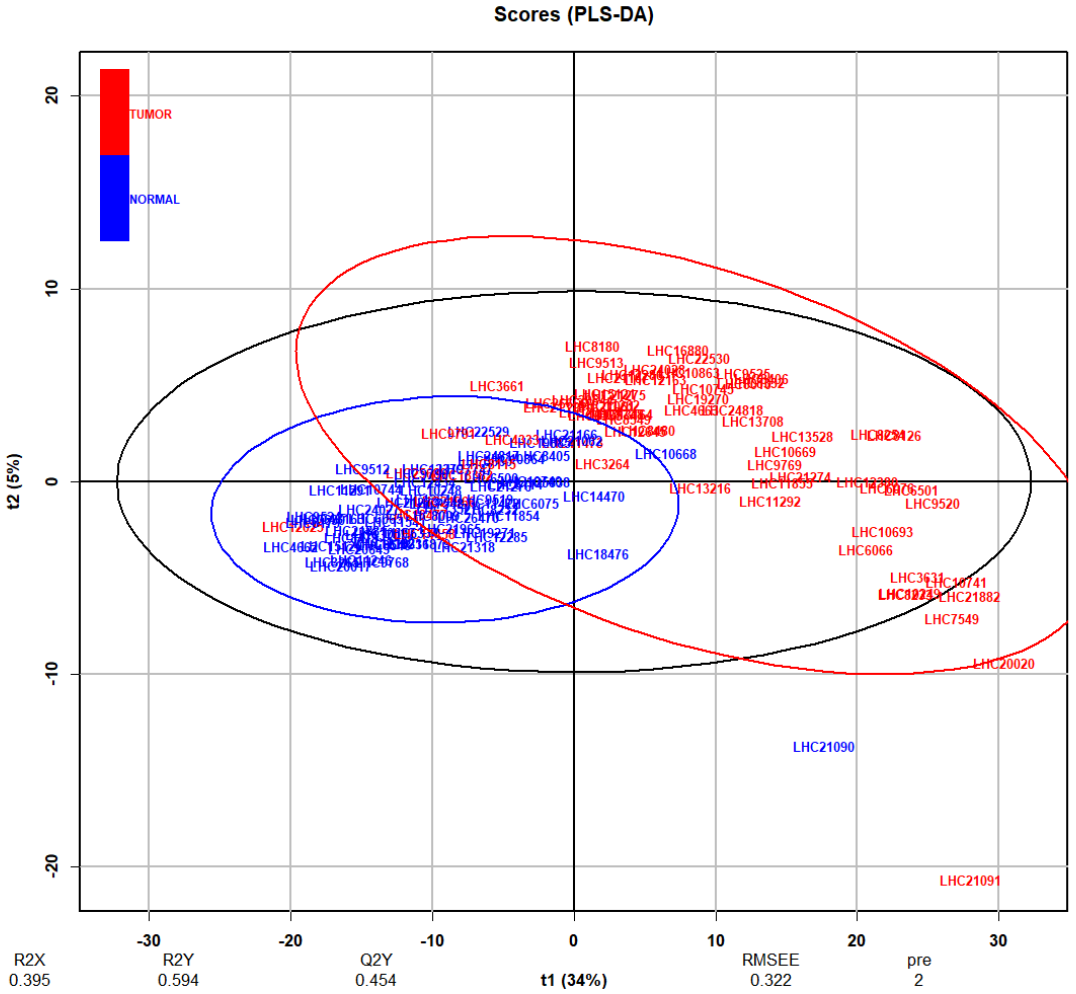

Figure S3C. Score plot from PLS-DA on integrated gene expression and metabolomics data.

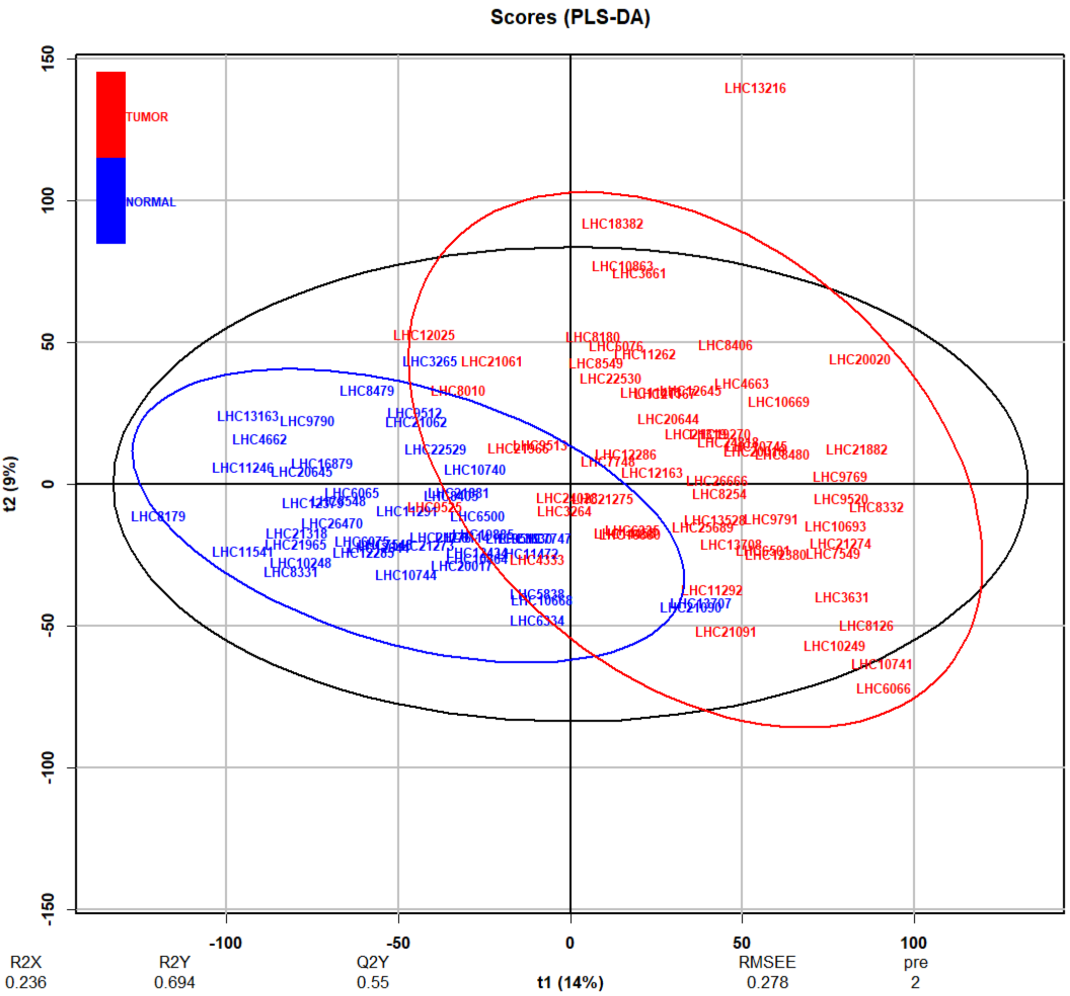

Figure S4. Volcano plot from the IOPA analysis of breast cancer data. The colors on the plot correspond to the KEGG pathway maps. The vertical axis represents the p-values of the Pbine test, which have been transformed to -log10 values after false discovery rate (FDR) correction. The horizontal axis represents the normalized eSPIA score, a statistical measure that takes into account both genes and metabolites within a pathway.

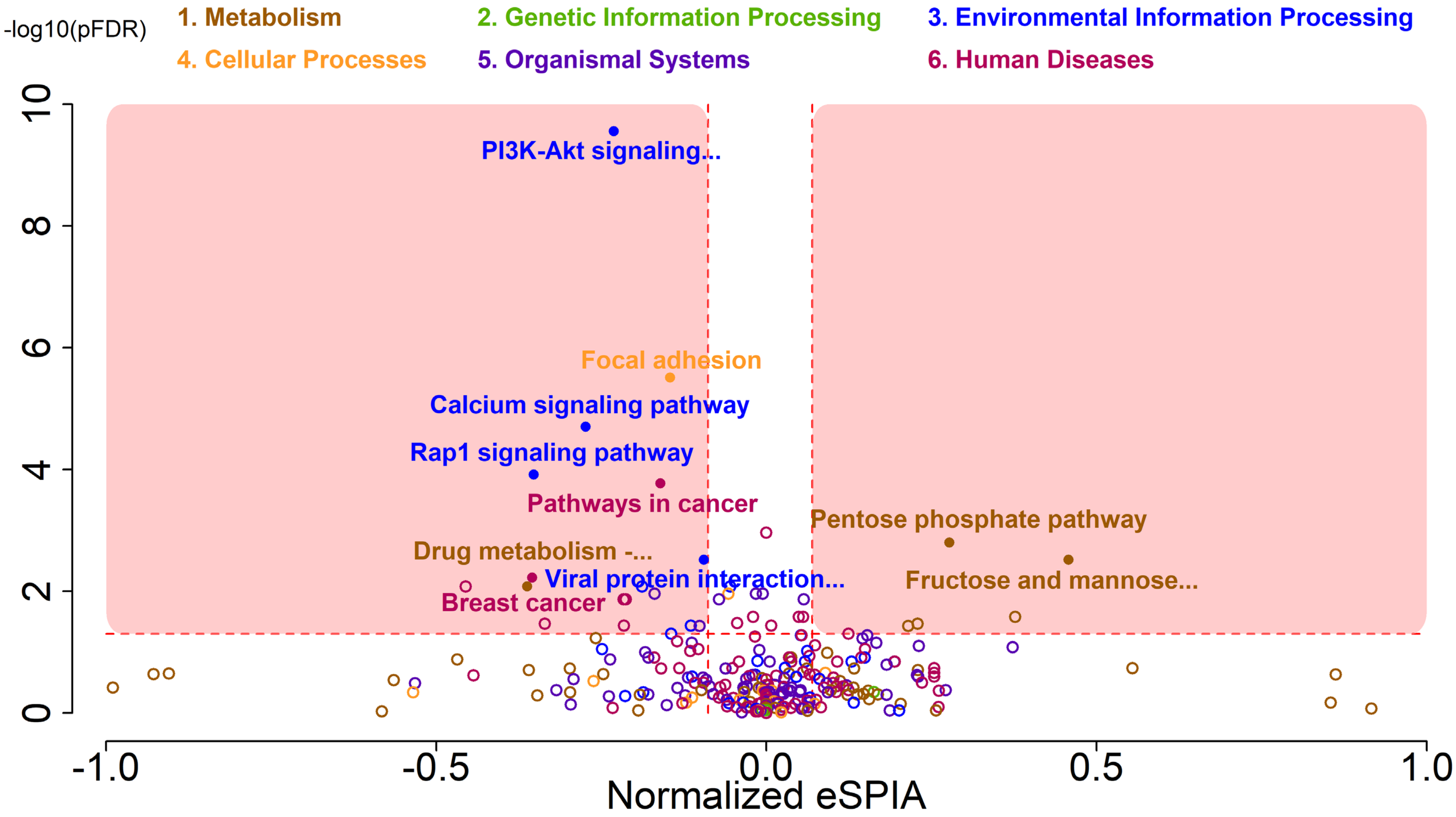

Figure S5A. Calibration curve for morphine. It was constructed using either a linear or quadratic model, with various weightings, including unweighted, 1/x, and 1/x^2. Additionally, different outlier detection methods were employed, including Cook's D, a 95% confidence interval (CI), and a Bias method (with a 20% relative error). (a) Linear model. (b) Quadratic model.

(a)

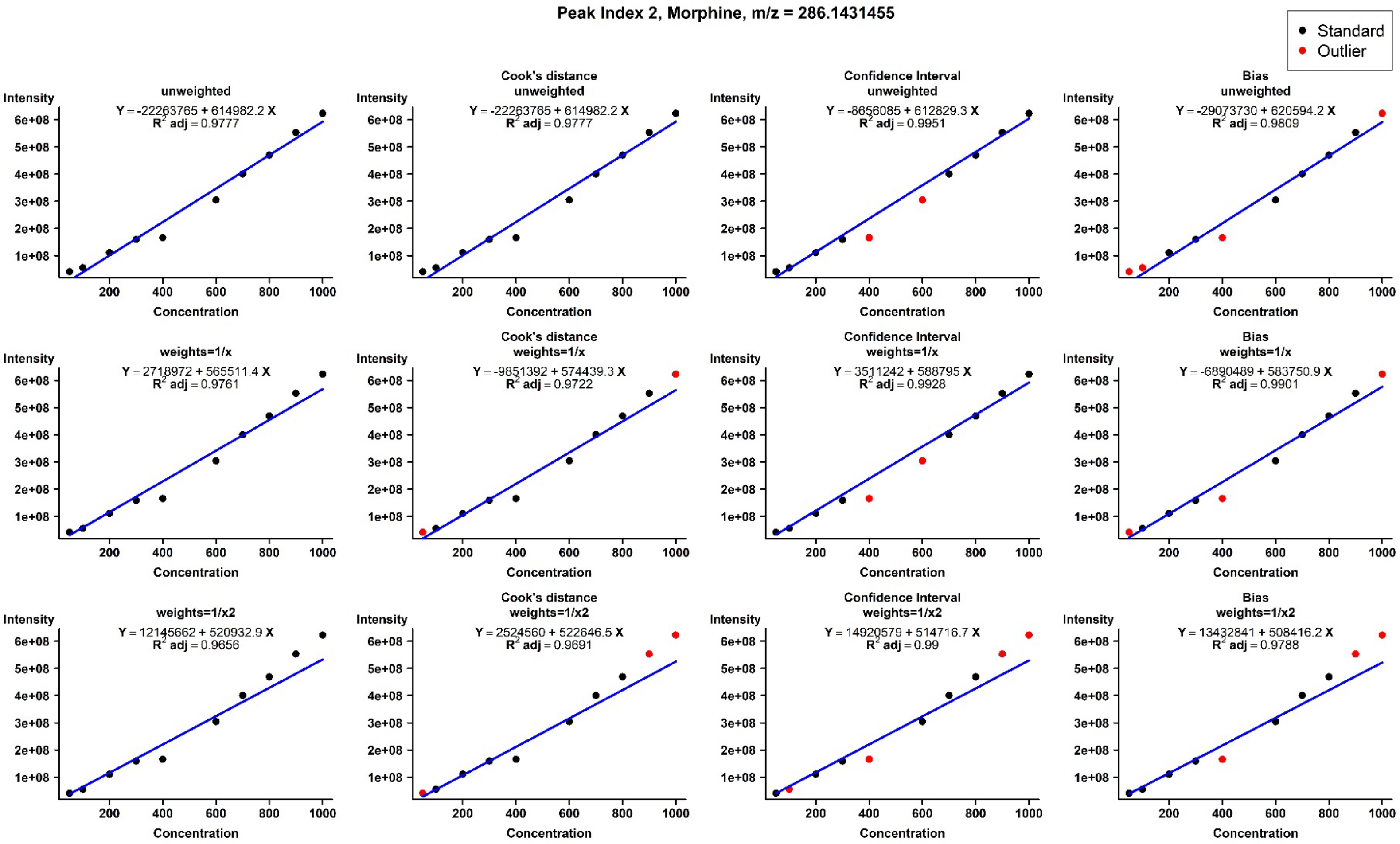

(b)

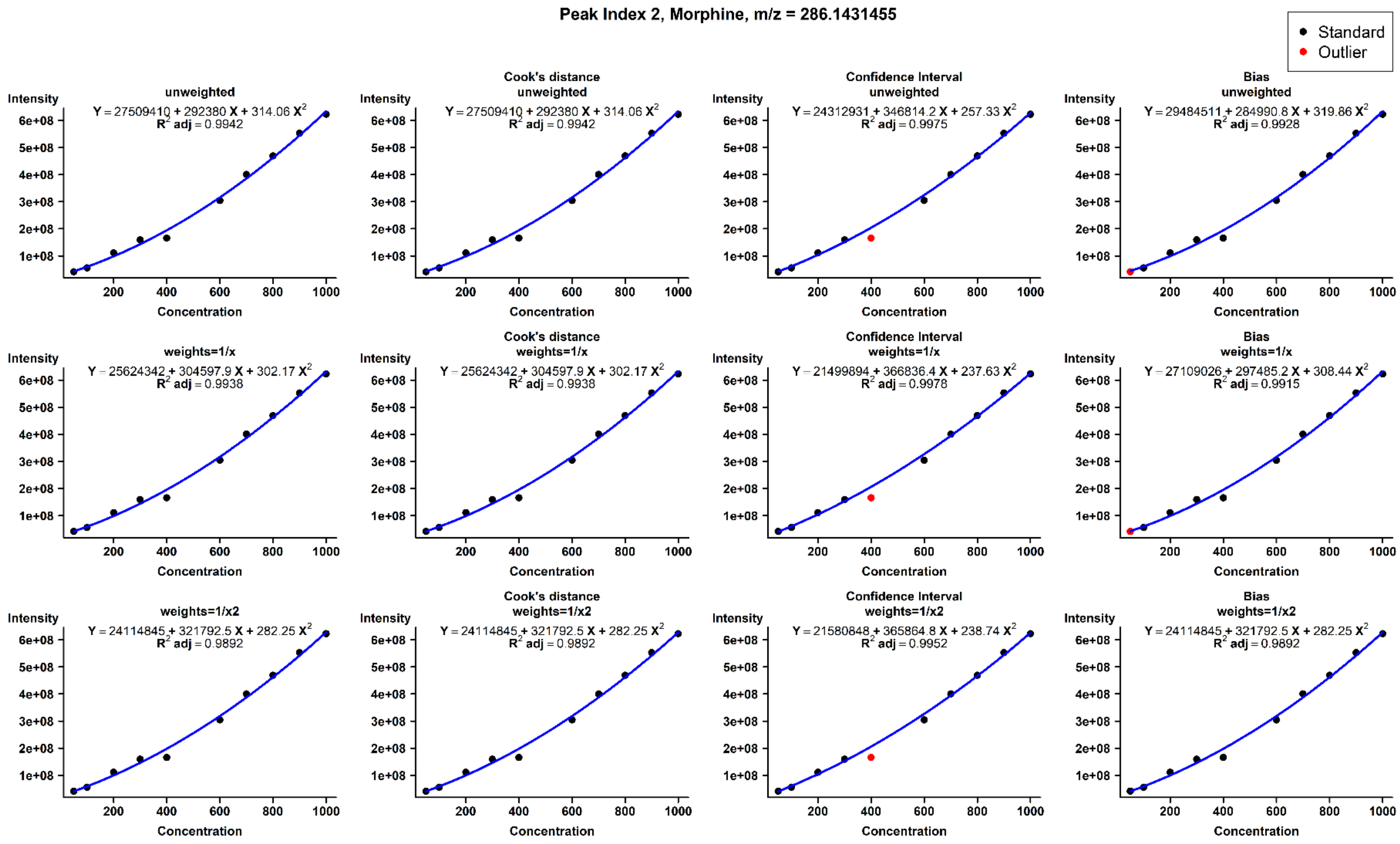

Peak Index 2, Morphine, m/z = 286.1431455
Standard
Outlier
unweighted
Y = 27509410 + 292380 X + 314.06 X2
R2 adj = 0.9942
Cook's distance
unweighted
Y = 27509410 + 292380 X + 314.06 X2
R2 adj = 0.9942
Confidence Interval
unweighted
Y = 24312931 + 346814.2 X + 257.33 X2
R2 adj = 0.9975
Bias
unweighted
Y = 29484511 + 284990.8 X + 319.86 X2
R2 adj = 0.9928
weights=1/x
Y = 25624342 + 304597.9 X + 302.17 X2
R2 adj = 0.9938
Cook's distance
weights=1/x
Y = 25624342 + 304597.9 X + 302.17 X2
R2 adj = 0.9938
Confidence Interval
weights=1/x
Y = 21499894 + 366836.4 X + 237.63 X2
R2 adj = 0.9978
Bias
weights=1/x
Y = 27109026 + 297485.2 X + 308.44 X2
R2 adj = 0.9915
weights=1/x2
Y = 24114845 + 321792.5 X + 282.25 X2
R2 adj = 0.9892
Cook's distance
weights=1/x2
Y = 24114845 + 321792.5 X + 282.25 X2
R2 adj = 0.9892
Confidence Interval
weights=1/x2
Y = 21580848 + 365864.8 X + 238.74 X2
R2 adj = 0.9952
Bias
weights=1/x2
Y = 24114845 + 321792.5 X + 282.25 X2
R2 adj = 0.9892
Intensity
Concentration

Figure S5B. Calibration curve for cocaine. It was constructed using either a linear or quadratic model, with various weightings, including unweighted, 1/x, and 1/x^2. Additionally, different outlier detection methods were employed, including Cook's D, a 95% confidence interval (CI), and a Bias method (with a 20% relative error). (a) Linear model. (b) Quadratic model.

(a)

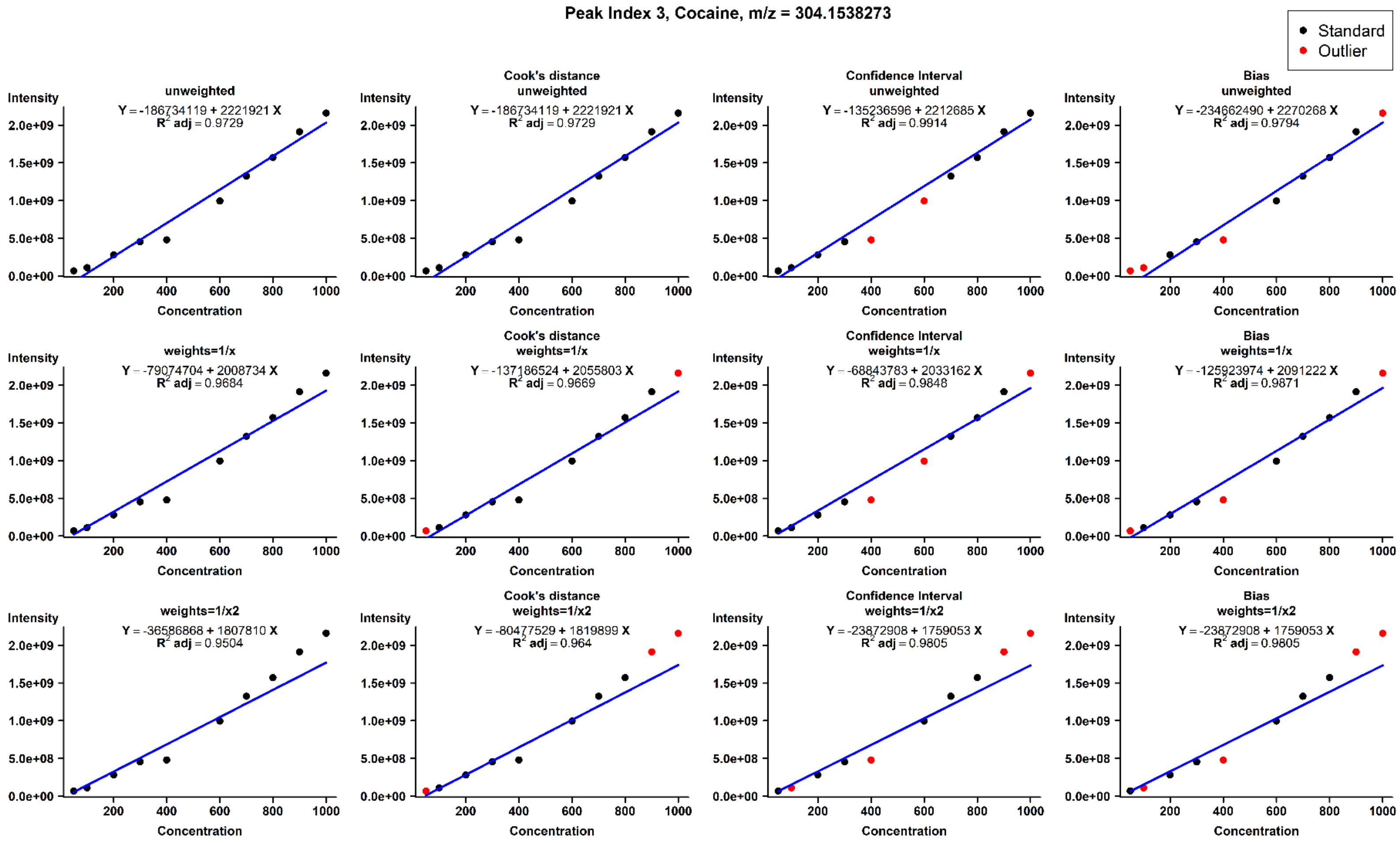

(b)

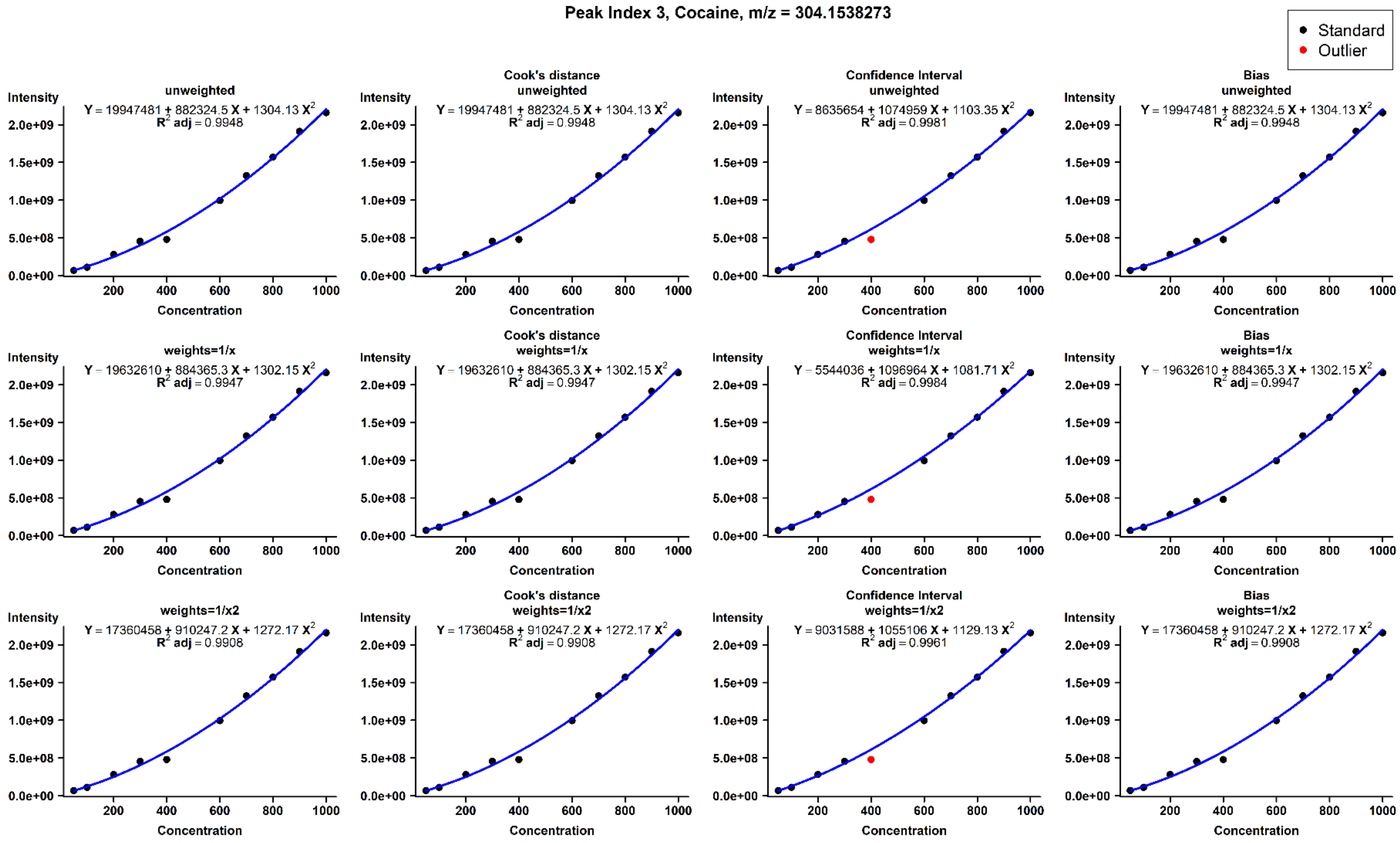
Peak Index 3, Cocaine, m/z = 304.1538273
Standard
Outlier
unweighted
Y = 19947481 + 882324.5 X + 1304.13 X2
R2 adj = 0.9948
Cook's distance
Confidence Interval
Y = 8635654 + 1074959 X + 1103.35 X2
R2 adj = 0.9981
Bias
weights=1/x
Y = 19632610 + 884365.3 X + 1302.15 X2
R2 adj = 0.9947
Y = 5544036 + 1096964 X + 1081.71 X2
R2 adj = 0.9984
weights=1/x2
Y = 17360458 + 910247.2 X + 1272.17 X2
R2 adj = 0.9908
Y = 9031588 + 1055106 X + 1129.13 X2
R2 adj = 0.9961
Intensity
Concentration

Figure S5C. Calibration curve for thebaine. It was constructed using either a linear or quadratic model, with various weightings, including unweighted, 1/x, and 1/x^2. Additionally, different outlier detection methods were employed, including Cook's D, a 95% confidence interval (CI), and a Bias method (with a 20% relative error). (a) Linear model. (b) Quadratic model.

(a)

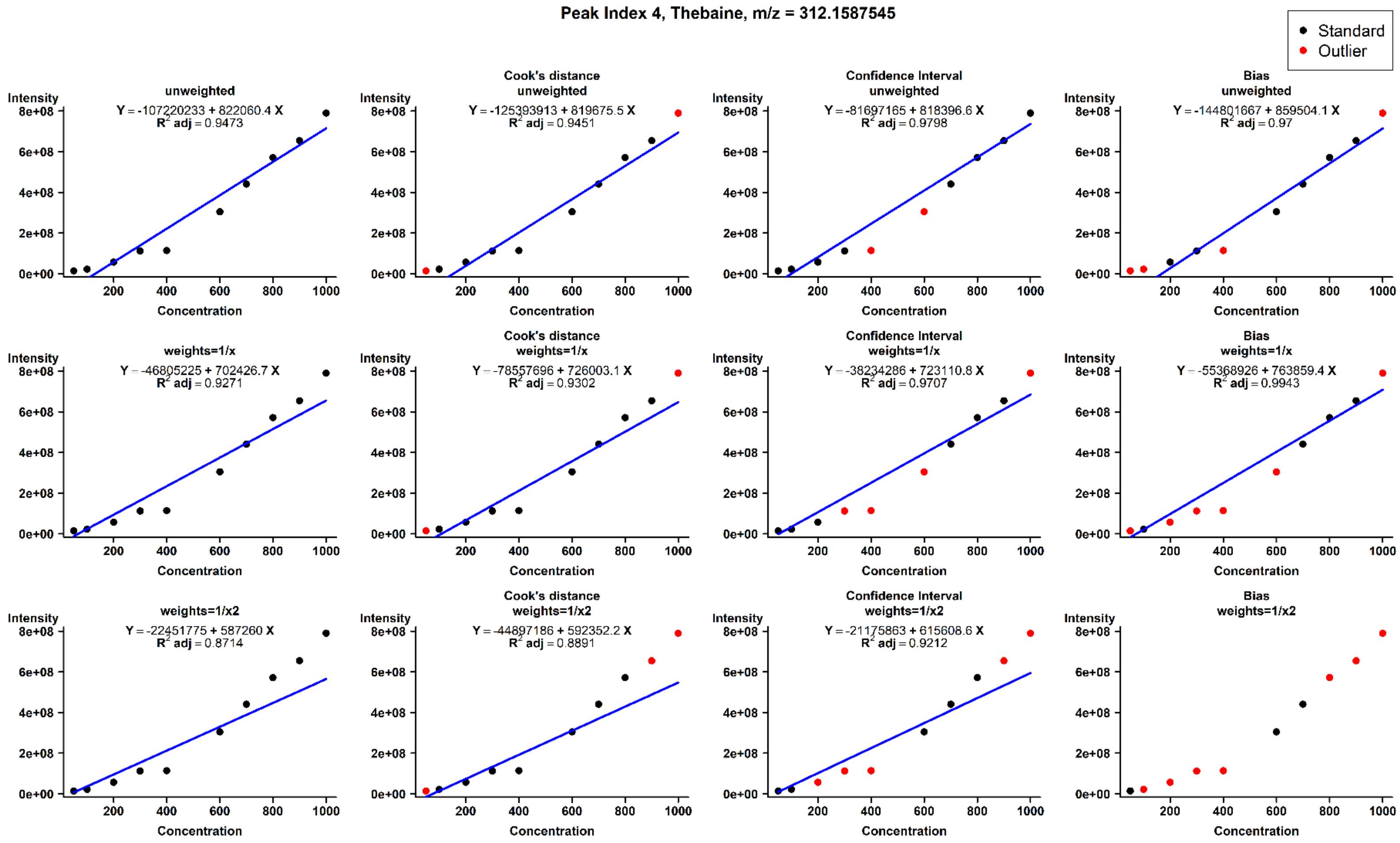

(b)

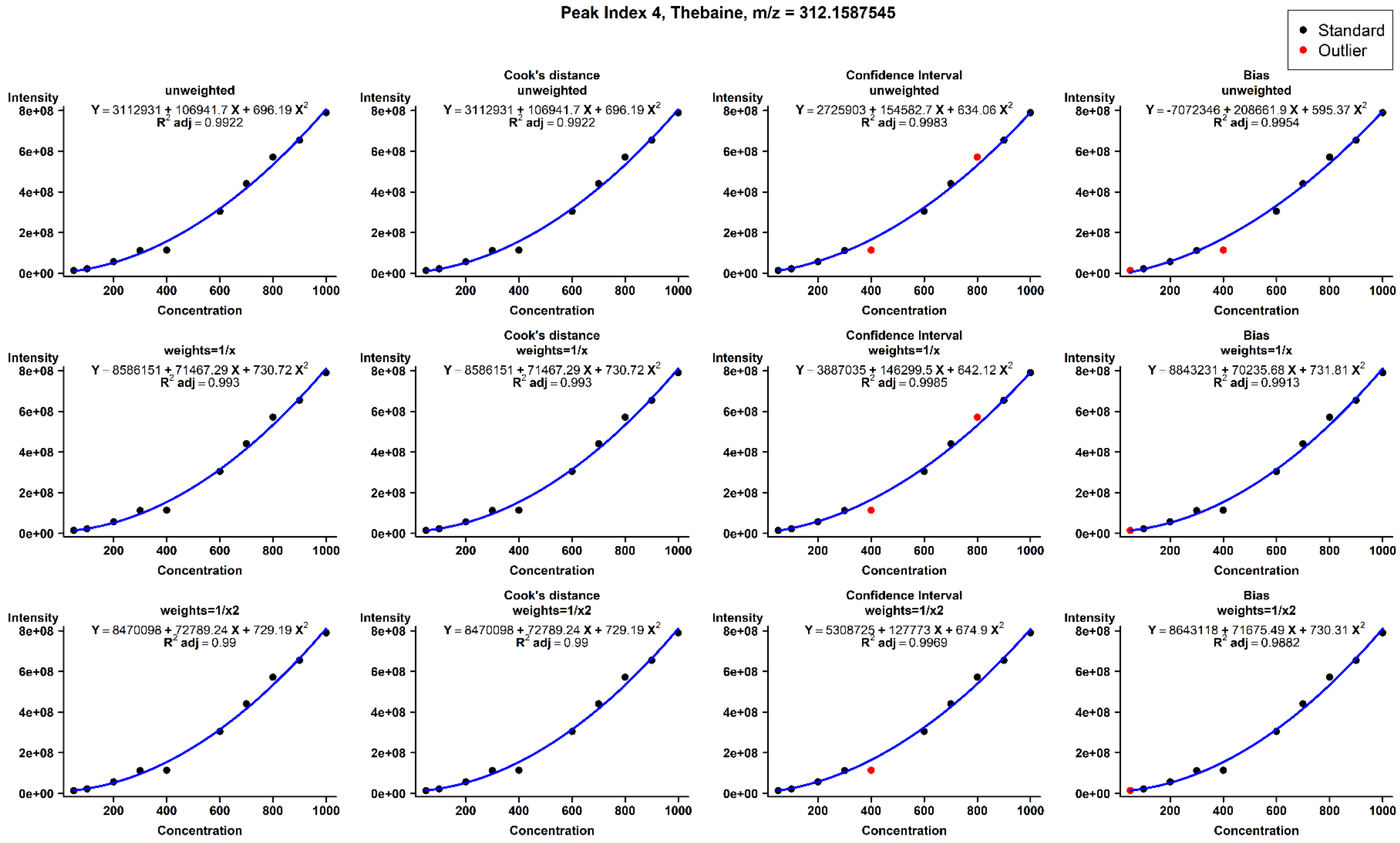
Peak Index 4, Thebaine, m/z = 312.1587545
Standard
Outlier
unweighted
Y = 3112931 + 106941.7 X + 696.19 X2
R2 adj = 0.9922
Cook's distance
unweighted
Y = 3112931 + 106941.7 X + 696.19 X2
R2 adj = 0.9922
Confidence Interval
unweighted
Y = 2725903 + 154582.7 X + 634.06 X2
R2 adj = 0.9983
Bias
unweighted
Y = -7072346 + 208661.9 X + 595.37 X2
R2 adj = 0.9954
weights=1/x
Y = 8586151 + 71467.29 X + 730.72 X2
R2 adj = 0.993
Cook's distance
weights=1/x
Y = 8586151 + 71467.29 X + 730.72 X2
R2 adj = 0.993
Confidence Interval
weights=1/x
Y = 3887035 + 146299.5 X + 642.12 X2
R2 adj = 0.9985
Bias
weights=1/x
Y = 8843231 + 70235.68 X + 731.81 X2
R2 adj = 0.9913
weights=1/x2
Y = 8470098 + 72789.24 X + 729.19 X2
R2 adj = 0.99
Cook's distance
weights=1/x2
Y = 8470098 + 72789.24 X + 729.19 X2
R2 adj = 0.99
Confidence Interval
weights=1/x2
Y = 5308725 + 127773 X + 674.9 X2
R2 adj = 0.9969
Bias
weights=1/x2
Y = 8643118 + 71675.49 X + 730.31 X2
R2 adj = 0.9882
Intensity
Concentration

Figure S5D. Calibration curve for delta9-THC. It was constructed using either a linear or quadratic model, with various weightings, including unweighted, 1/x, and 1/x^2. Additionally, different outlier detection methods were employed, including Cook's D, a 95% confidence interval (CI), and a Bias method (with a 20% relative error). (a) Linear model. (b) Quadratic model.

(a)

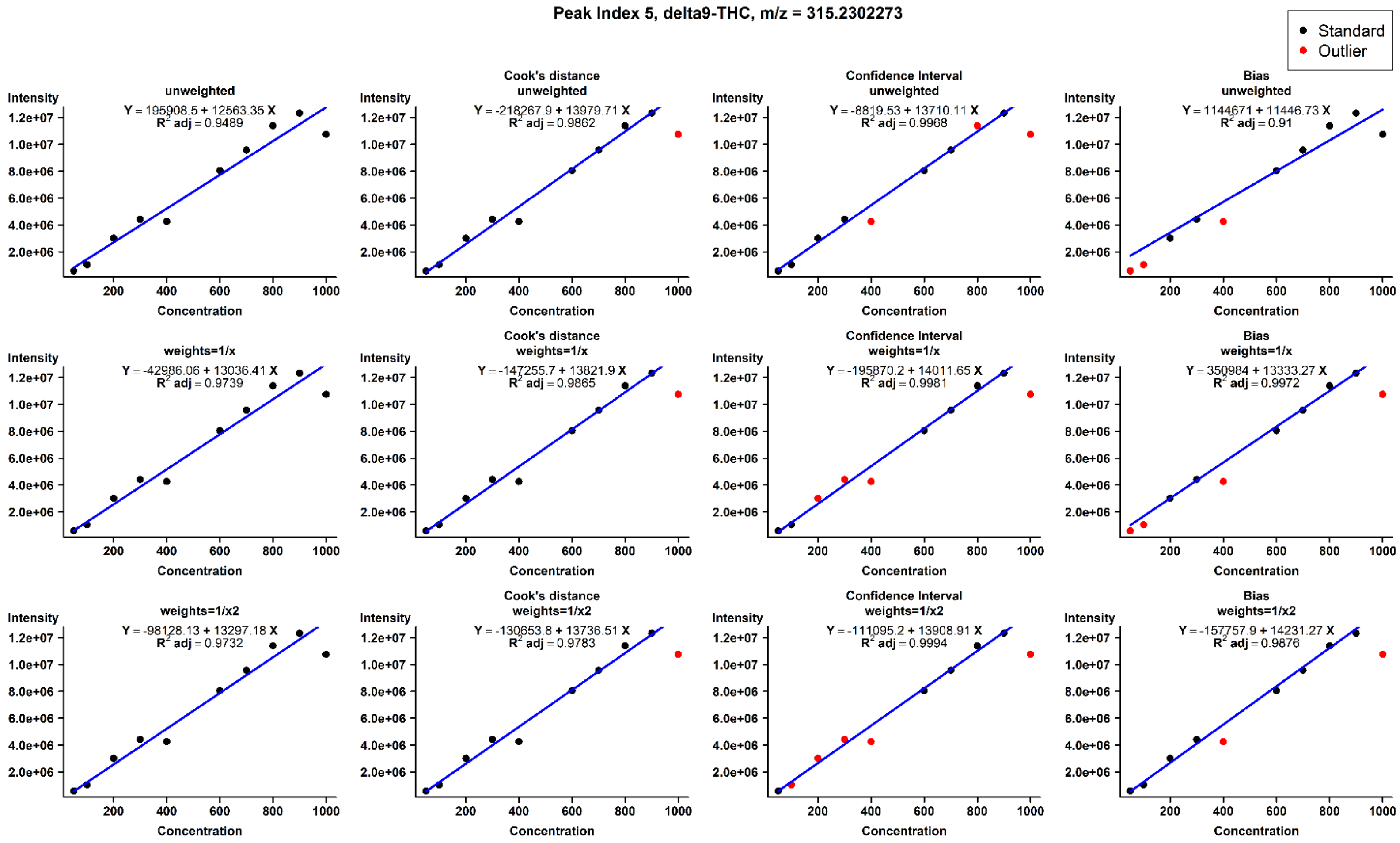

(b)

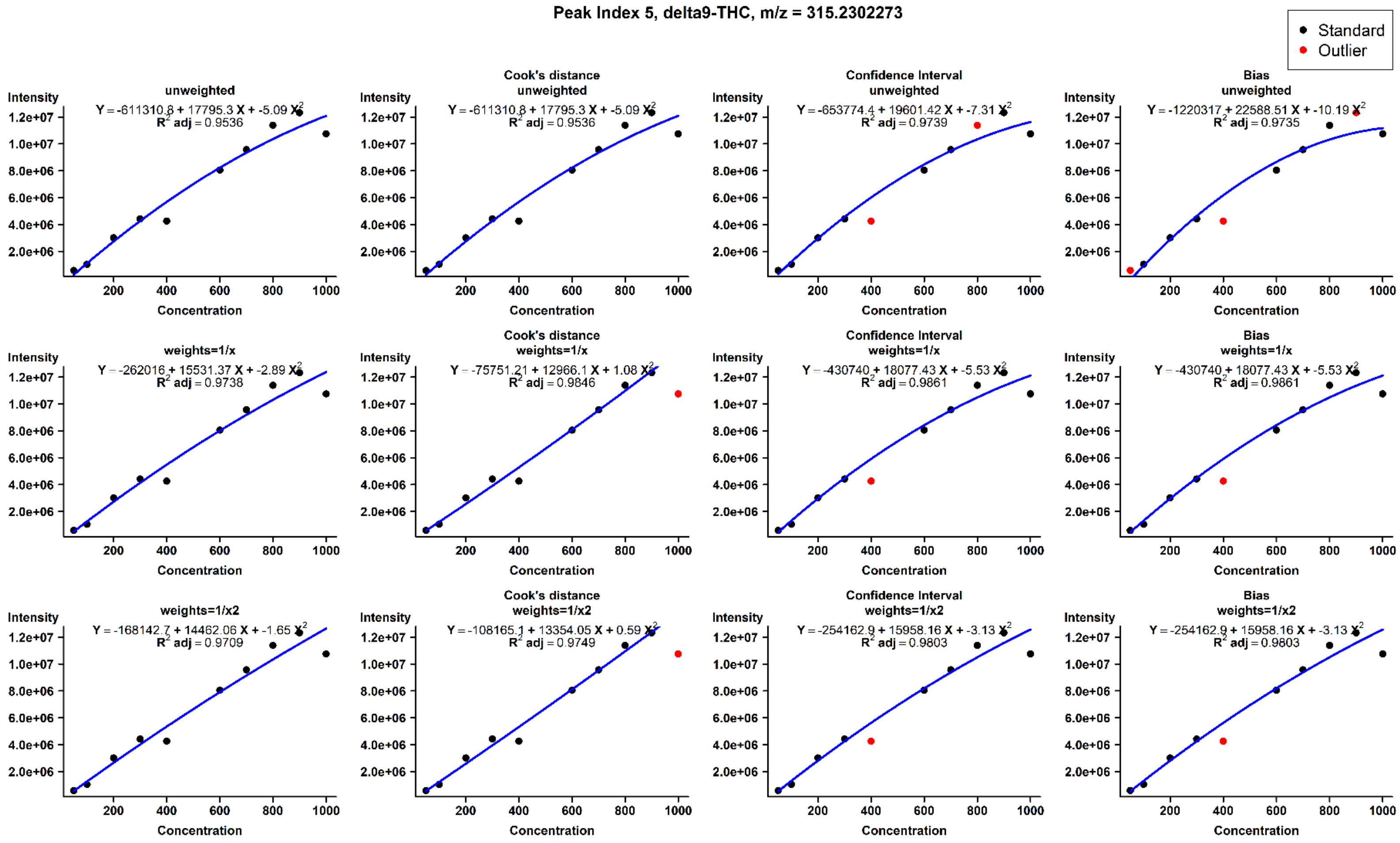

Peak Index 5, delta9-THC, m/z = 315.2302273
Standard
Outlier
unweighted
Y = -611310.8 + 17795.3 X + -5.09 X^2
R^2 adj = 0.9536
Cook's distance
unweighted
Y = -611310.8 + 17795.3 X + -5.09 X^2
R^2 adj = 0.9536
Confidence Interval
unweighted
Y = -653774.4 + 19601.42 X + -7.31 X^2
R^2 adj = 0.9739
Bias
unweighted
Y = -1220317 + 22588.51 X + -10.19 X^2
R^2 adj = 0.9735
weights=1/x
Y = -262016 + 15531.37 X + -2.89 X^2
R^2 adj = 0.9738
Cook's distance
weights=1/x
Y = -75751.21 + 12966.1 X + 1.08 X^2
R^2 adj = 0.9846
Confidence Interval
weights=1/x
Y = -430740 + 18077.43 X + -5.53 X^2
R^2 adj = 0.9861
Bias
weights=1/x
Y = -430740 + 18077.43 X + -5.53 X^2
R^2 adj = 0.9861
weights=1/x2
Y = -168142.7 + 14462.06 X + -1.65 X^2
R^2 adj = 0.9709
Cook's distance
weights=1/x2
Y = -108165.1 + 13354.05 X + 0.59 X^2
R^2 adj = 0.9749
Confidence Interval
weights=1/x2
Y = -254162.9 + 15958.16 X + -3.13 X^2
R^2 adj = 0.9803
Bias
weights=1/x2
Y = -254162.9 + 15958.16 X + -3.13 X^2
R^2 adj = 0.9803
Intensity
1.2e+07
1.0e+07
8.0e+06
6.0e+06
4.0e+06
2.0e+06
Concentration
200
400
600
800
1000

Figure S5E. Calibration curve for amphetamine. It was constructed using either a linear or quadratic model, with various weightings, including unweighted, 1/x, and 1/x^2. Additionally, different outlier detection methods were employed, including Cook's D, a 95% confidence interval (CI), and a Bias method (with a 20% relative error). (a) Linear model. (b) Quadratic model.

(a)

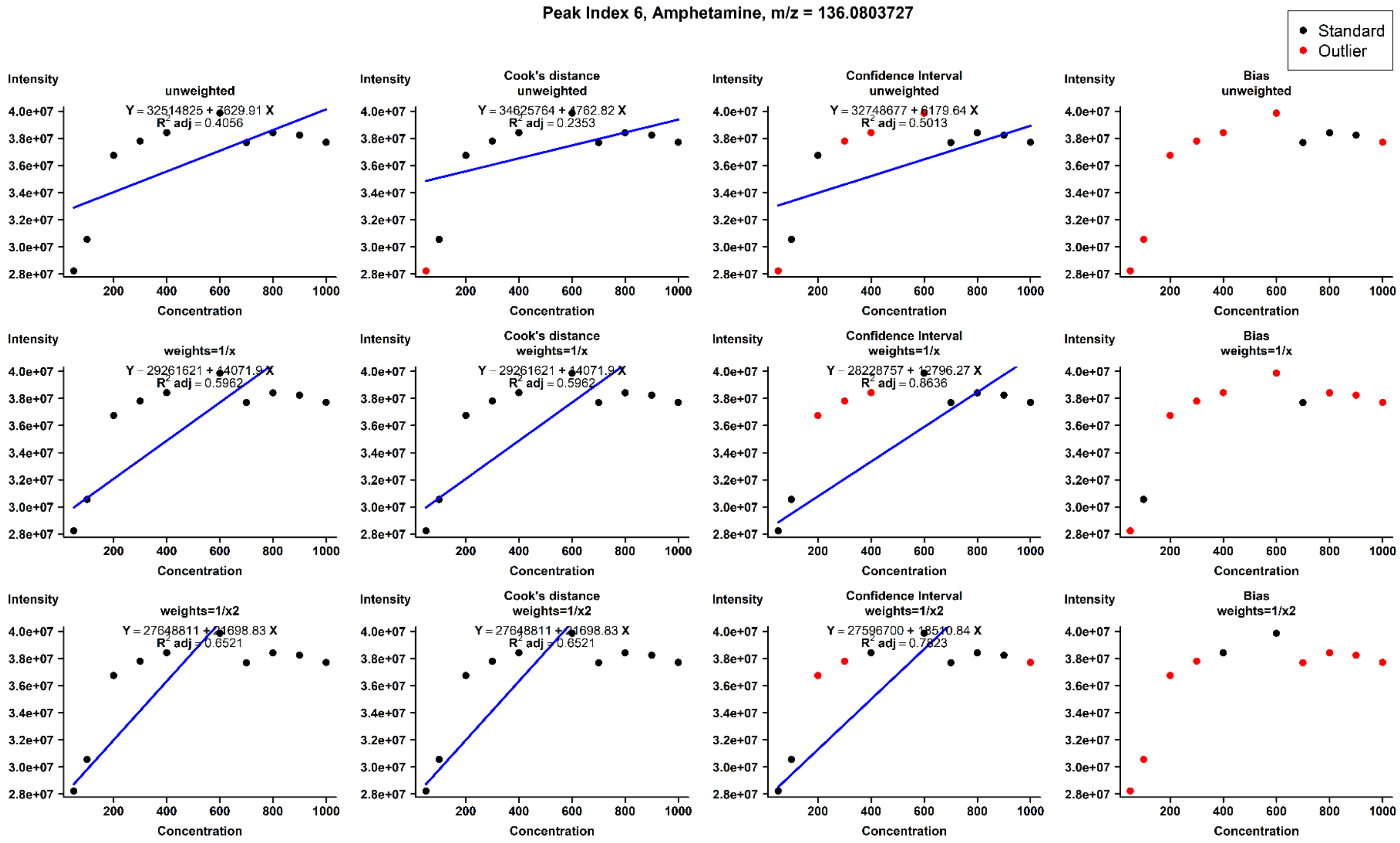

(b)

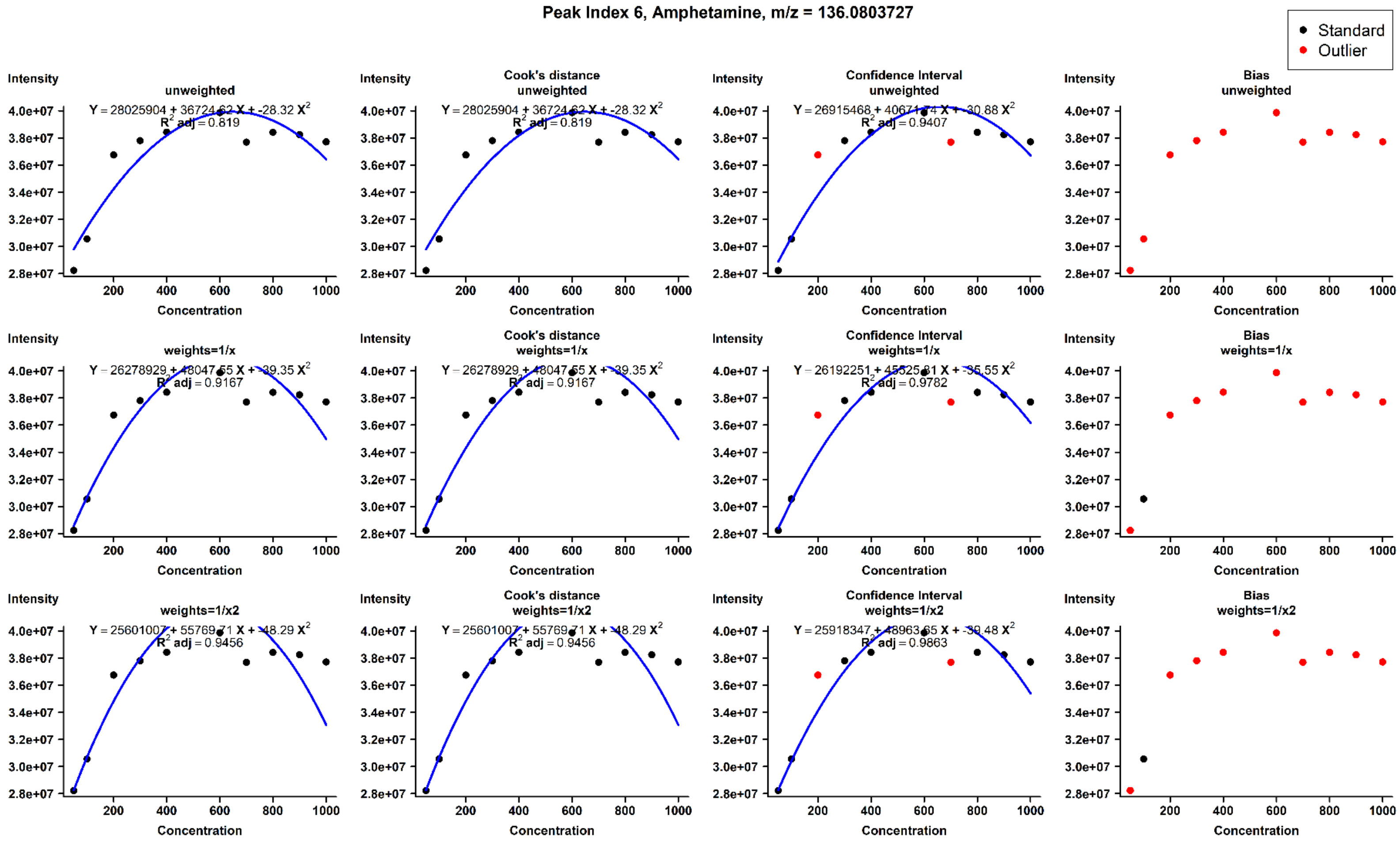

Peak Index 6, Amphetamine, m/z = 136.0803727
Standard
Outlier
Intensity
unweighted
Y = 28025904 + 36724.62 X + -28.32 X2
R2 adj = 0.819
Cook's distance
unweighted
Y = 28025904 + 36724.62 X + -28.32 X2
R2 adj = 0.819
Confidence Interval
unweighted
Y = 26915468 + 40671.74 X + -30.88 X2
R2 adj = 0.9407
Bias
unweighted
weights=1/x
Y = 26278929 + 48047.55 X + -39.35 X2
R2 adj = 0.9167
Cook's distance
weights=1/x
Y = 26278929 + 48047.55 X + -39.35 X2
R2 adj = 0.9167
Confidence Interval
weights=1/x
Y = 26192251 + 45525.81 X + -35.55 X2
R2 adj = 0.9782
Bias
weights=1/x
weights=1/x2
Y = 25601007 + 55769.71 X + -48.29 X2
R2 adj = 0.9456
Cook's distance
weights=1/x2
Y = 25601007 + 55769.71 X + -48.29 X2
R2 adj = 0.9456
Confidence Interval
weights=1/x2
Y = 25918347 + 48963.65 X + -39.48 X2
R2 adj = 0.9863
Bias
weights=1/x2
4.0e+07
3.8e+07
3.6e+07
3.4e+07
3.2e+07
3.0e+07
2.8e+07
200
400
600
800
1000
Concentration

Figure S5F. Calibration curve for MA. It was constructed using either a linear or quadratic model, with various weightings, including unweighted, 1/x, and 1/x^2. Additionally, different outlier detection methods were employed, including Cook's D, a 95% confidence interval (CI), and a Bias method (with a 20% relative error). (a) Linear model. (b) Quadratic model.

(a)

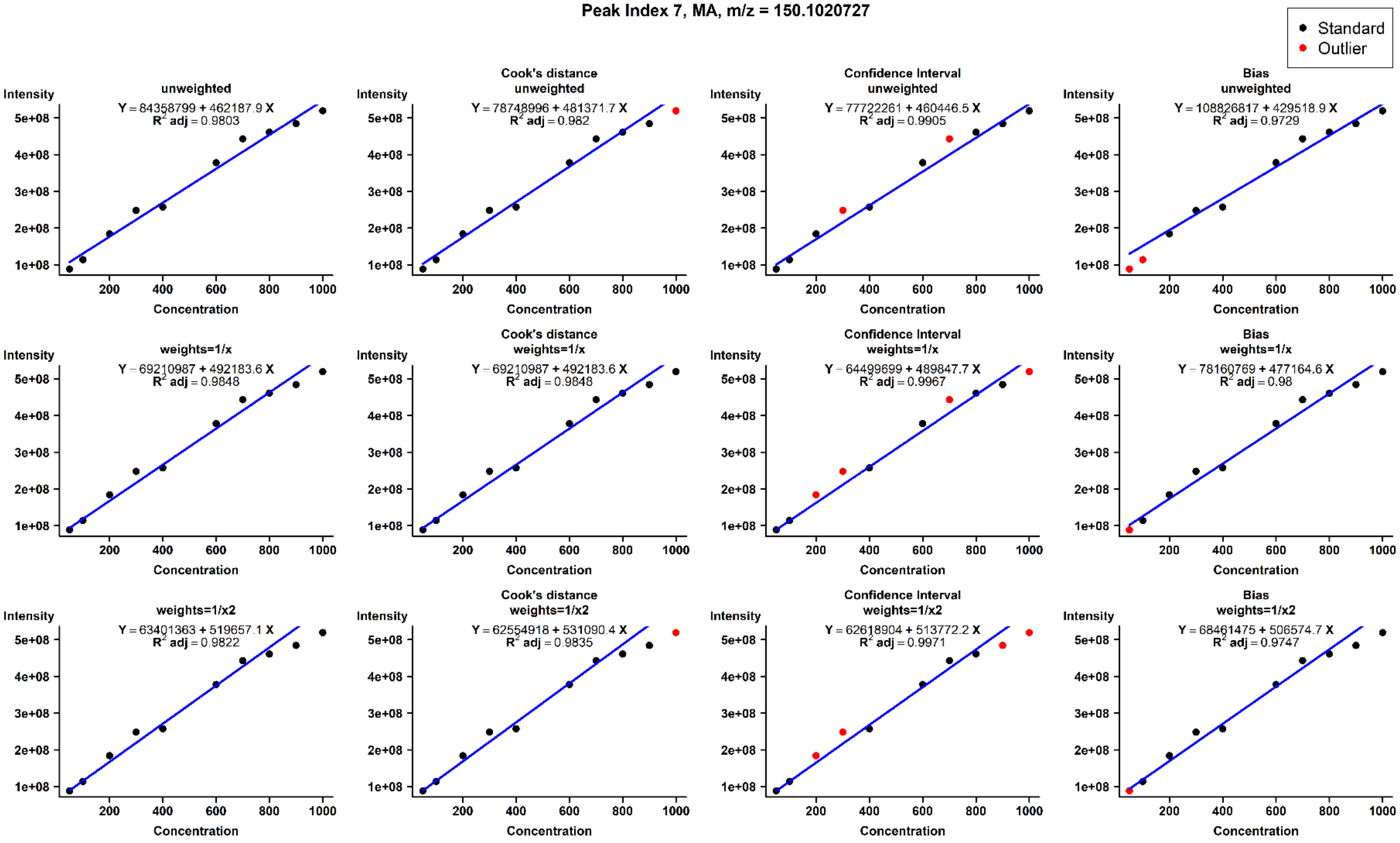

(b)

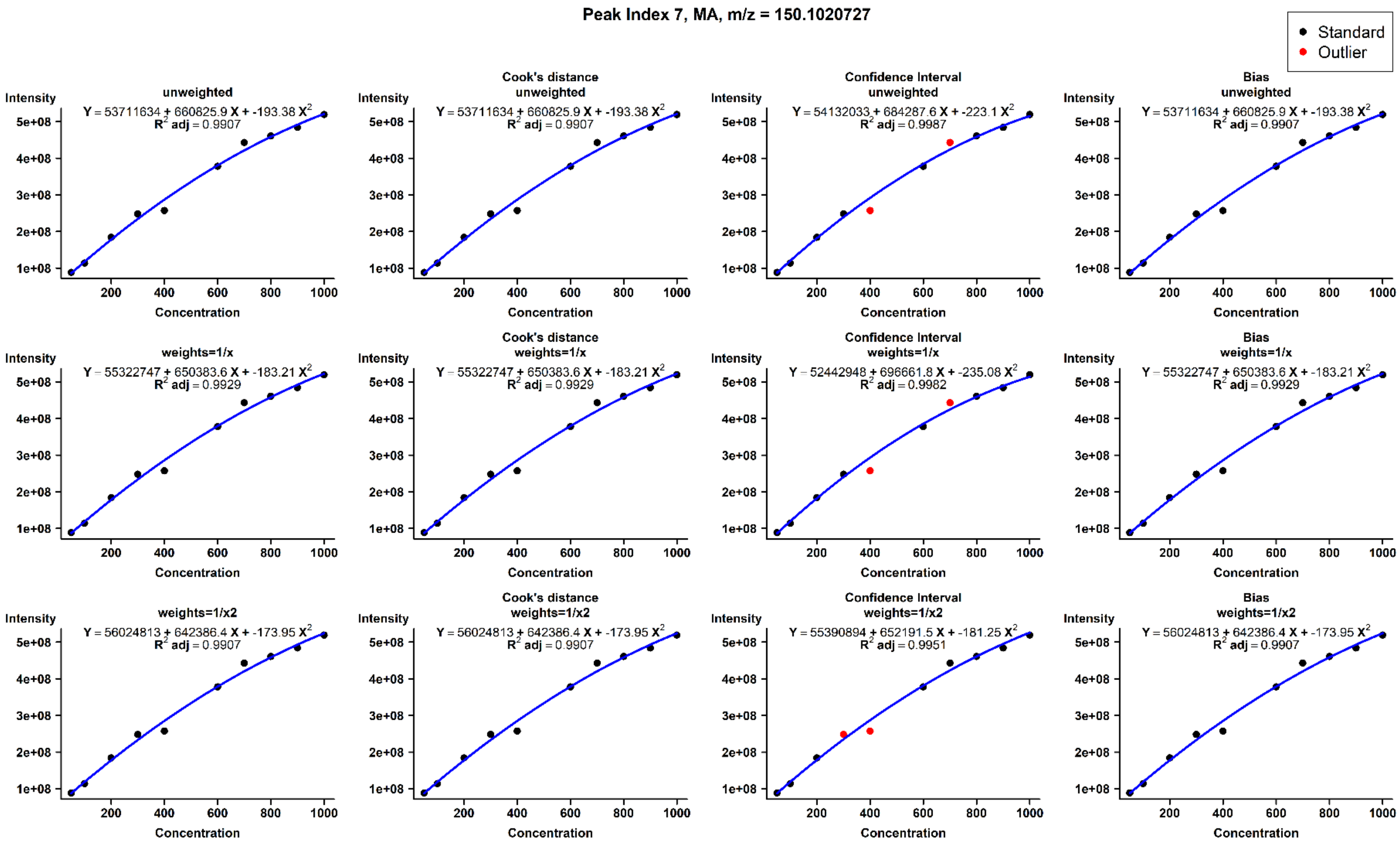

Peak Index 7, MA, m/z = 150.1020727
Standard
Outlier
unweighted
Intensity
Y = 53711634 + 660825.9 X + -193.38 X2
R2 adj = 0.9907
Concentration
Cook's distance
unweighted
Y = 53711634 + 660825.9 X + -193.38 X2
R2 adj = 0.9907
Confidence Interval
unweighted
Y = 54132033 + 684287.6 X + -223.1 X2
R2 adj = 0.9987
Bias
unweighted
Y = 53711634 + 660825.9 X + -193.38 X2
R2 adj = 0.9907
weights=1/x
Y = 55322747 + 650383.6 X + -183.21 X2
R2 adj = 0.9929
Cook's distance
weights=1/x
Y = 55322747 + 650383.6 X + -183.21 X2
R2 adj = 0.9929
Confidence Interval
weights=1/x
Y = 52442948 + 696661.8 X + -235.08 X2
R2 adj = 0.9982
Bias
weights=1/x
Y = 55322747 + 650383.6 X + -183.21 X2
R2 adj = 0.9929
weights=1/x2
Y = 56024813 + 642386.4 X + -173.95 X2
R2 adj = 0.9907
Cook's distance
weights=1/x2
Y = 56024813 + 642386.4 X + -173.95 X2
R2 adj = 0.9907
Confidence Interval
weights=1/x2
Y = 55390894 + 652191.5 X + -181.25 X2
R2 adj = 0.9951
Bias
weights=1/x2
Y = 56024813 + 642386.4 X + -173.95 X2
R2 adj = 0.9907
1e+08
2e+08
3e+08
4e+08
5e+08
200
400
600
800
1000

Figure S5G. Calibration curve for MDMA. It was constructed using either a linear or quadratic model, with various weightings, including unweighted, 1/x, and 1/x^2. Additionally, different outlier detection methods were employed, including Cook's D, a 95% confidence interval (CI), and a Bias method (with a 20% relative error). (a) Linear model. (b) Quadratic model.

(a)

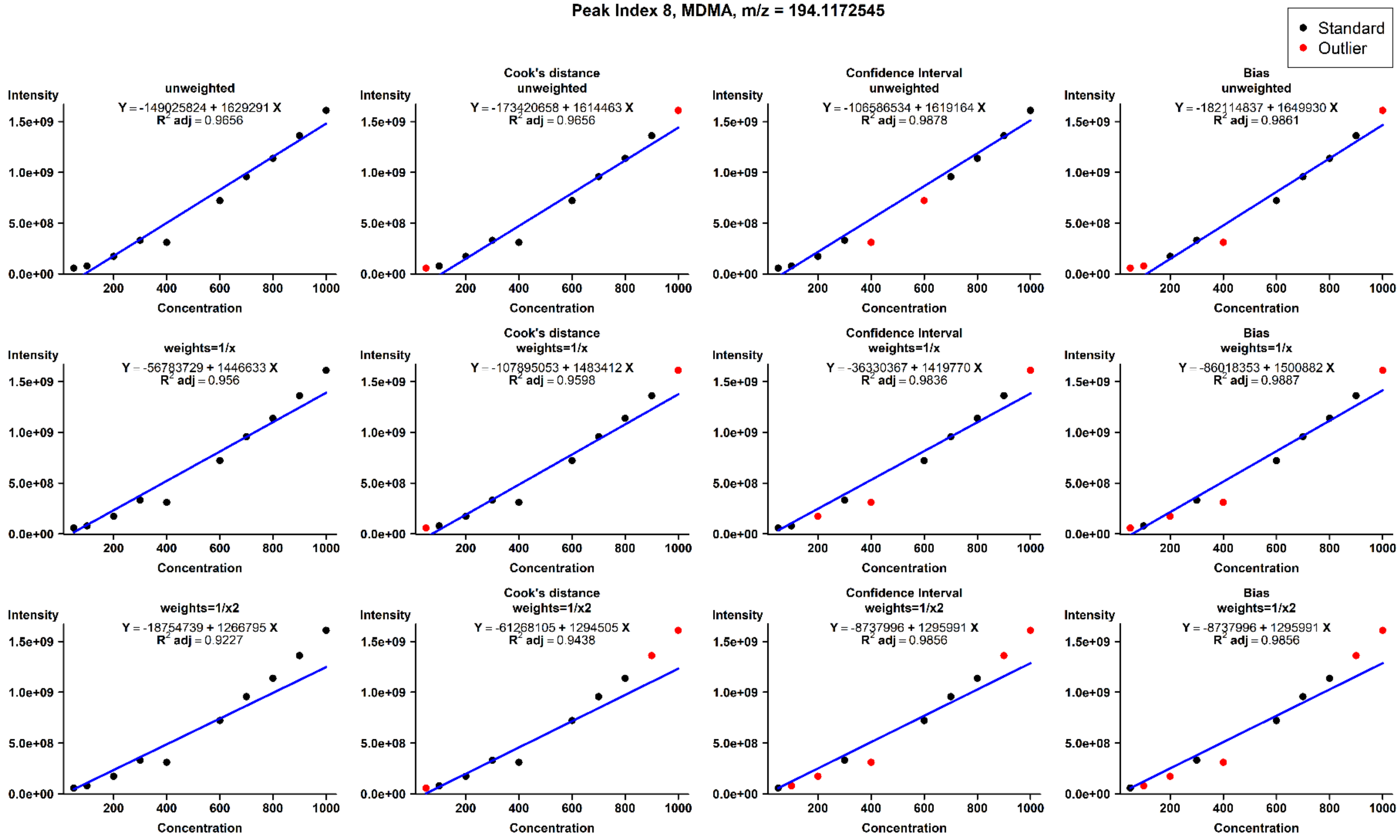

(b)

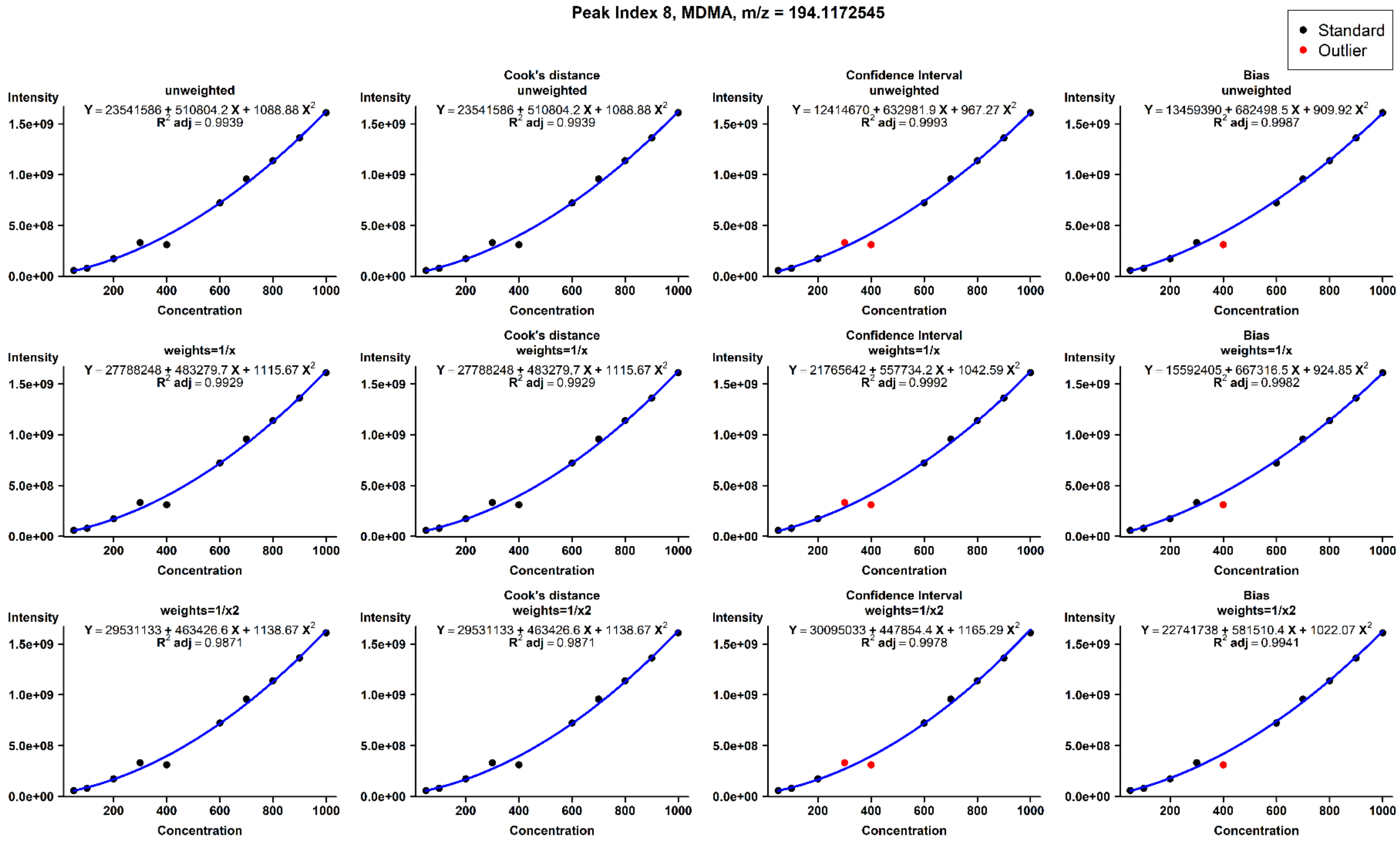
Peak Index 8, MDMA, m/z = 194.1172545
Standard
Outlier
unweighted
Y = 23541586 + 510804.2 X + 1088.88 X2
R2 adj = 0.9939
Cook's distance
unweighted
Y = 23541586 + 510804.2 X + 1088.88 X2
R2 adj = 0.9939
Confidence Interval
unweighted
Y = 12414670 + 632981.9 X + 967.27 X2
R2 adj = 0.9993
Bias
unweighted
Y = 13459390 + 682498.5 X + 909.92 X2
R2 adj = 0.9987
weights=1/x
Y = 27788248 + 483279.7 X + 1115.67 X2
R2 adj = 0.9929
Cook's distance
weights=1/x
Y = 27788248 + 483279.7 X + 1115.67 X2
R2 adj = 0.9929
Confidence Interval
weights=1/x
Y = 21765642 + 557734.2 X + 1042.59 X2
R2 adj = 0.9992
Bias
weights=1/x
Y = 15592405 + 667316.5 X + 924.85 X2
R2 adj = 0.9982
weights=1/x2
Y = 29531133 + 463426.6 X + 1138.67 X2
R2 adj = 0.9871
Cook's distance
weights=1/x2
Y = 29531133 + 463426.6 X + 1138.67 X2
R2 adj = 0.9871
Confidence Interval
weights=1/x2
Y = 30095033 + 447854.4 X + 1165.29 X2
R2 adj = 0.9978
Bias
weights=1/x2
Y = 22741738 + 581510.4 X + 1022.07 X2
R2 adj = 0.9941
Intensity
1.5e+09
1.0e+09
5.0e+08
0.0e+00
200
400
600
800
1000
Concentration

Figure S5H. Calibration curve for MDA (love drug). It was constructed using either a linear or quadratic model, with various weightings, including unweighted, 1/x, and 1/x^2. Additionally, different outlier detection methods were employed, including Cook's D, a 95% confidence interval (CI), and a Bias method (with a 20% relative error). (a) Linear model. (b) Quadratic model.

(a)

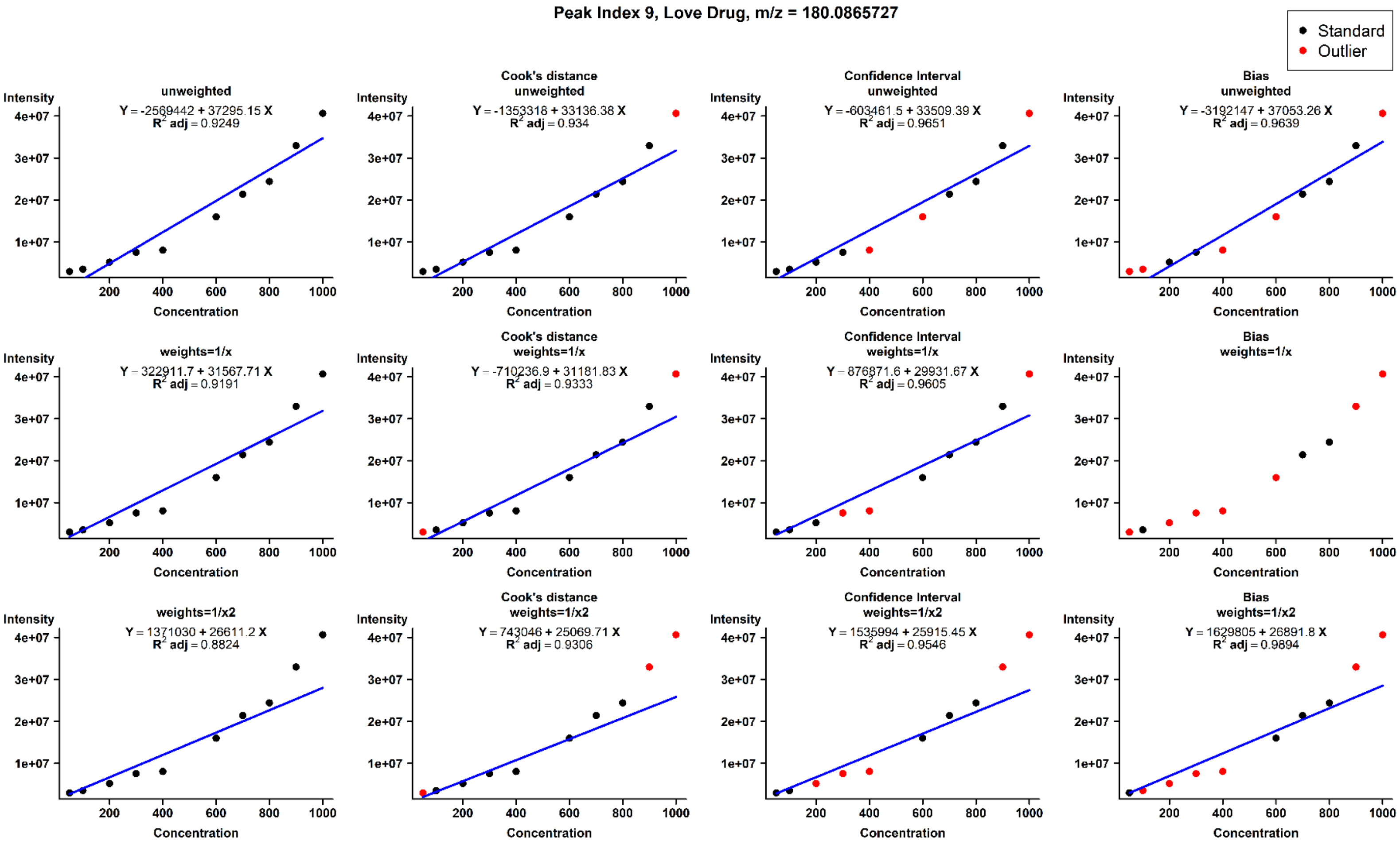

(b)

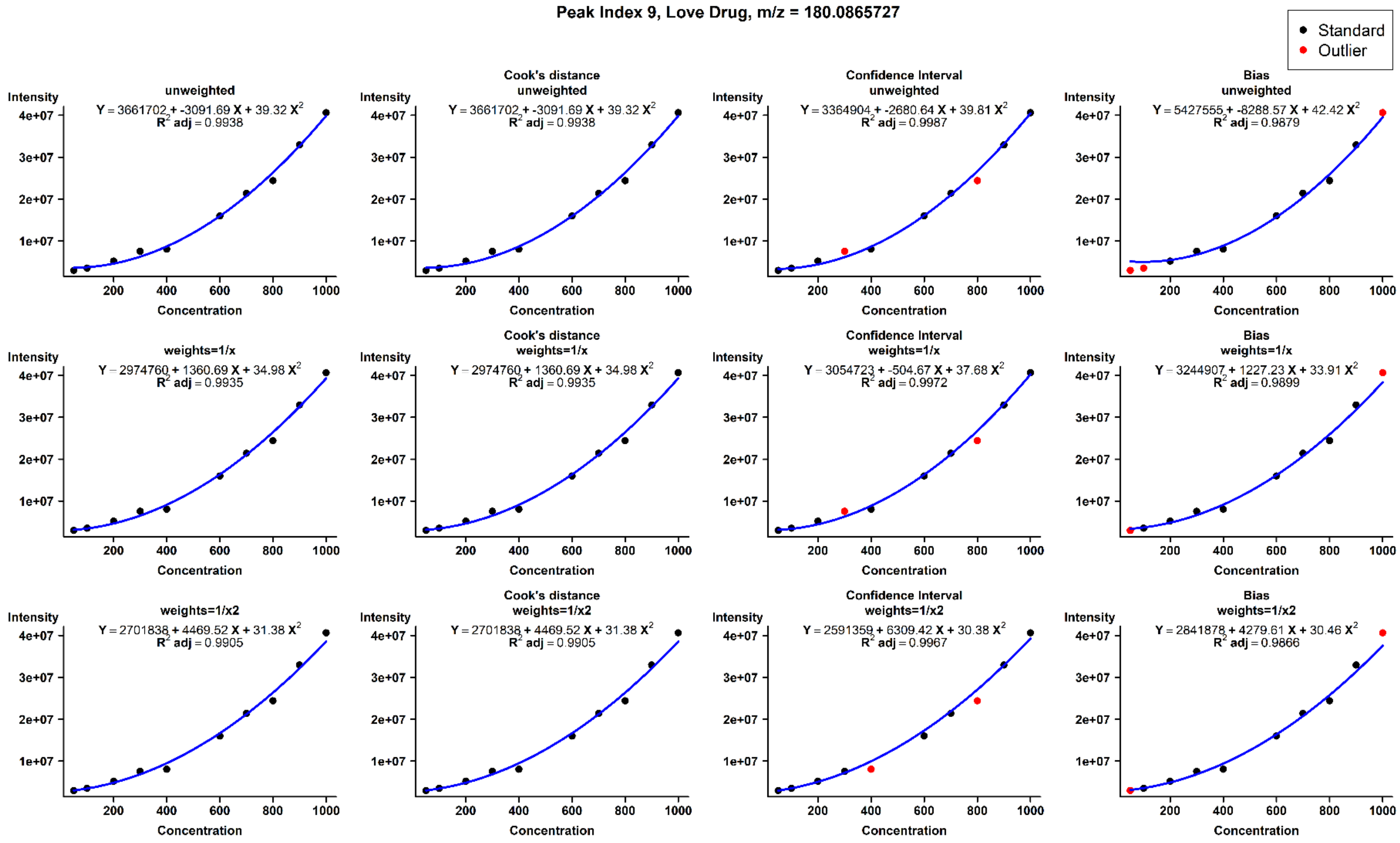

Peak Index 9, Love Drug, m/z = 180.0865727
Standard
Outlier
unweighted
Y = 3661702 + -3091.69 X + 39.32 X2
R2 adj = 0.9938
Cook's distance
unweighted
Y = 3661702 + -3091.69 X + 39.32 X2
R2 adj = 0.9938
Confidence Interval
unweighted
Y = 3364904 + -2680.64 X + 39.81 X2
R2 adj = 0.9987
Bias
unweighted
Y = 5427555 + -8288.57 X + 42.42 X2
R2 adj = 0.9879
weights=1/x
Y = 2974760 + 1360.69 X + 34.98 X2
R2 adj = 0.9935
Cook's distance
weights=1/x
Y = 2974760 + 1360.69 X + 34.98 X2
R2 adj = 0.9935
Confidence Interval
weights=1/x
Y = 3054723 + -504.67 X + 37.68 X2
R2 adj = 0.9972
Bias
weights=1/x
Y = 3244907 + 1227.23 X + 33.91 X2
R2 adj = 0.9899
weights=1/x2
Y = 2701838 + 4469.52 X + 31.38 X2
R2 adj = 0.9905
Cook's distance
weights=1/x2
Y = 2701838 + 4469.52 X + 31.38 X2
R2 adj = 0.9905
Confidence Interval
weights=1/x2
Y = 2591359 + 6309.42 X + 30.38 X2
R2 adj = 0.9967
Bias
weights=1/x2
Y = 2841878 + 4279.61 X + 30.46 X2
R2 adj = 0.9866
Intensity
4e+07
3e+07
2e+07
1e+07
200
400
600
800
1000
Concentration

Figure S5I. Calibration curve for ketamine. It was constructed using either a linear or quadratic model, with various weightings, including unweighted, 1/x, and 1/x^2. Additionally, different outlier detection methods were employed, including Cook's D, a 95% confidence interval (CI), and a Bias method (with a 20% relative error). (a) Linear model. (b) Quadratic model.

(a)

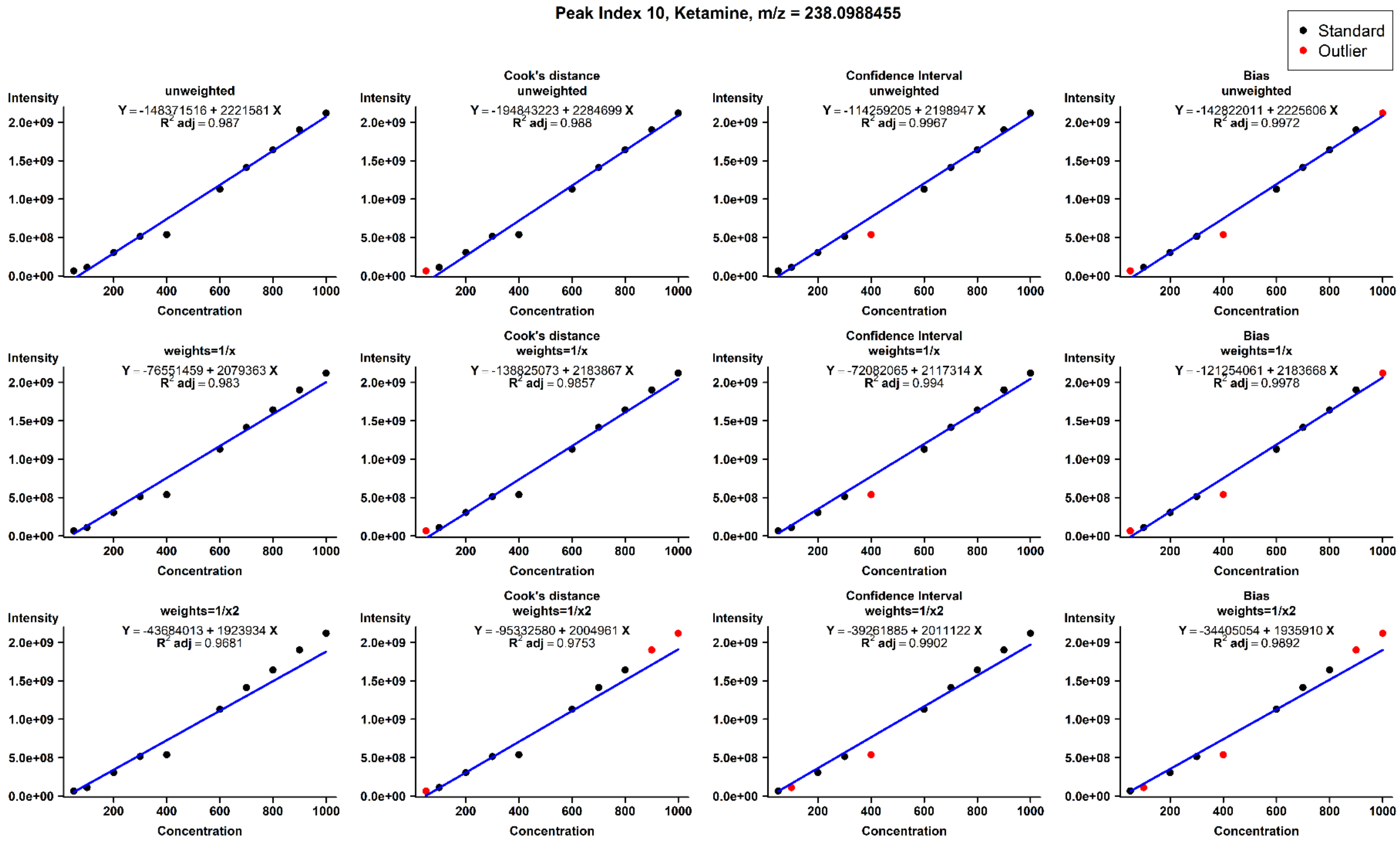

(b)

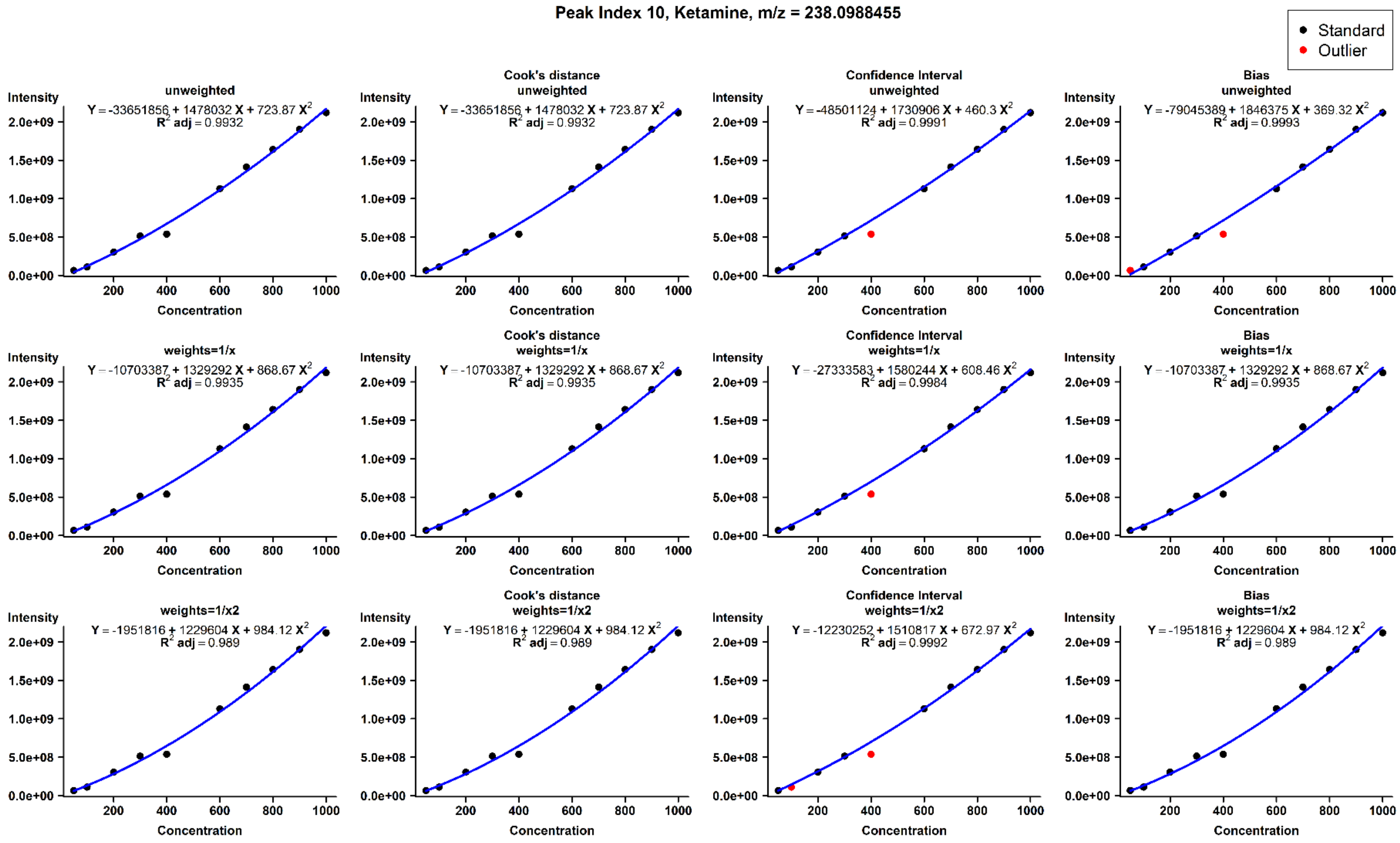

Peak Index 10, Ketamine, m/z = 238.0988455
Standard
Outlier
Intensity
Concentration
unweighted
Y = -33651856 + 1478032 X + 723.87 X2
R2 adj = 0.9932
Cook's distance
unweighted
Y = -33651856 + 1478032 X + 723.87 X2
R2 adj = 0.9932
Confidence Interval
unweighted
Y = -48501124 + 1730906 X + 460.3 X2
R2 adj = 0.9991
Bias
unweighted
Y = -79045389 + 1846375 X + 369.32 X2
R2 adj = 0.9993
weights=1/x
Y = -10703387 + 1329292 X + 868.67 X2
R2 adj = 0.9935
Cook's distance
weights=1/x
Y = -10703387 + 1329292 X + 868.67 X2
R2 adj = 0.9935
Confidence Interval
weights=1/x
Y = -27333583 + 1580244 X + 608.46 X2
R2 adj = 0.9984
Bias
weights=1/x
Y = -10703387 + 1329292 X + 868.67 X2
R2 adj = 0.9935
weights=1/x2
Y = -1951816 + 1229604 X + 984.12 X2
R2 adj = 0.989
Cook's distance
weights=1/x2
Y = -1951816 + 1229604 X + 984.12 X2
R2 adj = 0.989
Confidence Interval
weights=1/x2
Y = -12230252 + 1510817 X + 672.97 X2
R2 adj = 0.9992
Bias
weights=1/x2
Y = -1951816 + 1229604 X + 984.12 X2
R2 adj = 0.989
0.0e+00
5.0e+08
1.0e+09
1.5e+09
2.0e+09
200
400
600
800
1000

Figure S5J. Calibration curve for FM2. It was constructed using either a linear or quadratic model, with various weightings, including unweighted, 1/x, and 1/x^2. Additionally, different outlier detection methods were employed, including Cook's D, a 95% confidence interval (CI), and a Bias method (with a 20% relative error). (a) Linear model. (b) Quadratic model.

(a)

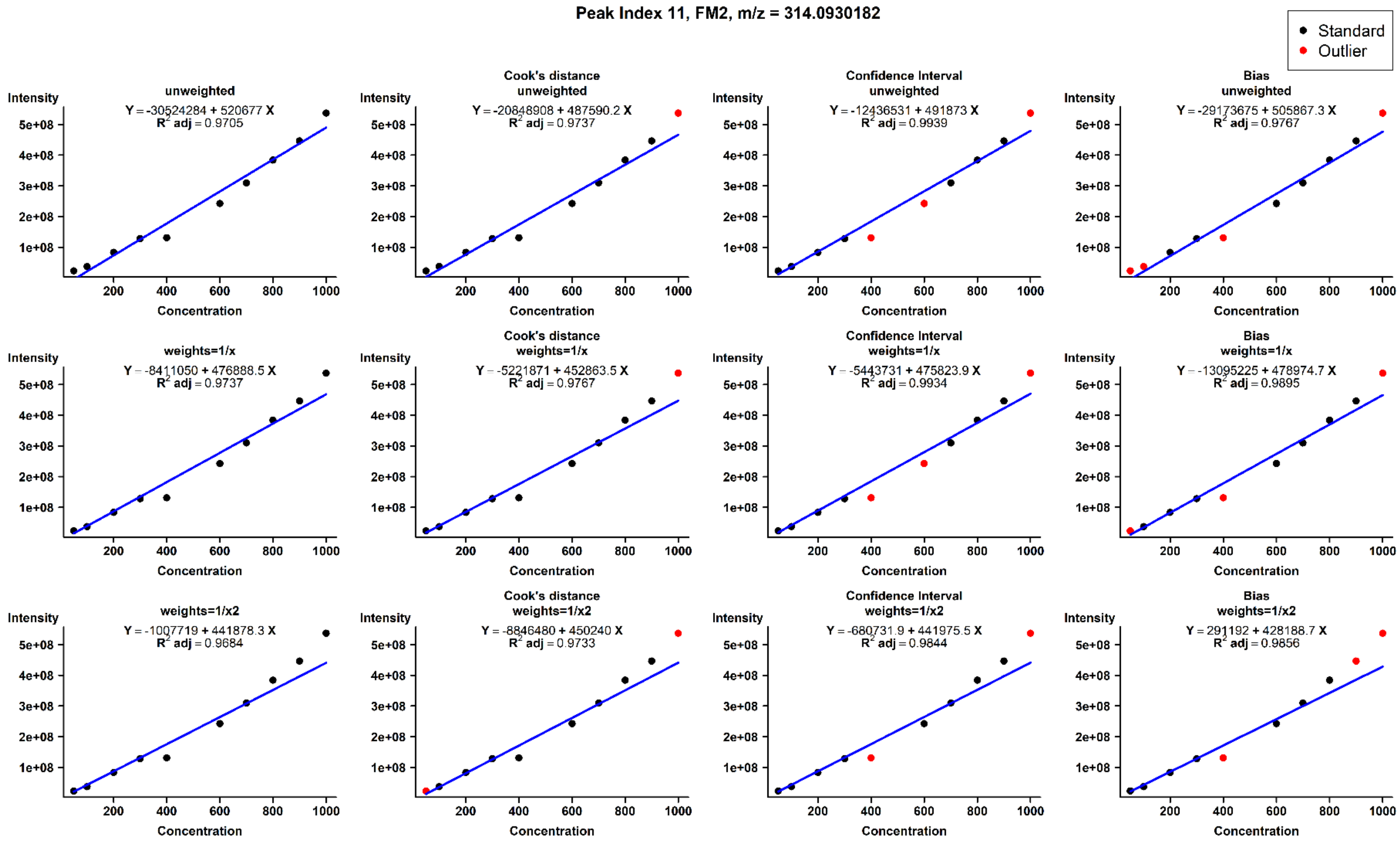

(b)

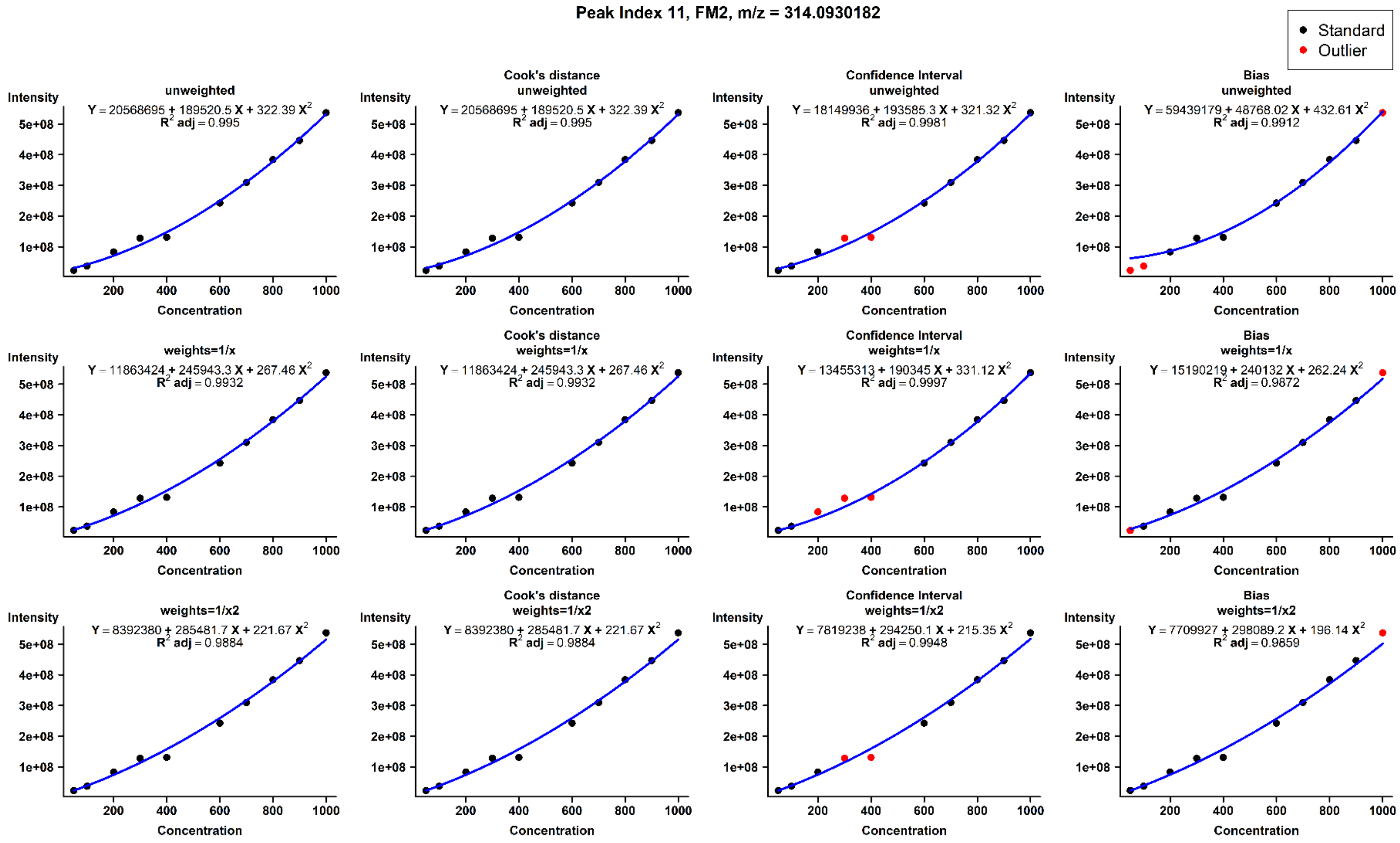
Peak Index 11, FM2, m/z = 314.0930182
Standard
Outlier
unweighted
Y = 20568695 + 189520.5 X + 322.39 X2
R2 adj = 0.995
Cook's distance
unweighted
Y = 20568695 + 189520.5 X + 322.39 X2
R2 adj = 0.995
Confidence Interval
unweighted
Y = 18149936 + 193585.3 X + 321.32 X2
R2 adj = 0.9981
Bias
unweighted
Y = 59439179 + 48768.02 X + 432.61 X2
R2 adj = 0.9912
weights=1/x
Y = 11863424 + 245943.3 X + 267.46 X2
R2 adj = 0.9932
Cook's distance
weights=1/x
Y = 11863424 + 245943.3 X + 267.46 X2
R2 adj = 0.9932
Confidence Interval
weights=1/x
Y = 13455313 + 190345 X + 331.12 X2
R2 adj = 0.9997
Bias
weights=1/x
Y = 15190219 + 240132 X + 262.24 X2
R2 adj = 0.9872
weights=1/x2
Y = 8392380 + 285481.7 X + 221.67 X2
R2 adj = 0.9884
Cook's distance
weights=1/x2
Y = 8392380 + 285481.7 X + 221.67 X2
R2 adj = 0.9884
Confidence Interval
weights=1/x2
Y = 7819238 + 294250.1 X + 215.35 X2
R2 adj = 0.9948
Bias
weights=1/x2
Y = 7709927 + 298089.2 X + 196.14 X2
R2 adj = 0.9859
Intensity
1e+08
2e+08
3e+08
4e+08
5e+08
200
400
600
800
1000
Concentration

Figure S5K. Calibration curve for nimetazepam. It was constructed using either a linear or quadratic model, with various weightings, including unweighted, 1/x, and 1/x^2. Additionally, different outlier detection methods were employed, including Cook's D, a 95% confidence interval (CI), and a Bias method (with a 20% relative error). (a) Linear model. (b) Quadratic model.

(a)

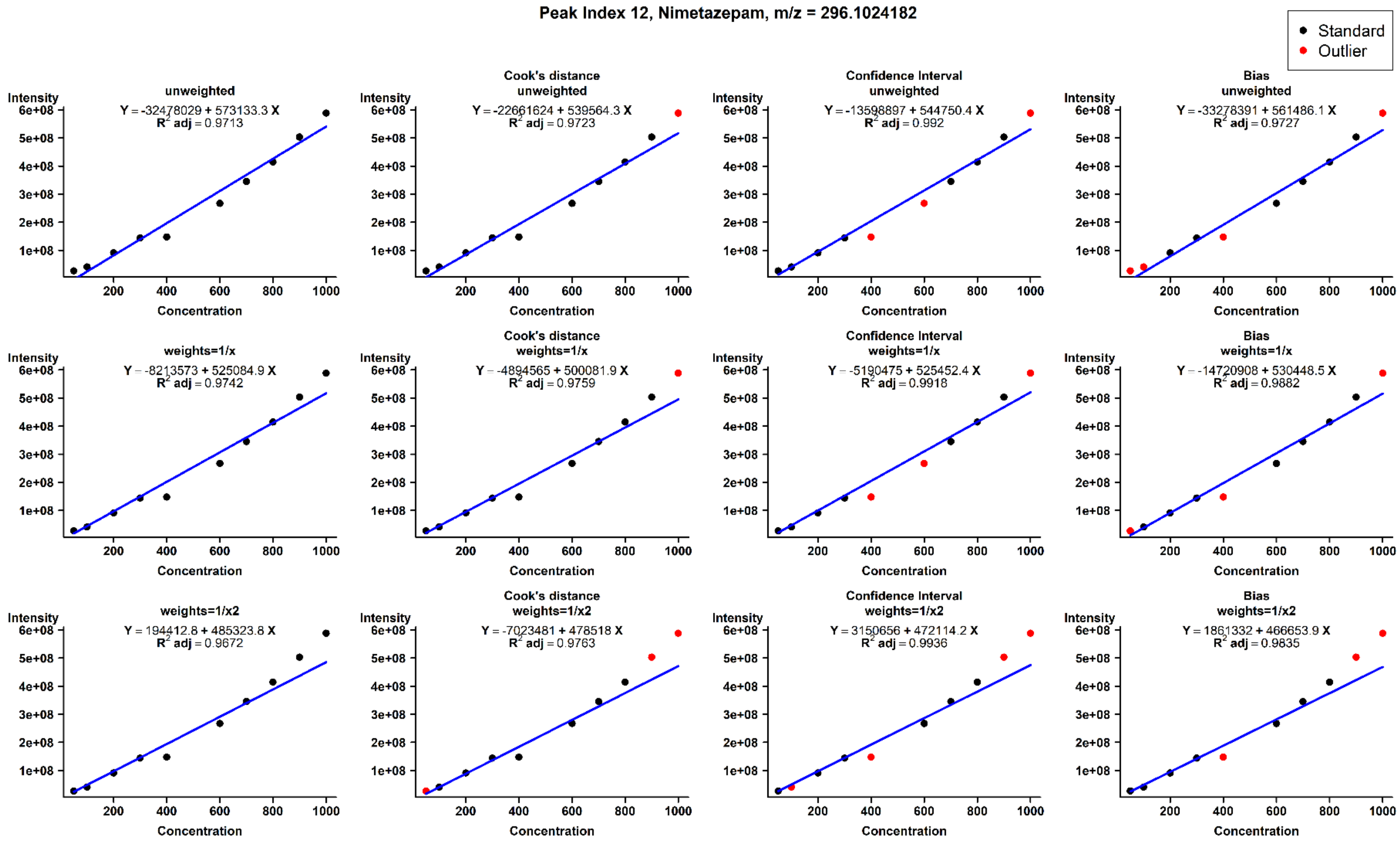

(b)

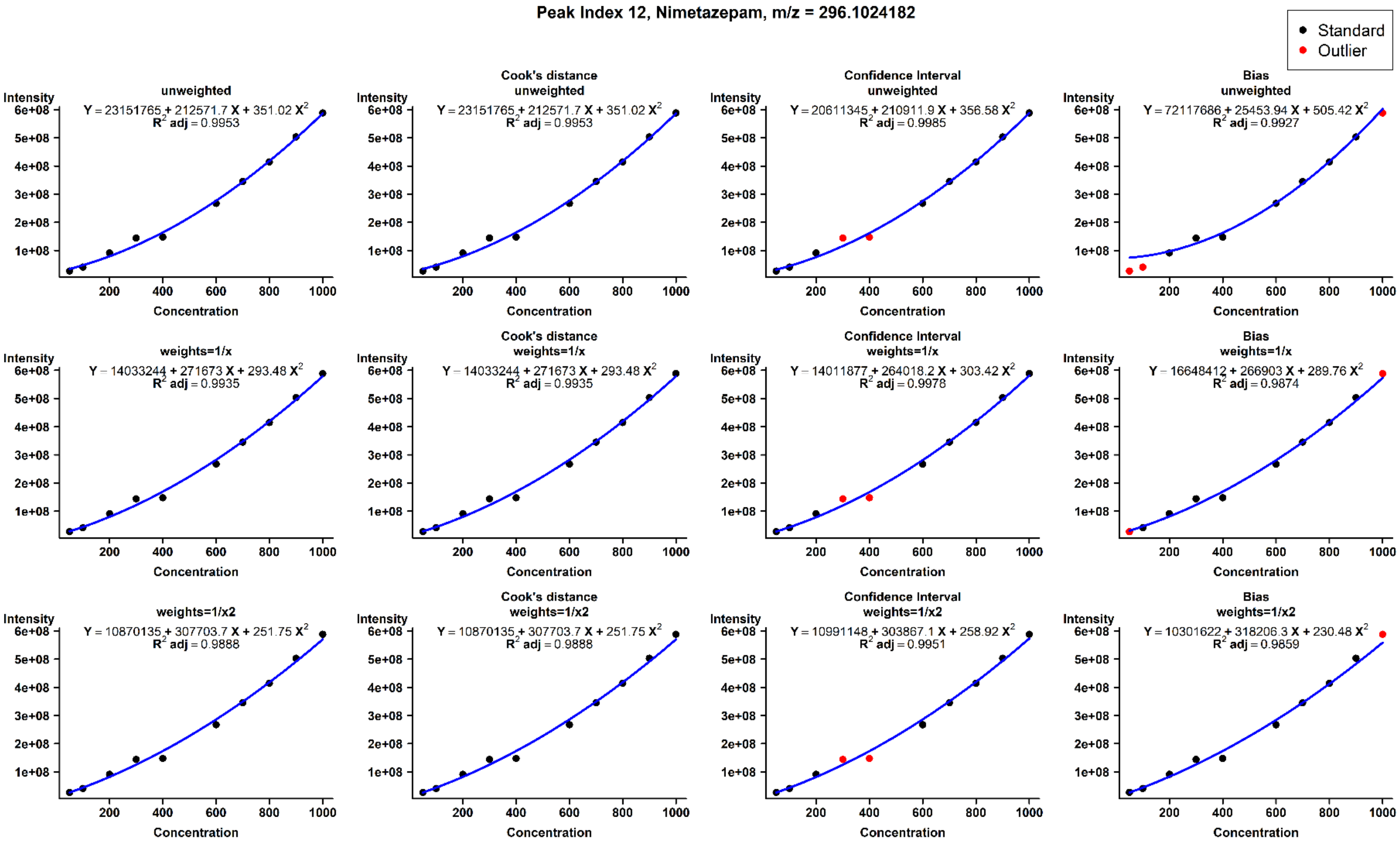

Peak Index 12, Nimetazepam, m/z = 296.1024182
Standard
Outlier
unweighted
Y = 23151765 + 212571.7 X + 351.02 X2
R2 adj = 0.9953
Cook's distance
unweighted
Y = 23151765 + 212571.7 X + 351.02 X2
R2 adj = 0.9953
Confidence Interval
unweighted
Y = 20611345 + 210911.9 X + 356.58 X2
R2 adj = 0.9985
Bias
unweighted
Y = 72117686 + 25453.94 X + 505.42 X2
R2 adj = 0.9927
weights=1/x
Y = 14033244 + 271673 X + 293.48 X2
R2 adj = 0.9935
Cook's distance
weights=1/x
Y = 14033244 + 271673 X + 293.48 X2
R2 adj = 0.9935
Confidence Interval
weights=1/x
Y = 14011877 + 264018.2 X + 303.42 X2
R2 adj = 0.9978
Bias
weights=1/x
Y = 16648412 + 266903 X + 289.76 X2
R2 adj = 0.9874
weights=1/x2
Y = 10870135 + 307703.7 X + 251.75 X2
R2 adj = 0.9888
Cook's distance
weights=1/x2
Y = 10870135 + 307703.7 X + 251.75 X2
R2 adj = 0.9888
Confidence Interval
weights=1/x2
Y = 10991148 + 303867.1 X + 258.92 X2
R2 adj = 0.9951
Bias
weights=1/x2
Y = 10301622 + 318206.3 X + 230.48 X2
R2 adj = 0.9659
Intensity
6e+08
5e+08
4e+08
3e+08
2e+08
1e+08
200
400
600
800
1000
Concentration

Figure S6A. Optimal calibrated concentration plot for morphine. The graph illustrates the calculated concentrations of morphine in the 500 ppb test sample, utilizing the optimal linear calibration curve with the weighted method as x and applying the bias method to detect outliers. Blue denotes the 500 ppb test sample, while black indicates the other standards.

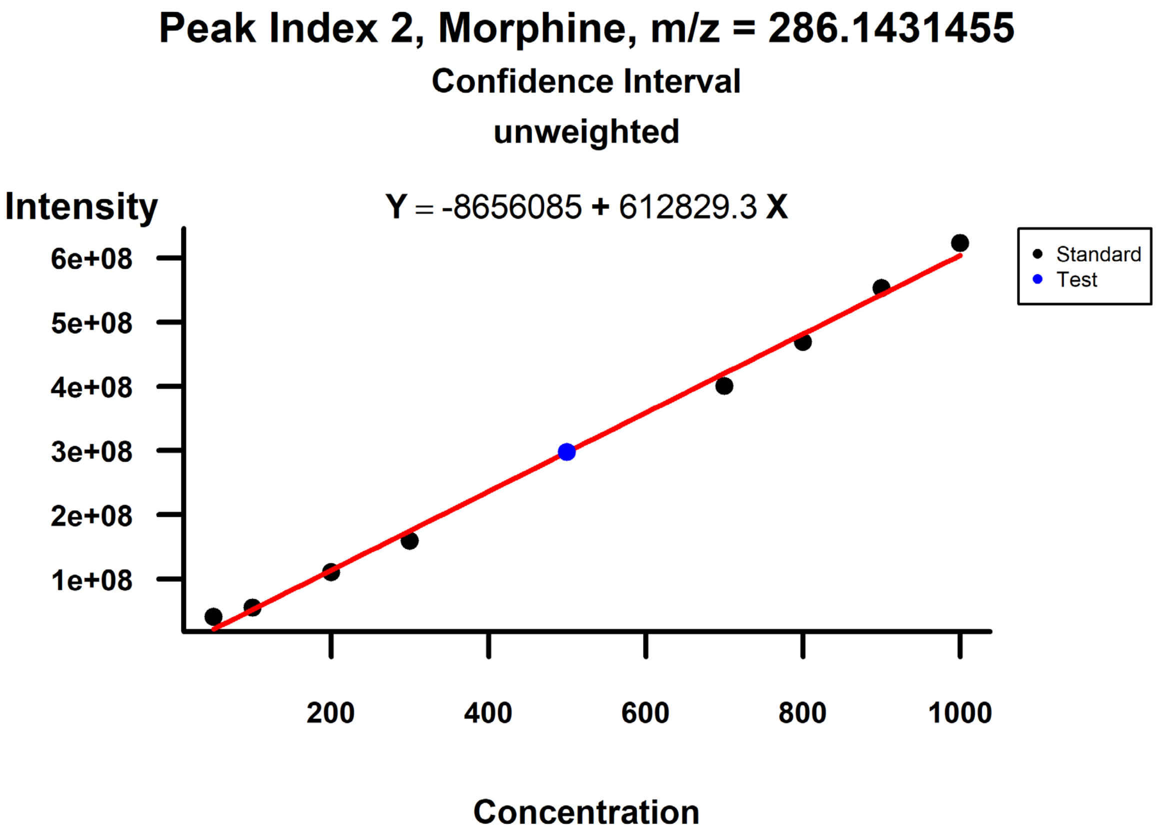

Figure S6B. Optimal calibrated concentration plot for cocaine. The graph illustrates the calculated concentrations of cocaine in the 500 ppb test sample, utilizing the optimal linear calibration curve with the weighted method as x and applying the bias method to detect outliers. Blue denotes the 500 ppb test sample, while black indicates the other standards.

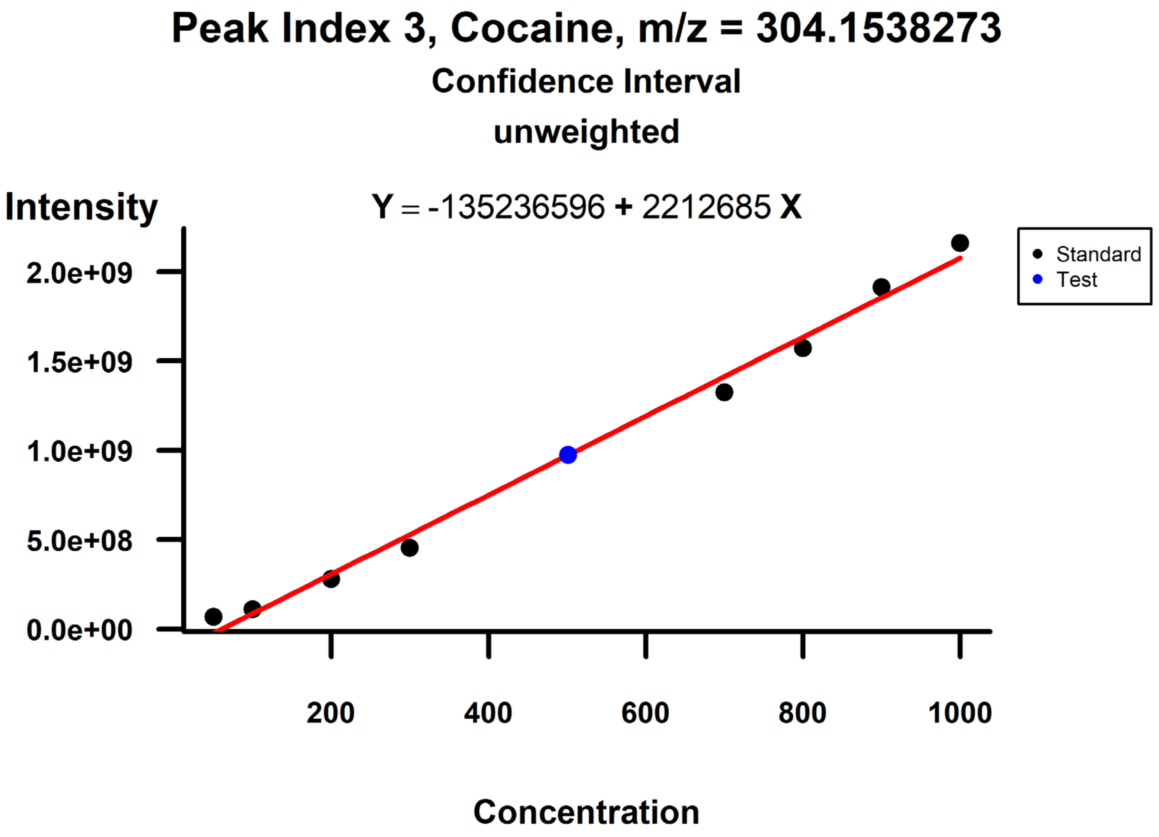

Figure S6C. Optimal calibrated concentration plot for thebaine. The graph illustrates the calculated concentrations of thebaine in the 500 ppb test sample, utilizing the optimal linear calibration curve with the weighted method as x and applying the bias method to detect outliers. Blue denotes the 500 ppb test sample, while black indicates the other standards.

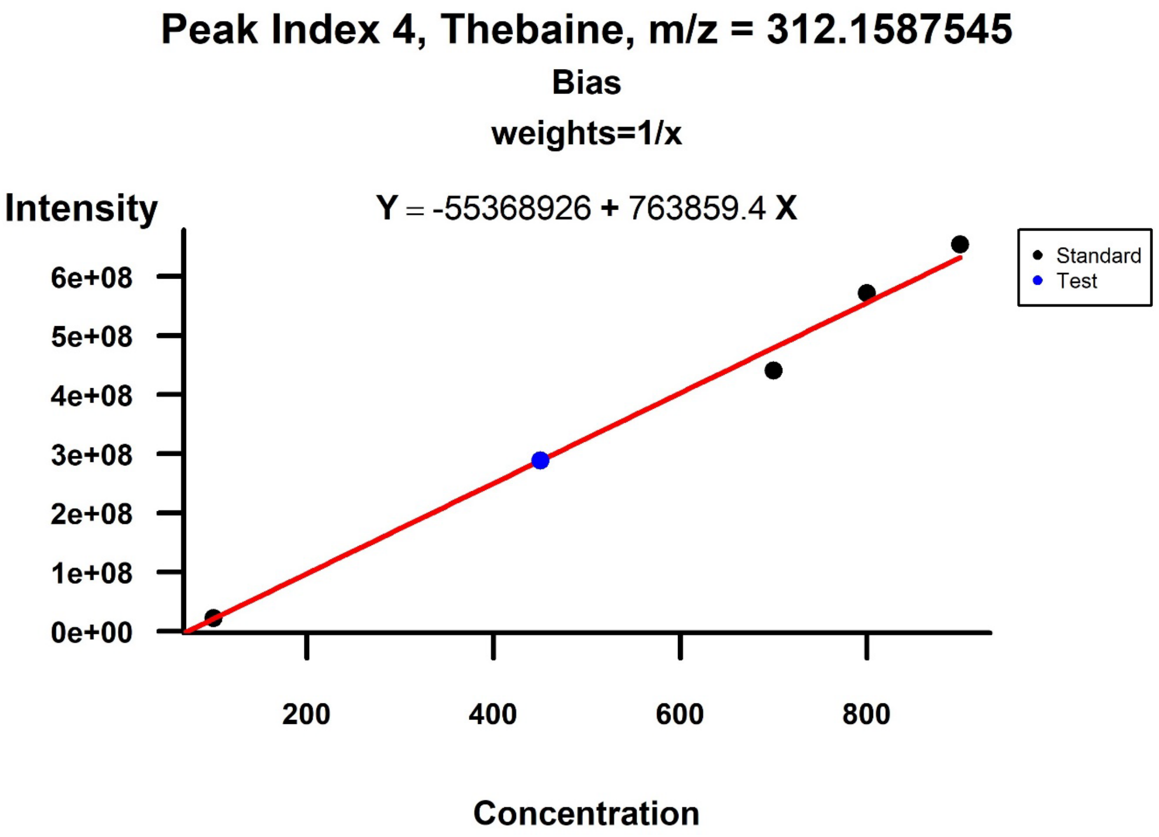

Figure S6D. Optimal calibrated concentration plot for delta9-THC. The graph illustrates the calculated concentrations of delta9-THC in the 500 ppb test sample, utilizing the optimal linear calibration curve with the weighted method as x and applying the bias method to detect outliers. Blue denotes the 500 ppb test sample, while black indicates the other standards.

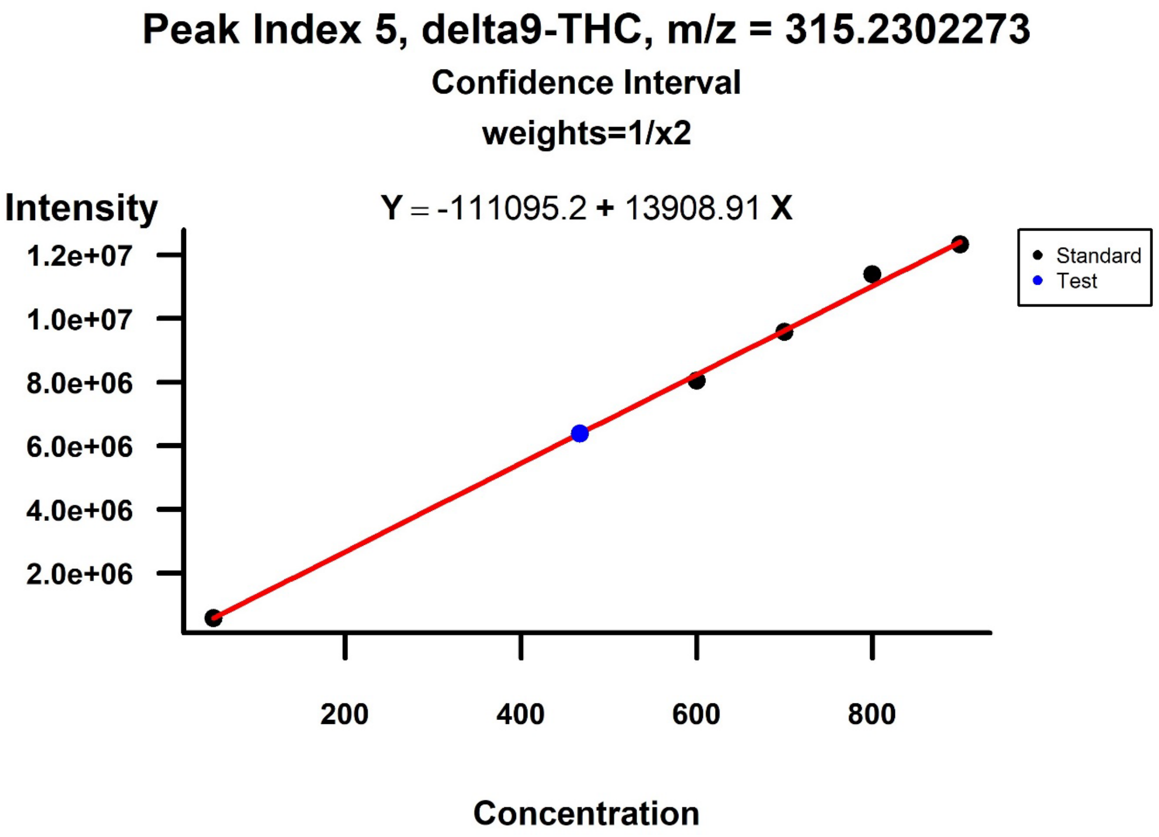

Figure S6E. Optimal calibrated concentration plot for amphetamine. The graph illustrates the calculated concentrations of amphetamine in the 500 ppb test sample, utilizing the optimal linear calibration curve with the weighted method as x and applying the bias method to detect outliers. Blue denotes the 500 ppb test sample, while black indicates the other standards.

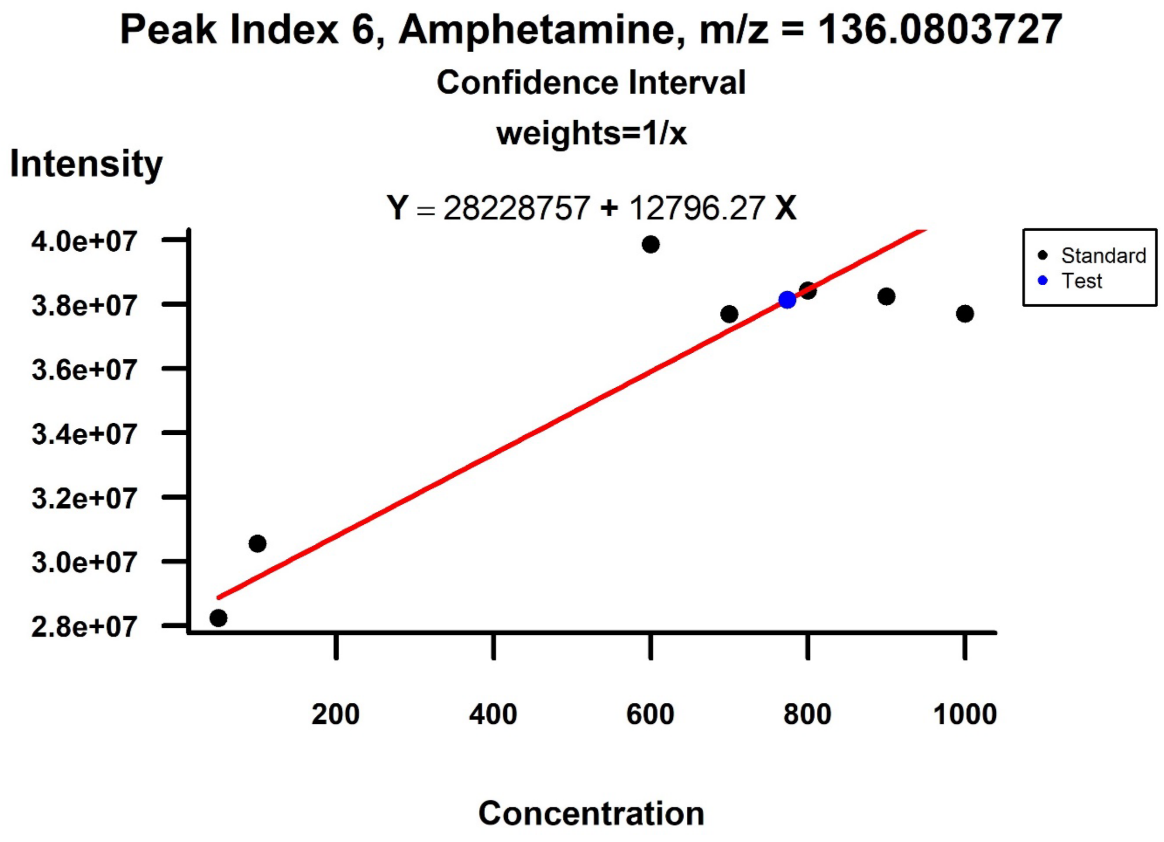

Figure S6F. Optimal calibrated concentration plot for MA. The graph illustrates the calculated concentrations of MA in the 500 ppb test sample, utilizing the optimal linear calibration curve with the weighted method as x and applying the bias method to detect outliers. Blue denotes the 500 ppb test sample, while black indicates the other standards.

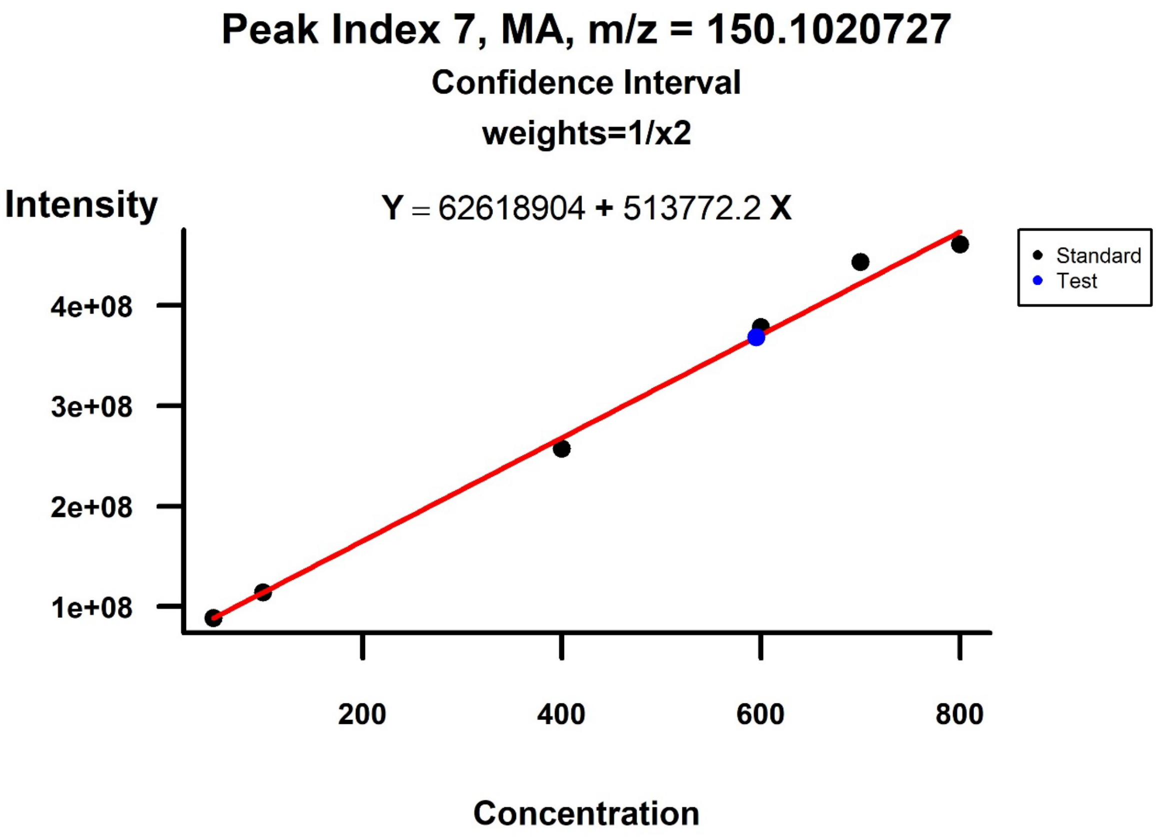

Figure S6G. Optimal calibrated concentration plot for MDMA. The graph illustrates the calculated concentrations of MDMA in the 500 ppb test sample, utilizing the optimal linear calibration curve with the weighted method as x and applying the bias method to detect outliers. Blue denotes the 500 ppb test sample, while black indicates the other standards.

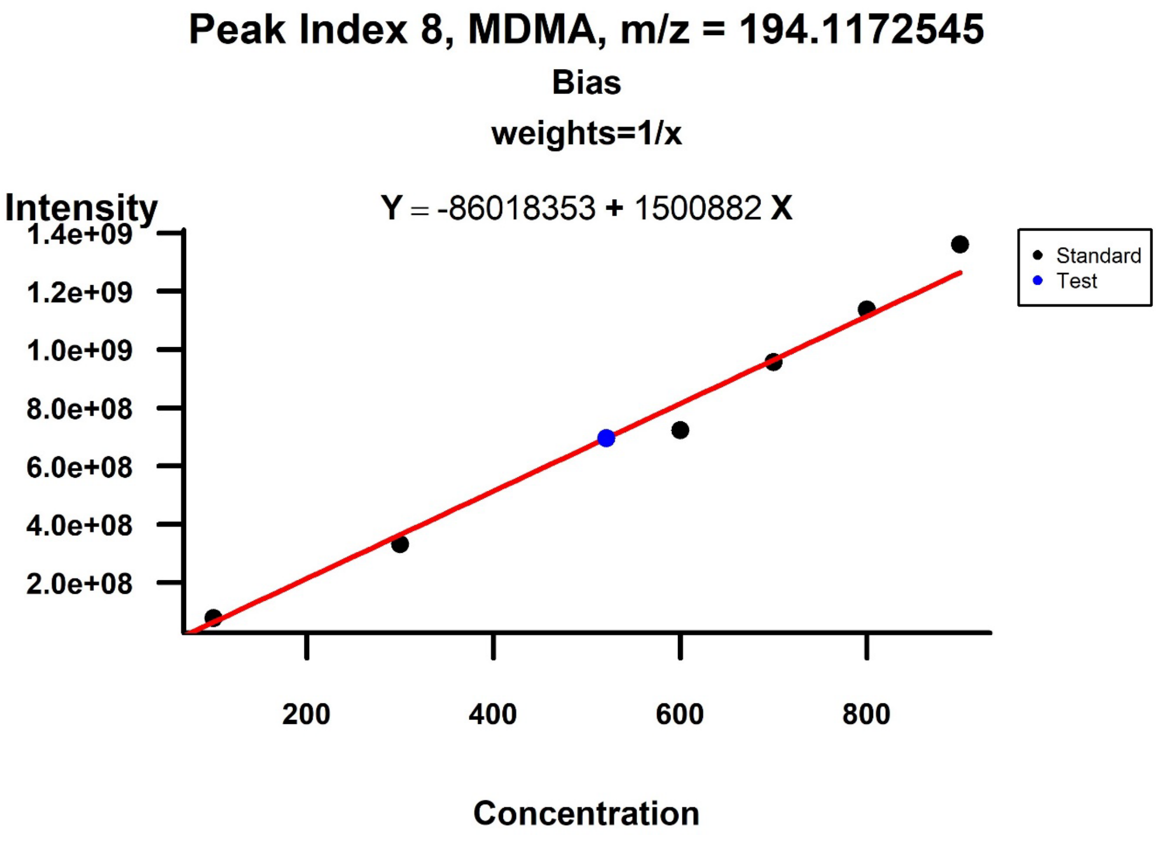

Figure S6H. Optimal calibrated concentration plot for MDA (love drug). The graph illustrates the calculated concentrations of MDA in the 500 ppb test sample, utilizing the optimal linear calibration curve with the weighted method as x and applying the bias method to detect outliers. Blue denotes the 500 ppb test sample, while black indicates the other standards.

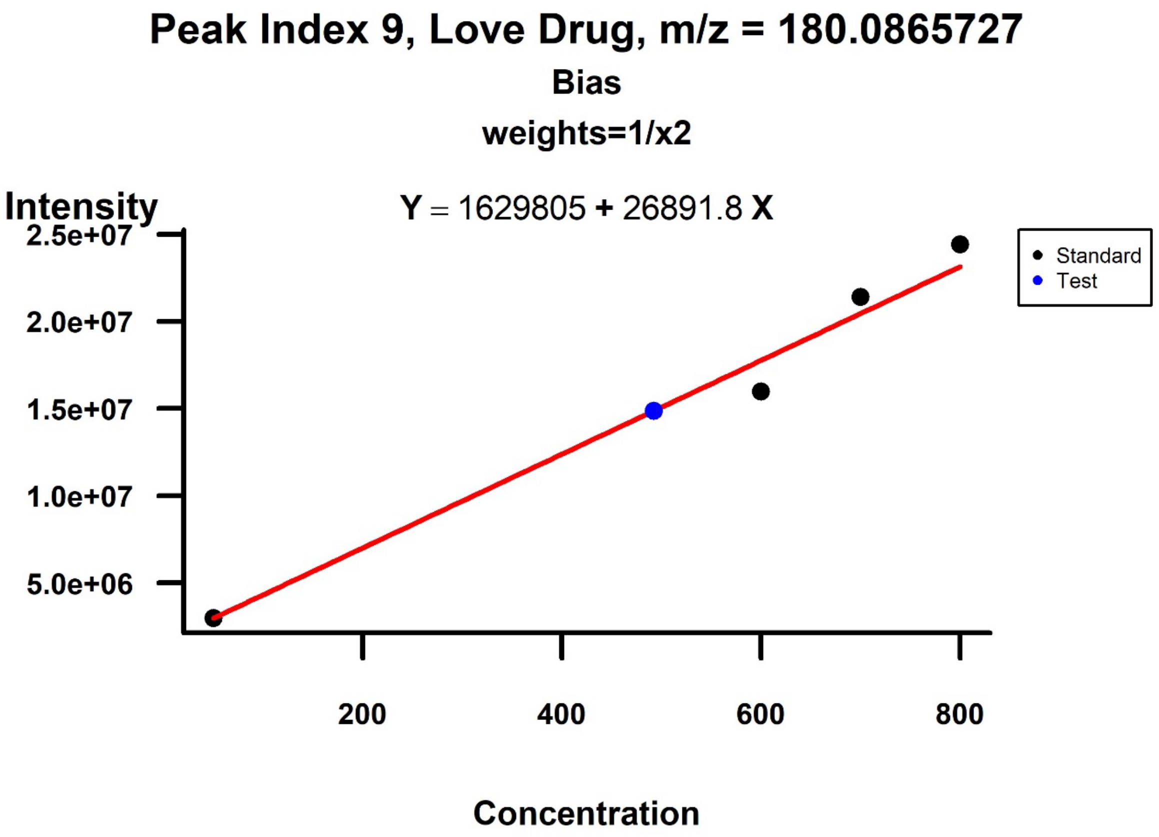

Figure S6I. Optimal calibrated concentration plot for ketamine. The graph illustrates the calculated concentrations of ketamine in the 500 ppb test sample, utilizing the optimal linear calibration curve with the weighted method as x and applying the bias method to detect outliers. Blue denotes the 500 ppb test sample, while black indicates the other standards.

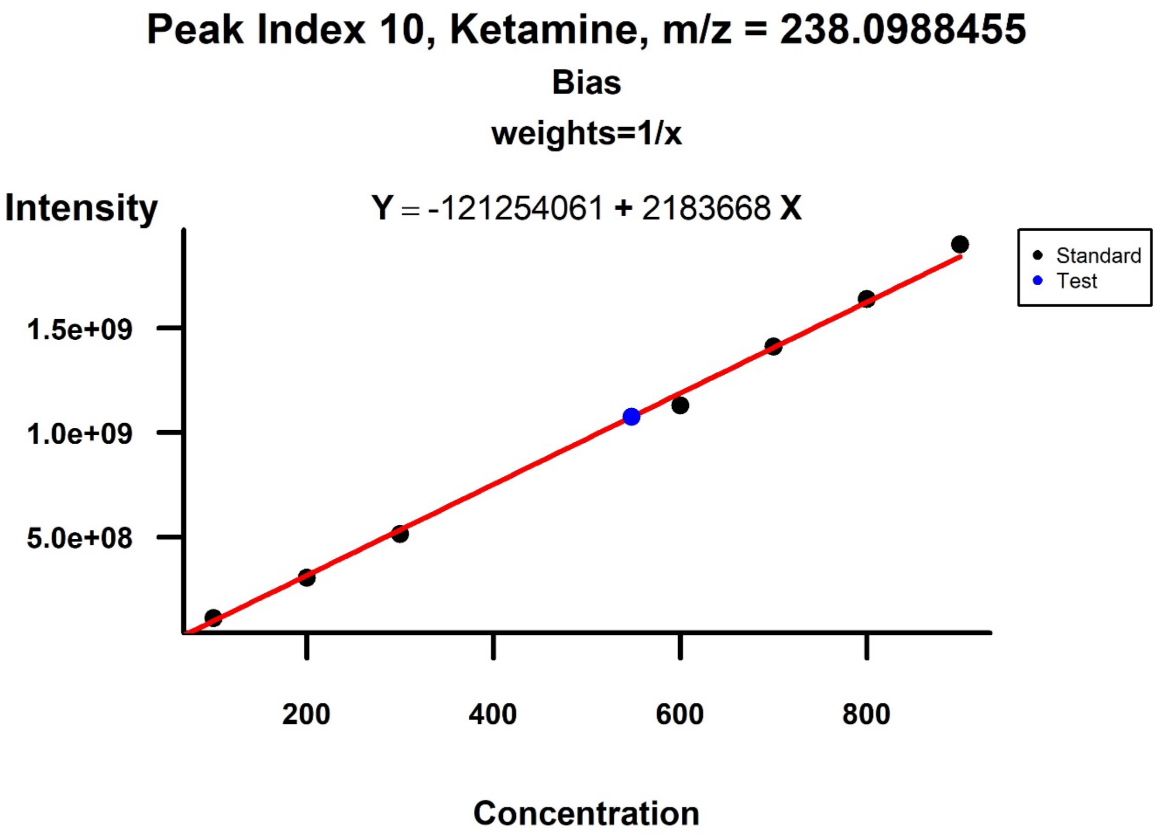

Figure S6J. Optimal calibrated concentration plot for FM2. The graph illustrates the calculated concentrations of FM2 in the 500 ppb test sample, utilizing the optimal linear calibration curve with the weighted method as x and applying the bias method to detect outliers. Blue denotes the 500 ppb test sample, while black indicates the other standards.

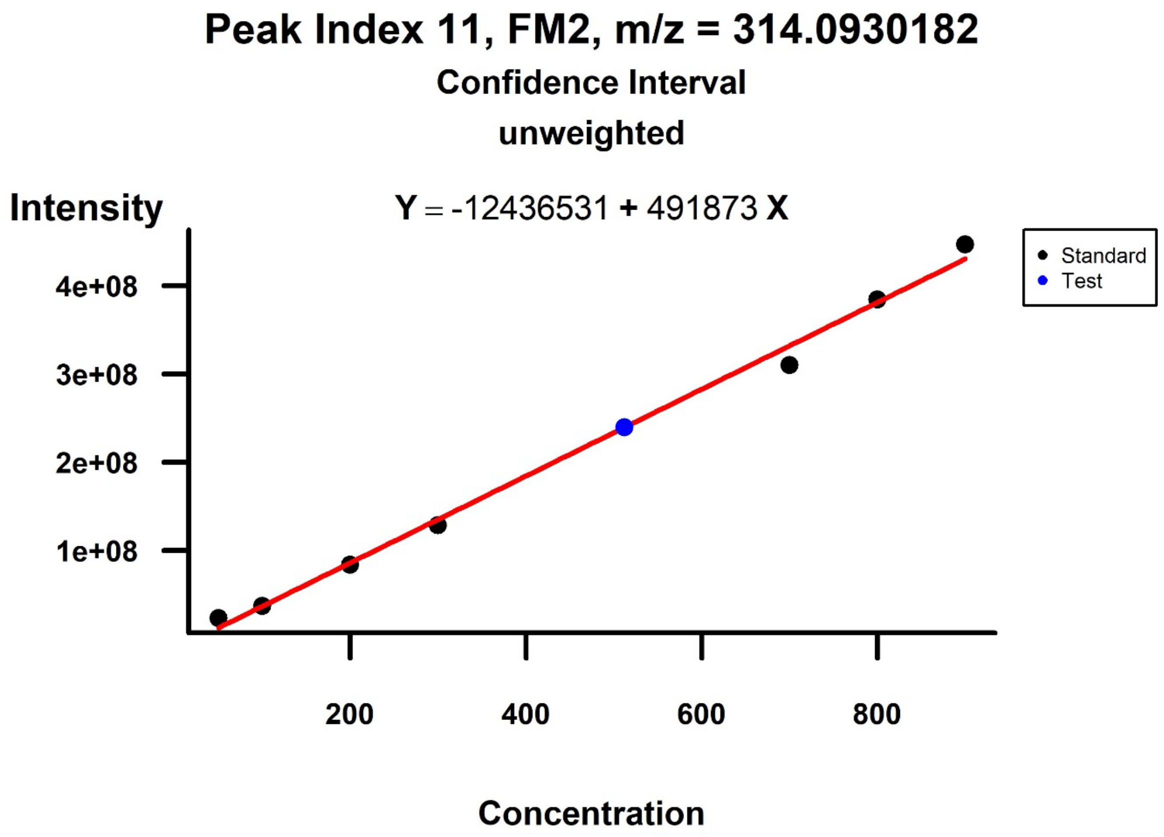

Figure S6K. Optimal calibrated concentration plot for nimetazepam. The graph illustrates the calculated concentrations of nimetazepam in the 500 ppb test sample, utilizing the optimal linear calibration curve with the weighted method as x and applying the bias method to detect outliers. Blue denotes the 500 ppb test sample, while black indicates the other standards.

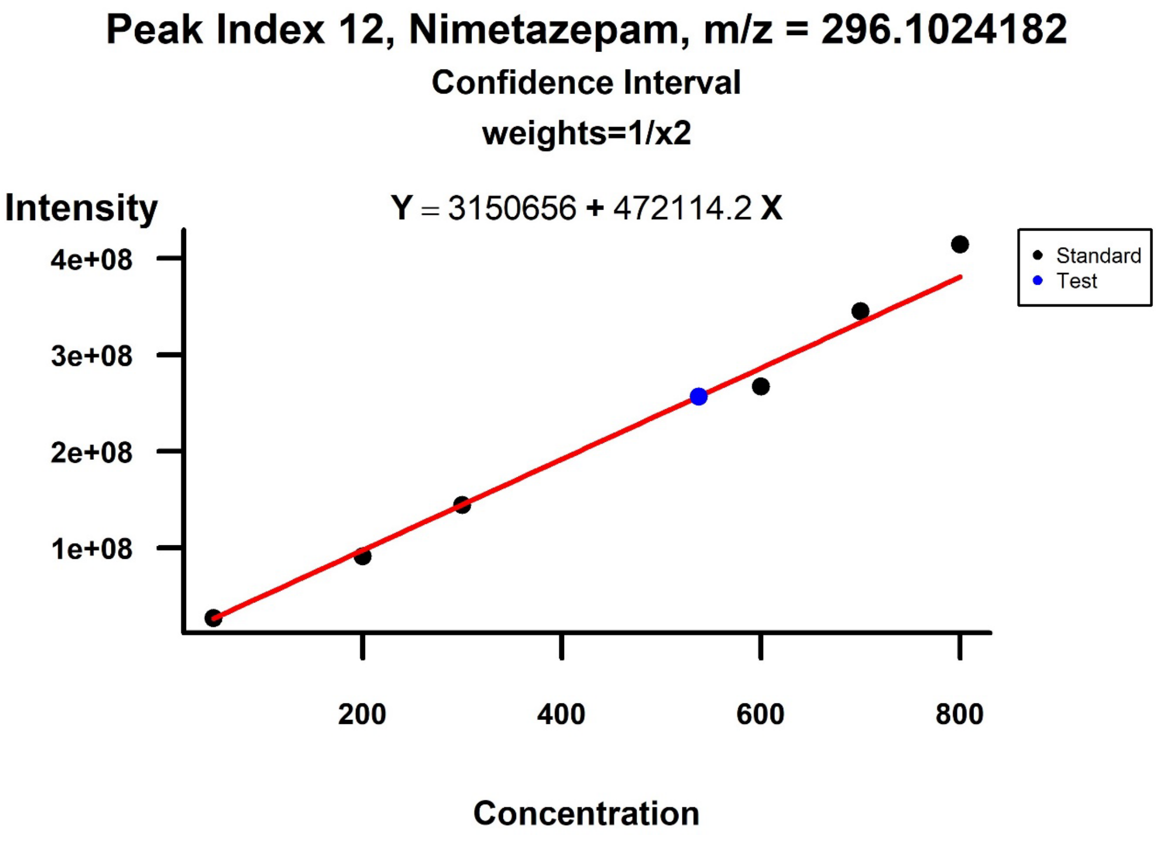

REFERENCES

(1) Smith, C. A.; Want, E. J.; O'Maille, G.; Abagyan, R.; Siuzdak, G. *Anal Chem* **2006**, *78*, 779-787.
(2) Tarca, A. L.; Draghici, S.; Khatri, P.; Hassan, S. S.; Mittal, P.; Kim, J. S.; Kim, C. J.; Kusanovic, J. P.; Romero, R. *Bioinformatics* **2009**, *25*, 75-82.
(3) Tenenbaum, D.; Maintainer, B. *R package version 1.42.0* **2022**.
(4) Zhang, J. D.; Wiemann, S. *Bioinformatics* **2009**, *25*, 1470-1471.
(5) Lin, Y. C.; Liang, Y. J.; Yang, H. C. *Comput Struct Biotec* **2022**, *20*, 3615-3620.
